# One-Pot Synthesis of (*E*)-2-(3-Oxoindolin-2-ylidene)-2-arylacetonitriles

**DOI:** 10.3390/molecules27092808

**Published:** 2022-04-28

**Authors:** Nicolai A. Aksenov, Alexander V. Aksenov, Igor A. Kurenkov, Nikita K. Kirillov, Dmitrii A. Aksenov, Nikolai A. Arutiunov, Daria S. Aksenova, Michael Rubin

**Affiliations:** 1 Department of Chemistry, North Caucasus Federal University, 1a Pushkin St., Stavropol 355017, Russia; aaksenov@ncfu.ru (A.V.A.); kurenkman@icloud.com (I.A.K.); lyncheron@gmail.com (N.K.K.); daksenov@ncfu.ru (D.A.A.); naarutiunov@ncfu.ru (N.A.A.); dasha.severilo@gmail.com (D.S.A.); 2 Department of Chemistry, University of Kansas, 1567 Irving Hill Road, Lawrence, KS 66045, USA

**Keywords:** nitroalkanes, brønsted acid catalysis, indoles, rearrangements, cascade transformations

## Abstract

A highly efficient and expeditious one-pot approach towards 2-(3-oxoindolin-2-yl)acetonitriles was designed, which involves a base-assisted aldol reaction of *ortho*-nitroacetophenones, followed by hydrocyanation, triggering an unusual reductive cyclization reaction.

## 1. Introduction

**Scheme 1:**
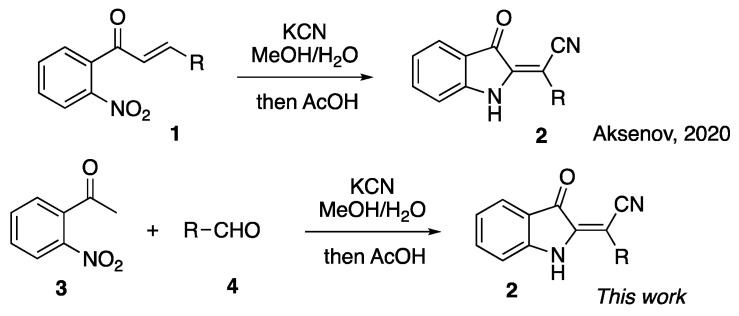
Cyclization of ortho-nitrochalcones into 2-alkylideneindolin-3-ones.

The derivatives of 2-Alkylideneindolin-3-one play important role in modern medicinal chemistry. The bis-indole indirubin known as “Tyrian purple” dye has been used for many years as an active component of a traditional Chinese herbal medicine [1,2,3]. Lately, numerous synthetic analogs of this compound were reported to show potent and highly selective pharmacological inhibition of glycogen synthase kinases and cycline-dependent kinases [4,5,6]. Related compounds possessing a single 3-oxoindoline subunit (or two remotely positioned subunits) also were isolated from natural sources and are also known to exhibit a wide spectrum of important biological properties [7,8,9,10,11,12,13]. Normally, the preparation of these structures relies heavily on the chemistry of isatins, which greatly limits the diversity of accessible substitution patterns. Alternative methods have also been developed based on the aldol condensation of 3*H*-indol-3-ones with carbonyl compounds [14,15,16,17], the transition metal-catalyzed carbonylative cross-coupling of *ortho*-iodoanilines to alkynes [18,19,20,21], or the cascade cyclizations of anilines with α-ketoesters [7,22]. We recently reported an original, facile, and highly efficient method for the preparation of 2-(3-oxoindolin-2-ylidene) acetonitriles **2** from ortho-nitrochalcones **1**, operating as a triggered Michael addition of the cyanide anion to the chalcone followed by a cascade cyclization mechanistically related to the Baeyer–Drewson reaction (Scheme 1) [23].

Herein, we report on the development of a more streamlined synthetic approach which allows for the combining of this transformation with the preparation of the chalcone precursor 1 from aldehydes 4 and *ortho*-nitroacetophenones 3 in a single one-pot operation.

## 2. Results and Discussion

Since our newly developed method for preparation of 2-(3-oxoindolin-2-ylidene) acetonitriles **2** relies heavily on the availability of the corresponding chalchone precursors **1**, we wondered about possibility of generating them in situ via an aldol condensation reaction. It was initially planned that this step could be carried out in the presence of catalytic amounts of alkali, which later would be neutralized with acetic acid to trigger the hydrocyanation step. However, much more attractive was the idea of employing potassium cyanide as a base to catalyze the aldol condensation step. This unusual approach to the aldol reaction was precedented in a single report by Migita et al. [24]. To test this idea, we subjected 2-nitroacetophenone (**2a**) to the reaction with *p*-anisaldehyde (**4a**) in the presence of KCN in methanol (2 mL). Water additive (130 μL) was used, as it was previously demonstrated it is crucial for successful cyclization [23]. The reaction mixture was refluxed for 1 h, then treated with acetic acid (150 μL) and refluxed for an additional 30 min to afford 3-oxoindoline **2aa** in 64% yield (Table 1, entry 1). This reaction was accompanied by the formation of a large number of unidentified side products, as evidenced by TLC analysis. In attempt to improve this situation, we decided to carry out the reaction under conditions ensuring a faster kinetic rate of the desired transformation. First, cyanide loading was increased (with a proportional increase of water and acetic acid concentrations). The reaction proceeded faster, but the yield of the target product was not improved under these conditions (entry 2). Then, the concentration effect was evaluated, with the knowledge that this effect should be favorable for bimolecular reactions. Indeed, when the concentration of starting materials was increased twice, **2aa** was formed cleanly in 95% yield (entry 3). Finally, it was found that decreasing the temperature had a detrimental effect on the reaction performance. The reaction proceeded very sluggishly, affording **2aa** in 58% yield along with several unidentified side products (entry 4). Without water additive, this reaction affords lower yields (entry 5), and this is also the case when other acids are employed instead of acetic (entries 6, 7).

**Scheme 2:**
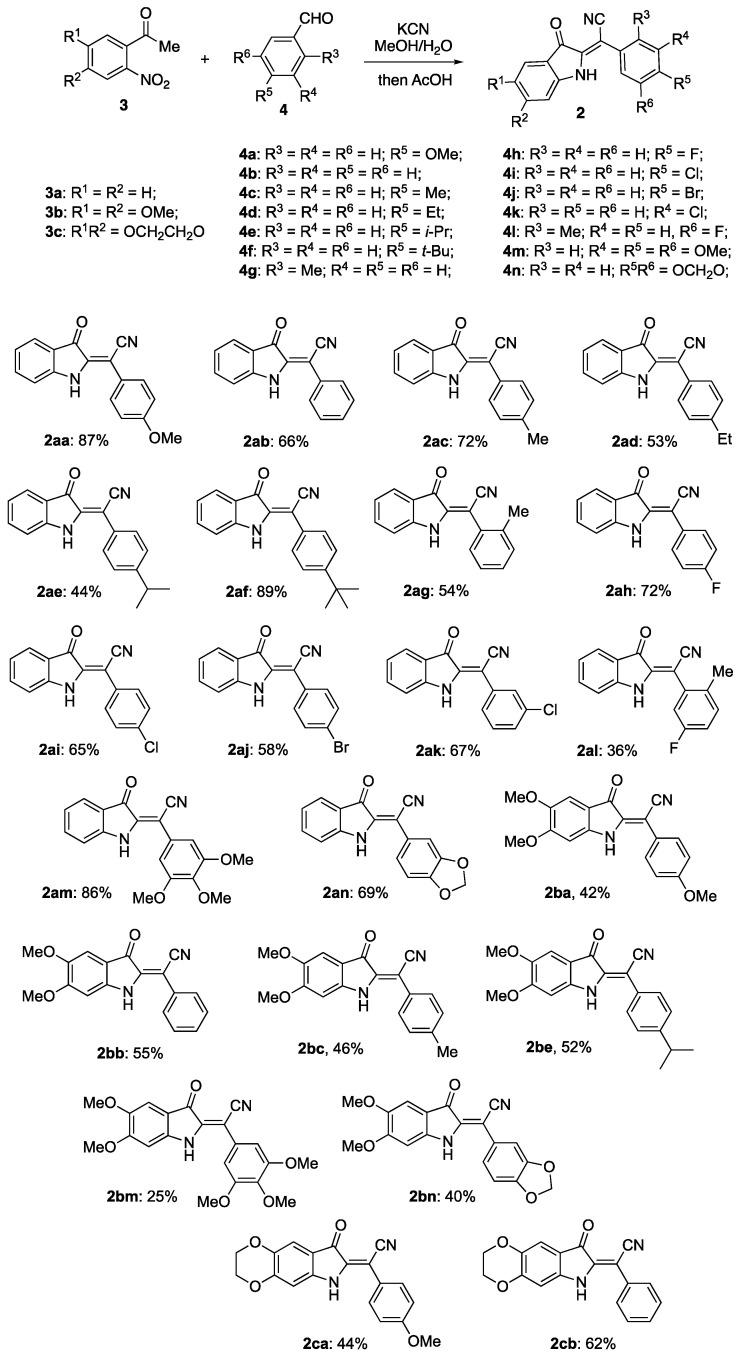
Preparation of 2-(3-oxoindolin-2-yl)acetonitriles **2**.

**Figure 1:**
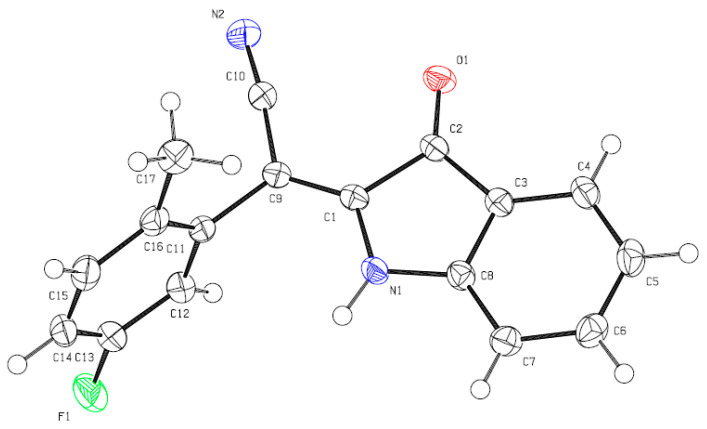
ORTEP drawing of X-Ray structures of (*E*)-2-(5-fluoro-2-methylphenyl)-2-(3-oxoindolin-2-ylidene)acetonitrile (**2al**, CCDC #2157035). The thermal ellipsoids are shown at 50% probability. Green:fluorine; blue:nitrogen; red:oxygen, gray:carbon.

With the optimized conditions in hand, we performed these transformations in a preparative scale (up to 2.00 mmol) and managed to obtain comparably high isolated yields of **2aa** (85%) (Table 1, entry 3, Scheme 2). The reaction demonstrated good tolerance and compatibility with a variety of substituents, including alkoxyarenes, halogenated arenes, and cyclic acetals (Scheme 2). The formation of (*E*)-2-(3-oxoindolin-2-ylidene)acetonitrile moiety in this reaction was unambiguously confirmed by single crystal X-ray diffraction of compound **2al** (CCDC #2157035, Figure 1).

**Scheme 3:**
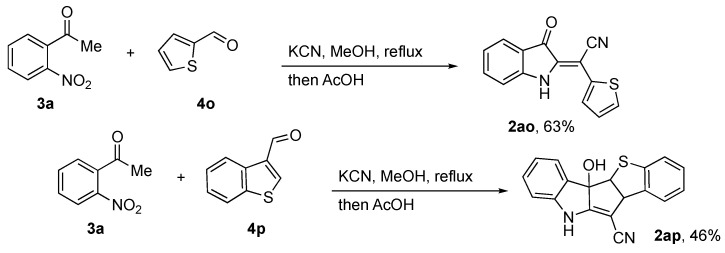
Reactions with thiophenecarbaldehydes **4o**-**p**.

It was possible to engage heterocyclic aldehydes into the featured transformation. Thus, the reaction of *ortho*-nitroacetophenone (**3a**) with thiophene-2-carbaldehyde (**4o**) afforded thienyl-substituted compound **2ao** in good yield (Scheme 3). On the other hand, the reaction involving benzo[*b*]thiophene-3-carbaldehyde (**4p**) led to the formation of rearranged polycyclic product 2ap (Scheme 3). Further investigation of this unusual transformation is currently under way in our laboratories.

**Scheme 4:**
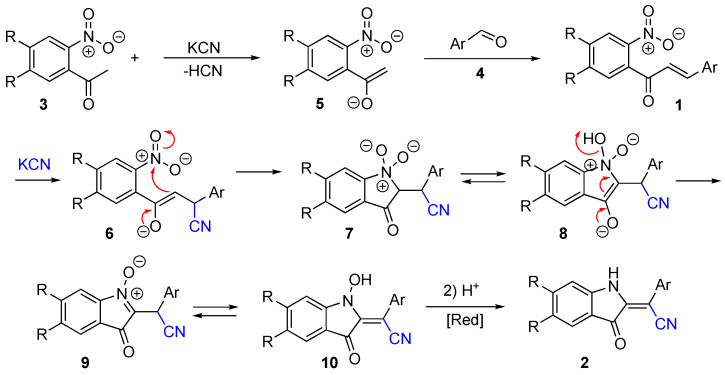
Mechanistic rationale for cascade transformation providing 2-(3-oxoindolin-2-yl)acetonitriles **2**.

**Scheme 5:**
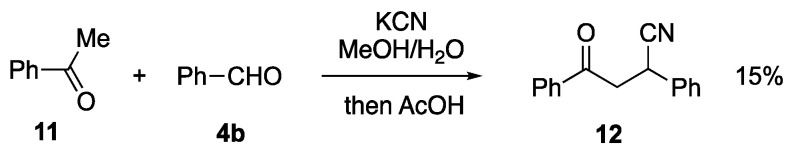
Cyanide-induced Aldol condensation/Michael addition cascade.

**Scheme 6:**
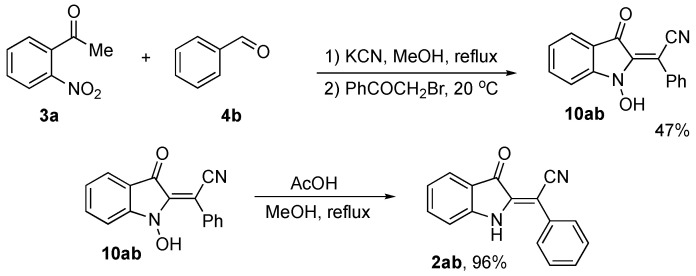
Preparation intermediate cyclic hydroxylamine **10ab** and its further conversion into 2-(3-oxoindolin-2-yl)acetonitrile **2ab**.

The mechanistic rationale for the featured transformation is shown in Scheme 4. It is believed that potassium cyanide can serve as a base to induce the enolization of ortho-nitroacetophenone **3** to provide enolate form **5**, which can be engaged into an aldol reaction with aldehyde **4** affording chalchone **1** (Scheme 4). The subsequent conjugate addition of cyanide to chalchone would provide 4-oxo-4-arylbutanenitrile in enolate form **6**. To demonstrate the possibility of carrying out this sequence of reactions, we tested the reactivity between acetophenone (**11**) and benzaldehyde (**4b**) in the presence of excess potassium cyanide under typical reaction conditions. Expectedly, 4-oxo-4-phenylbutanenitrile **12** was formed smoothly, albeit in marginal yield (Scheme 5). Once formed, enolate species **6** would experience intramolecular nucleophilic attack involving the ortho-nitro group to afford cyclic keto-azinate **7**, which should exist in tautomeric equilibrium with azinic acid-enolate form **8** (Scheme 4). The elimination of the hydroxyl group from this species would lead to the formation of imine *N*-oxide intermediate 9, which should tautomerize into cyclic hydroxylamine **10**. Subsequent reduction (most likely involving methanol as a reducing agent) [25] would afford final 2-(3-oxoindolin-2-yl)acetonitriles **2** (Scheme 4) [23]. It should be pointed out that carrying out a reaction between ortho-nitroacetophenone **3a** and benzaldehyde **4b** followed by quenching with phenacyl bromide (serving as a precursor of HBr, slowly released over an extended period) allowed for the isolation of compound **10ab** in moderate yield (Scheme 6). The same compound was detected by mass spectroscopy in the reaction mixture carried out under standard conditions. It proved to be stable upon heating in DMSO at 100 °C for 2 hr. However, treatment of this sample with water or formic acid caused the slow consumption of **10ab** (mass 285 [M + Na]) and accumulation of product **2ab** (mass 269 [M + Na]). In the presence of methanol and acetic acid, this process proceeded notably faster. In preparative scale, this reaction afforded a nearly quantitative conversion into **2ab**, identical to the sample obtained in one-pot fashion (Scheme 6).

## 3. Conclusions

An improved protocol for the preparation of 2-(3-oxoindolin-2-yl)acetonitriles **2** via a cyanide-mediated cascade reaction of *ortho*-nitroacetophenones (**3**) with aromatic aldehydes (**4**) was developed. This transformation involves an initial aldol condensation followed by a Michael-type conjugated addition of cyanide anion to the intermediate chalcone, which triggers an unusual cyclization mechanistically related to the Baeyer–Drewson reaction. This methodology was employed to synthesize a small, focused library of target molecules. It should be mentioned that aliphatic aldehydes do not participate in aldol condensation under these reaction conditions and cannot be used as precursors for preparation of alkyl-substituted analogs. An investigation into the biological activity of these new €-2-(3-oxoindolin-2-ylidene)-2-arylacetonitriles is currently under way in our laboratories.

## 4. Experimental Part

### General

NMR spectra (^1^H and ^13^C) were measured in solutions of CDCl_3_ or DMSO-*d_6_* on a Bruker AVANCE-III HD instrument (at 400.40 or 100.61 MHz, respectively). HRMS spectra were measured in MeCN solutions on Bruker maXis impact (electrospray ionization, employing HCO_2_Na–HCO_2_H for calibration). See Supplementary Materials for NMR and HRMS spectral charts and X-Ray crystallography data. IR spectra was measured on an FT-IR spectrometer Shimadzu IRAffinity-1S equipped with an ATR sampling module. Reaction progress, purity of isolated compounds, and R*_f_* values were assessed by TLC on Silufol UV-254 plates. Column chromatography was performed on silica gel (32–63 μm, 60 Å pore size). Melting points were measured on Stuart SMP30 apparatus. All reagents and solvents were purchased from commercial vendors and used as received. The reactions involving KCN are accompanied by the formation of highly toxic fumes of HCN. A well-ventilated fume hood must be used.

*Preparation of (E)-2-aryl-2-(3-oxoindolin-2-ylidene)acetonitriles (**2aa**–**2cb**) by reaction of benzaldehydes (**4a**–**n**) and* 2′-*nitroacetophenones (**3a**–**c**) (General procedure):*

A 5-mL round bottom flask equipped with magnetic stirring bar was charged with 2′-nitroacetophenone (1 mmol), benzaldehyde (1 mmol), KCN (2 mmol, 130 mg), H_2_O (130 mg), and MeOH (1 mL) and refluxed for 30 min (TLC control). After the consumption of the starting material, the reaction mixture was cooled to room temperature and AcOH (2.5 mmol, 150 mg, 143 µL) (exothermic reaction) was added and reflux was continued to another 30 min. Then, the reaction mixture was diluted with 100 mL of EtOAc and washed twice with 30 mL of concentrated NaHCO_3_ solution. The organic layer was concentrated and purified by column chromatography (gradient: EtOAc:Hex 1:4–1:1) or by recrystallization from EtOH.

*(E)-2-(4-methoxyphenyl)-2-(3-oxoindolin-2-ylidene)acetonitrile* (**2aa**): 

Red solid, mp (EtOH) 247.5–249.4 °C (Literature data: [23] mp 250.1–251.1 °C), R*_f_* 0.23 (EtOAc/Hex, 1:2). Yield: 240 mg (0.87 mmol, 87%). ^1^H NMR (400 MHz, DMSO-d_6_) δ 10.42 (s, 1H), 7.61 (dt, *J* = 16.6, 7.9 Hz, 4H), 7.11 (dd, *J* = 15.3, 8.2 Hz, 3H), 7.01 (t, *J* = 7.5 Hz, 1H), 3.84 (s, 3H).

*(E)-2-(3-oxoindolin-2-ylidene)-2-phenylacetonitrile* (**2ab**): 

Orange solid, mp 235.0–236.3 °C (Literature data: [23] mp 233.1–235.9 °C), R*_f_* 0.32 (EtOAc/Hex, 1:2). Yield: 162 mg (0.66 mmol, 66%). ^1^H NMR (400 MHz, DMSO-d_6_) δ 10.52 (s, 1H), 7.72–7.52 (m, 6H), 7.48 (t, *J* = 7.4 Hz, 1H), 7.09 (d, *J* = 8.1 Hz, 1H), 7.03 (t, *J* = 7.5 Hz, 1H). 

*(E)-2-(3-oxoindolin-2-ylidene)-2-(p-tolyl)acetonitrile* (**2ac**): 

Orange solid, mp (MeOH) 241.2–242.6 °C (literature data: [23] mp 236– 240 °C), R*_f_* 0.46 (EtOAc/Hex, 1:2). Yield: 187 mg (0.72 mmol, 72%). ^1^H NMR (400 MHz, DMSO-d_6_) δ 10.45 (s, 1H), 7.65 (d, *J* = 7.6 Hz, 1H), 7.56 (dd, *J* = 16.0, 7.5 Hz, 3H), 7.38 (d, *J* = 8.0 Hz, 2H), 7.09 (d, *J* = 8.1 Hz, 1H), 7.02 (t, *J* = 7.4 Hz, 1H), 2.38 (s, 3H). 

*(E)-2-(4-ethylphenyl)-2-(3-oxoindolin-2-ylidene)acetonitrile* (**2ad**): 

Red solid, mp (EtOH) 214.4–215.1 °C (literature data: [23] mp 223.2–225.7 °C), R*_f_* 0.56 (EtOAc/Hex, 1:2). Yield: 145 mg (0.53 mmol, 53%). ^1^H NMR (400 MHz, DMSO-d_6_) δ 10.47 (s, 1H), 7.65 (d, *J* = 9.8 Hz, 1H), 7.57 (d, *J* = 5.1 Hz, 3H), 7.42 (d, *J* = 8.3 Hz, 2H), 7.10 (d, *J* = 8.1 Hz, 1H), 7.03 (t, 1H), 2.67 (q, 2H), 1.22 (t, 3H). 

*(E)-2-(4-isopropylphenyl)-2-(3-oxoindolin-2-ylidene)acetonitrile* (**2ae**): 

Red solid, mp (EtOH) 223.4–224.6 °C (literature data: [23] mp 223.0–226.6 °C), R*_f_* 0.47 (EtOAc/Hex, 1:2). Yield: 127 mg (0.44 mmol, 44%). ^1^H NMR (400 MHz, DMSO-d_6_) δ 10.48 (s, 1H), 7.65 (d, *J* = 8.4 Hz, 1H), 7.62–7.53 (m, 3H), 7.45 (d, *J* = 8.3 Hz, 2H), 7.09 (d, *J* = 8.1 Hz, 1H), 7.02 (t, *J* = 7.5 Hz, 1H), 2.97 (hept, *J* = 7.0 Hz, 1H), 1.24 (d, *J* = 7.0 Hz, 6H). 

*(E)-2-(4-(tert-butyl)phenyl)-2-(3-oxoindolin-2-ylidene)acetonitrile* (**2af**): 

Red solid, mp 241–243 °C, R*_f_* 0.57 (EtOAc/Hex, 1:1). Yield: 269 mg (0.89 mmol, 89%). ^1^H NMR (400 MHz, DMSO-d_6_) δ 10.49 (s, 1H), 7.65 (d, *J* = 7.6 Hz, 1H), 7.59 (s, 5H), 7.10 (d, *J* = 8.0 Hz, 1H), 7.02 (t, *J* = 7.4 Hz, 1H), 1.33 (s, 9H). ^13^C NMR (101 MHz, DMSO-d_6_) δ 184.2, 152.5, 151.9, 142.2, 137.5, 129.3, 128.6 (2C), 126.3 (2C), 124.9, 121.4, 119.5, 118.0, 112.8, 89.1, 34.7, 31.0 (3C). IR, v_max_/cm^−1^: 3328, 2966, 2206, 1709, 1586, 1470, 1329, 1278, 1222, 1099, 969. HRMS (ES TOF) calculated for (M + Na)^+^ C_20_H_18_N_2_NaO 325.1311, found 325.1309 (0.6 ppm).

*(E)-2-(3-oxoindolin-2-ylidene)-2-(o-tolyl)acetonitrile* (**2ag**)

Red crystals, mp (MeOH) 205.3–206.6 °C (literature data: [23] mp 201.8–203.5 °C), R*_f_* 0.29 (EtOAc/Hex, 1:2). Yield: 140 mg (0.54 mmol, 54%). ^1^H NMR (400 MHz, DMSO-d_6_) δ 10.06 (s, 1H), 7.64 (d, *J* = 7.6 Hz, 1H), 7.55 (t, *J* = 7.6 Hz, 1H), 7.41 (dt, *J* = 7.6, 3.2 Hz, 3H), 7.35 (ddd, *J* = 7.9, 5.2, 2.7 Hz, 1H), 7.03–6.94 (m, 2H), 2.31 (s, 3H). 

*(E)-2-(4-fluorophenyl)-2-(3-oxoindolin-2-ylidene)acetonitrile* (**2ah**): 

Orange solid, mp (EtOH) 279.3–281.4 °C (literature data: [23] mp 281.1–282.8 °C), R*_f_* 0.54 (EtOAc/Hex, 1:2). Yield: 190 mg (0.72 mmol, 72%). ^1^H NMR (400 MHz, DMSO-d_6_) δ 10.50 (s, 1H), 7.74–7.63 (m, 3H), 7.58 (t, *J* = 7.7 Hz, 1H), 7.42 (t, *J* = 8.7 Hz, 2H), 7.08 (d, *J* = 8.1 Hz, 1H), 7.03 (t, *J* = 7.5 Hz, 1H). ^19^F NMR (376 MHz, DMSO-d_6_) δ -111.4. 

*(E)-2-(4-chlorophenyl)-2-(3-oxoindolin-2-ylidene)acetonitrile* (**2ai**): 

Red solid, mp 286–288 °C (literature data: [23] mp 285.9–287.8 °C), R*_f_* 0.22 (EtOAc/Hex, 1:2). Yield: 182 mg (0.65 mmol, 65%). ^1^H NMR (400 MHz, DMSO-d_6_) δ 10.55 (s, 1H), 7.68–7.62 (m, 5H), 7.61–7.56 (m, 1H), 7.08 (d, *J* = 8.0 Hz, 1H), 7.04 (t, *J* = 7.5 Hz, 1H). 

*(E)-2-(4-bromophenyl)-2-(3-oxoindolin-2-ylidene)acetonitrile* (**2aj**): 

Red solid, mp (EtOH) 283.0–284.8 °C (literature data: [23] mp 278.8–282.5 °C), R*_f_* 0.66 (EtOAc/Hex, 1:2). Yield: 187 mg (0.58 mmol, 58%). ^1^H NMR (400 MHz, DMSO-d_6_) δ 10.56 (s, 1H), 7.77 (d, *J* = 8.7 Hz, 2H), 7.65 (d, *J* = 7.2 Hz, 1H), 7.61–7.56 (m, 3H), 7.10–6.99 (m, 2H). 

*(E)-2-(3-chlorophenyl)-2-(3-oxoindolin-2-ylidene)acetonitrile* (**2ak**): 

Orange solid, mp 270–271 °C (literature data: [23] mp 267–269 °C), R*_f_* 0.57 (EtOAc/Hex, 1:2). Yield: 188 mg (0.67 mmol, 67%). ^1^H NMR (400 MHz, DMSO-d_6_) δ 10.63 (s, 1H), 7.66 (d, *J* = 7.4 Hz, 2H), 7.62–7.52 (m, 4H), 7.09 (d, *J* = 8.0 Hz, 1H), 7.04 (t, *J* = 7.5 Hz, 1H). 

*(E)-2-(5-fluoro-2-methylphenyl)-2-(3-oxoindolin-2-ylidene)acetonitrile* (**2al**)

Red crystals, mp (EtOH) 236.1–237.4 °C, R*_f_* 0.22 (EtOAc/Hex, 1:2). Yield: 100 mg (0.36 mmol, 36%). ^1^H NMR (400 MHz, DMSO-d_6_) δ 10.17 (s, 1H), 7.65 (dd, *J* = 7.6, 1.2 Hz, 1H), 7.56 (ddd, *J* = 8.4, 7.3, 1.3 Hz, 1H), 7.44 (dd, *J* = 8.5, 5.9 Hz, 1H), 7.33–7.23 (m, 2H), 7.05–6.94 (m, 2H), 2.28 (s, 3H). ^13^C NMR (101 MHz, DMSO-d_6_) δ 184.1, 160.6 (d, *J* = 243.2 Hz), 152.5, 144.3, 137.8, 133.2 (d, *J* = 3.3 Hz), 132.7 (d, *J* = 8.1 Hz), 132.3 (d, *J* = 8.4 Hz), 125.1, 121.5, 119.6, 117.2, 116.9 (d, *J* = 22.2 Hz), 116.4 (d, *J* = 20.8 Hz), 112.4, 86.3, 18.5. ^19^F NMR (376 MHz, DMSO-d_6_) δ -116.2. IR, v_max_/cm^−1^: 3372, 2206, 1713, 1608, 1482, 1463, 1333, 1224, 176, 1075, 1003, 858, 751. HRMS (ES TOF) calculated for (M + Na)^+^ C_17_H_11_FN_2_NaO 301.0745, found 301.0748 (0.8 ppm).

*(E)-2-(3-oxoindolin-2-ylidene)-2-(3,4,5-trimethoxyphenyl)acetonitrile* (**2am**): 

Red solid, mp 216–218°C, R*_f_* 0.29 (EtOAc/Hex, 1:1). Yield: 289 mg (0.86 mmol, 86%). ^1^H NMR (400 MHz, DMSO-d_6_) δ 10.49 (s, 1H), 7.65 (d, *J* = 7.6 Hz, 1H), 7.60–7.55 (m, 1H), 7.08 (d, *J* = 8.0 Hz, 1H), 7.02 (t, *J* = 7.4 Hz, 1H), 6.89 (s, 2H), 3.86 (s, 6H), 3.73 (s, 3H). ^13^C NMR (101 MHz, DMSO-d_6_) δ 184.1, 153.3 (2C), 152.5, 142.5, 138.1, 137.5, 127.4, 124.9, 121.4, 119.5, 117.9, 112.7, 106.4 (2C), 89.2, 60.1, 56.0 (2C). IR, v_max_/cm^−1^: 3745, 3622, 3272, 2978, 2214, 1737, 1713, 1560, 1506, 1459, 1421, 1274, 1218, 1130, 993, 751. HRMS (ES TOF) calculated for (M + Na)^+^ C_19_H_16_N_2_NaO_4_ 359.1002, found 359.1004 (−0.6 ppm).

*(E)-2-(benzo[d][1,3]dioxol-5-yl)-2-(3-oxoindolin-2-ylidene)acetonitrile* (**2an**): 

Red solid, mp (EtOH) 247.3–248.6 °C (literature data: [23] mp 247.9–248.7 °C), R*_f_* 0.62 (EtOAc/Hex, 1:2). Yield: 200 mg (0.69 mmol, 69%). ^1^H NMR (400 MHz, DMSO-d_6_) δ 10.42 (s, 1H), 7.64 (d, *J* = 7.7 Hz, 1H), 7.60–7.51 (m, 1H), 7.18 (d, *J* = 1.7 Hz, 1H), 7.15–7.07 (m, 3H), 7.01 (t, *J* = 7.5 Hz, 1H), 6.14 (s, 2H). 

*(Z)-2-(3-oxoindolin-2-ylidene)-2-(thiophen-2-yl)acetonitrile* (**2ao**): 

Red solid, mp (EtOH) 229–230 °C, R*_f_* 0.71 (EtOAc/Hex, 1:2). Yield: 159 mg (0.63 mmol, 63%). 1H NMR (400 MHz, DMSO-d_6_) δ 10.39 (s, 1H), 7.86 (d, *J* = 5.4 Hz, 1H), 7.65–7.52 (m, 3H), 7.29 (t, *J* = 4.7 Hz, 1H), 7.20 (d, *J* = 8.2 Hz, 1H), 7.02 (t, *J* = 7.5 Hz, 1H). ^13^C NMR (101 MHz, DMSO-d_6_) δ 184.0, 152.1, 139.9, 137.3, 135.2, 129.43, 129.40, 129.1, 124.8, 121.9, 119.7, 117.1, 113.3, 84.6. IR, v_max_/cm^−1^ 3320, 2214, 1705, 1685, 1624, 1584, 1552, 1528, 1480, 1466, 1421, 1391, 1363, 1299, 1254, 1198. HRMS (ES TOF) calculated for (M + Na)^+^ C_14_H_8_N_2_NaOS 275.0250, found 275.0250 (−0.1 ppm).

*5b-Hydroxy-5b,10-dihydrobenzo [4’,5’]thieno [2’,3’:3,4]cyclopenta [1,2-b]indole-11-carbonitrile* (**2ap**):

Red solid, mp (EtOH) 218–219 °C, R*_f_* 0.37 (EtOAc/Hex, 1:2). Yield: 139 mg (0.46 mmol, 46%). ^1^H NMR (400 MHz, DMSO-d6) δ 12.04 (s, 1H), 11.39 (s, 1H), 9.00–8.90 (m, 1H), 7.77 (d, *J* = 8.1 Hz, 1H), 7.70 (d, *J* = 8.4 Hz, 1H), 7.46–7.40 (m, 1H), 7.39–7.30 (m, 3H), 7.15 (t*, J* = 7.5 Hz, 1H).^13^C NMR (101 MHz, DMSO) δ 160.3, 159.5, 138.8, 137.4, 131.0, 130.7, 129.1, 128.4, 126.3, 126.2, 125.8, 125.4, 120.3, 117.7, 116.7, 116.0, 113.7, 113.4. IR, v_max_/cm^−1^ 3316, 3049, 2210, 1679, 1624, 1584, 1550, 1530, 1504, 1463, 1387, 1321. HRMS (ES TOF) calculated for (M + Na)^+^ C_18_H_10_N_2_NaO 325.0406, found 325.0404 (0.7 ppm).

*(E)-2-(5,6-dimethoxy-3-oxoindolin-2-ylidene)-2-(4-methoxyphenyl)acetonitrile* (**2ba**): 

Red solid, mp (EtOH) 270.6–272.1 °C, R*_f_* 0.2 (EtOAc/Hex, 3:2). Yield: 140 mg (0.42 mmol, 42%). ^1^H NMR (400 MHz, DMSO-d_6_) δ 10.05 (s, 1H), 7.57 (d, *J* = 8.9 Hz, 2H), 7.19–7.05 (m, 3H), 6.63 (s, 1H), 3.85 (d, *J* = 8.8 Hz, 6H), 3.75 (s, 3H). ^13^C NMR (101 MHz, DMSO-d_6_) δ 181.9, 159.7, 157.7, 150.1, 144.8, 143.0, 130.3 (2C), 124.2, 118.1, 114.8 (2C), 110.5, 105.7, 95.8, 89.0, 56.1, 56.0, 55.5. IR, _vmax_ /cm^−1^: 3332, 2203, 1684, 1596, 1493, 1354, 1246, 1191, 1135, 1033. HRMS (ES TOF) calculated for (M + Na)^+^ C_19_H_16_N_2_NaO_4_ 359.1002, found 359.0993 (2.7 ppm).

*(E)-2-(5,6-dimethoxy-3-oxoindolin-2-ylidene)-2-phenylacetonitrile* (**2bb**): 

Purple solid, mp (EtOH) 204.9–206.6 °C (literature data: [23] mp 205.2–207.6 °C), R*_f_* 0.47 (EtOAc/Hex, 1:1). Yield: 168 mg (0.55 mmol, 55%). ^1^H NMR (400 MHz, DMSO-d_6_) δ 10.15 (s, 1H), 7.64–7.60 (m, 2H), 7.56 (t, *J* = 7.7 Hz, 2H), 7.49–7.45 (m, 1H), 7.09 (s, 1H), 6.63 (s, 1H), 3.86 (s, 3H), 3.76 (s, 3H). 

*(E)-2-(5,6-dimethoxy-3-oxoindolin-2-ylidene)-2-(p-tolyl)acetonitrile* (**2bc**): 

Red solid, mp (EtOH) 281.6–282.7 °C, R*_f_* 0.3 (EtOAc/Hex, 3:2). Yield: 148 mg (0.46 mmol, 46%). ^1^H NMR (400 MHz, DMSO-d_6_) δ 10.09 (s, 1H), 7.52 (d, *J* = 8.2 Hz, 2H), 7.36 (d, *J* = 8.3 Hz, 2H), 7.07 (s, 1H), 6.62 (s, 1H), 3.85 (s, 3H), 3.75 (s, 3H), 2.38 (s, 3H). ^13^C NMR (101 MHz, DMSO-d_6_) δ 181.9, 157.7, 150.2, 144.8, 143.5, 138.8, 129.9 (2C), 129.3, 128.7 (2C), 118.1, 110.4, 105.8, 95.8, 88.8, 56.1, 55.9, 20.9. IR, v_max_ /cm^−1^: 3273, 2207, 1688, 1598, 1485, 1360, 1202, 1169, 1129, 1081. HRMS (ES TOF) calculated for (M + Na)^+^ C_19_H_16_N_2_NaO_3_ 343.1053, found 343.1046 (2.2 ppm).

*(E)-2-(5,6-dimethoxy-3-oxoindolin-2-ylidene)-2-(4-isopropylphenyl)acetonitrile* (**2be**): 

Red solid, mp (EtOH) 201.4–202.2 °C, R*_f_* 0.4 (EtOAc/Hex, 3:2). Yield: 181 mg (0.52 mmol, 52%). ^1^H NMR (400 MHz, DMSO-d_6_) δ 10.12 (s, 1H), 7.55 (d, *J* = 8.3 Hz, 2H), 7.43 (d, *J* = 8.4 Hz, 2H), 7.08 (s, 1H), 6.63 (s, 1H), 3.85 (s, 3H), 3.75 (s, 3H), 2.96 (hept, *J* = 6.9 Hz, 1H), 1.24 (d, *J* = 6.9 Hz, 6H). ^13^C NMR (101 MHz, DMSO-d_6_) δ 182.0, 157.7, 150.2, 149.6, 144.8, 143.6, 129.7, 128.8 (2C), 127.3 (2C), 118.1, 110.4, 105.8, 95.9, 88.7, 56.1, 56.0, 33.4, 23.7 (2C). IR, v_max_ /cm^−1^: 3293, 2211, 1682, 1598, 1491, 1358, 1324, 1202, 1167, 1135. HRMS (ES TOF) calculated for (M + Na)^+^ C_21_H_20_N_2_NaO_3_ 371.1366, found 371.1360 (1.7 ppm).

*(E)-2-(5,6-dimethoxy-3-oxoindolin-2-ylidene)-2-(3,4,5-trimethoxyphenyl)acetonitrile* (**2bm**): 

Purple solid, mp (EtOH) 182.1–184.0 °C, R*_f_* 0.17 (EtOAc/Hex, 3:2). Yield: 99 mg (0.25 mmol, 25%). ^1^H NMR (400 MHz, DMSO-d_6_) δ 10.13 (s, 1H), 7.08 (s, 1H), 6.86 (s, 2H), 6.61 (s, 1H), 3.86 (s, 9H), 3.74 (d, *J* = 10.8 Hz, 6H). ^13^C NMR (101 MHz, DMSO-d_6_) δ 181.8, 157.8, 153.3 (2C), 150.2, 144.8, 143.9, 138.0, 127.5, 117.9, 110.4, 106.3 (2C), 105.8, 95.8, 88.8, 60.1, 56.1, 56.0 (3C). IR, v_max_ /cm^−1^: 3285, 2207, 1670, 1600, 1493, 1340, 1310, 1212, 1125, 996. HRMS (ES TOF) calculated for (M + Na)^+^ C_21_H_20_N_2_NaO_6_ 419.1214, found 419.1208 (1.3 ppm).

*(E)-2-(benzo[d][1,3]dioxol-5-yl)-2-(5,6-dimethoxy-3-oxoindolin-2-ylidene)acetonitrile* (**2bn**): 

Red solid, mp (EtOH) 240.7–242.7 °C, R*_f_* 0.24 (EtOAc/Hex, 3:2). Yield: 140 mg (0.40 mmol, 40%). ^1^H NMR (400 MHz, DMSO-d_6_) δ 10.08 (s, 1H), 7.16 (s, 1H), 7.15–7.08 (m, 2H), 7.06 (s, 1H), 6.63 (s, 1H), 6.14 (s, 2H), 3.86 (s, 3H), 3.75 (s, 3H). ^13^C NMR (101 MHz, DMSO-d_6_) δ 181.9, 157.7, 150.2, 147.9 (2C), 144.8, 143.4, 125.8, 123.4, 118.1, 110.5, 109.1, 108.8, 105.8, 101.8, 95.8, 88.7, 56.1, 56.0. IR, v_max_ /cm^−1^: 3297, 2211, 1686, 1592, 1487, 1364, 1316, 1248, 1202, 1173, 1135. HRMS (ES TOF) calculated for (M + Na)^+^ C_19_H_14_N_2_NaO_5_ 373.0795, found 373.0795 (2.8 ppm).

*(E)-2-(4-methoxyphenyl)-2-(8-oxo-2,3,6,8-tetrahydro-7H-[1,4]dioxino [2,3-f]indol-7-ylidene)acetonitrile* (**2ca**): 

Red solid, mp (EtOH) 269.8–271.9 °C, R*_f_* 0.32 (EtOAc/Hex, 1:1). Yield: 147 mg (0.44 mmol, 44%). ^1^H NMR (400 MHz, DMSO-d_6_) δ 10.05 (s, 1H), 7.62–7.51 (m, 2H), 7.14–7.09 (m, 2H), 7.08 (s, 1H), 6.49 (s, 1H), 4.34 (dd, *J* = 5.6, 2.7 Hz, 2H), 4.24–4.18 (m, 2H), 3.83 (s, 3H). ^13^C NMR (101 MHz, DMSO-d_6_) δ 182.3, 159.7, 152.0, 148.2, 142.8, 139.0, 130.2 (2C), 124.3, 118.1, 114.8 (2C), 112.6, 112.3, 100.4, 88.3, 65.2, 63.5, 55.5. IR, v_max_/cm^−1^: 3344, 2914, 2211, 1685, 1642, 1586, 1470, 1310, 1242, 1170, 1138. HRMS (ES TOF) calculated for (M + Na)^+^ C_19_H_14_N_2_NaO_4_ 357.0846, found 357.0837 (2.6 ppm).

*(E)-2-(8-oxo-2H-[1,4]dioxino [2,3-f]indol-7(3H,6H,8H)-ylidene)-2-phenylacetonitrile* (**2cb**)

Red crystals, mp (EtOH) 290.3–291.7 °C, R*_f_* 0.23 (EtOAc/Hex, 1:2). Yield: 188 mg (0.62 mmol, 62%). ^1^H NMR (400 MHz, DMSO-d_6_) δ 10.15 (s, 1H), 7.62–7.53 (m, 4H), 7.46 (t, *J* = 7.1 Hz, 1H), 7.08 (s, 1H), 6.48 (s, 1H), 4.34 (dd, *J* = 5.6, 2.7 Hz, 2H), 4.22 (dd, *J* = 5.4, 2.7 Hz, 2H). ^13^C NMR (101 MHz, DMSO-d_6_) δ 182.4, 152.1, 148.2, 143.7, 139.1, 132.3, 129.36 (2C), 128.96, 128.73 (2C), 118.1, 112.47, 112.43, 100.5, 87.8, 65.2, 63.5. IR, v_max_/cm^−1^: 3300, 2975, 2218, 1699, 1608, 1490, 1333, 1208, 1164, 1067, 930, 896. HRMS (ES TOF) calculated for (M + Na)^+^ C_18_H_12_N_2_NaO_3_ 327.0735, found 327.0740 (1.5 ppm).

*(E)-2-(1-hydroxy-3-oxoindolin-2-ylidene)-2-phenylacetonitrile* (**10ab**): 

A 5-mL round bottom flask equipped with a magnetic stirring bar was charged with 2′-nitroacetophenone (1 mmol, 165 mg), benzaldehyde (1 mmol, 106 mg), KCN (2 mmol, 130 mg), H_2_O (130 mg), and MeOH (1 mL) and refluxed for 30 min (TLC control). The reaction mixture was cooled to room temperature, and after that, MeOH (2 mL) and phenacyl bromide (1 mmol, 199 mg) were added and the reaction mixture was allowed to stand overnight. The precipitate that had formed was filtered off and washed with methanol to obtain (*E*)-2-(1-hydroxy-3-oxoindolin-2-ylidene)-2-phenylacetonitrile as a yellowish solid, mp (MeOH) 197.3–198.6 °C (decomposition), R*_f_* 0.23 (EtOAc/Hex, 1:2). Yield: 123 mg (0.47 mmol, 47%). ^1^H NMR (400 MHz, DMSO-d_6_) δ 8.38 (d, *J* = 7.9 Hz, 2H), 7.27 (t, *J* = 7.7 Hz, 2H), 7.11 (t, *J* = 7.0 Hz, 2H), 6.99 (t, *J* = 7.3 Hz, 1H), 6.63 (d, *J* = 8.1 Hz, 1H), 6.39 (t, *J* = 7.2 Hz, 1H). ^13^C NMR (101 MHz, DMSO-d_6_) δ 197.0, 170.0, 155.9, 137.7, 136.4, 127.7 (2C), 127.4 (2C), 123.9, 123.7, 122.7, 121.8, 116.6, 116.5, 75.1. IR, v_max_/cm^−1^: 3308, 2219, 1708, 1600, 1465, 1445, 1389, 1328, 1208, 1141. HRMS (ES TOF) calculated for (M + Na)^+^ C_16_H_10_N_2_NaO_2_ 285.0634, found 285.0629 (1.9 ppm).

*4-Oxo-2,4-diphenylbutanenitrile* (**12**): 

A 5-mL round bottom flask equipped with a magnetic stirring bar was charged with acetophenone (1 mmol, 120 mg), benzaldehyde (1 mmol, 106 mg), KCN (2 mmol, 130 mg), H_2_O (130 mg), and MeOH (1 mL) and refluxed for 120 min (TLC control). The reaction mixture was cooled to room temperature, AcOH (1 mmol, 60 mg, 57 µL) was added, and reflux was continued to another 30 min. Then, the reaction mixture was diluted with 100 mL of EtOAc and washed twice with 30 mL of concentrated NaHCO_3_ solution. The organic layer was concentrated and purified by column chromatography (eluent EtOAc:Hex 1:4). Then, 4-Oxo-2,4-diphenylbutanenitrile was obtained as a white solid. NMR spectra were in agreement to previously published [26]. Yield: 35 mg (0.15 mmol, 15%). ^1^H NMR (400 MHz, Chloroform-d) δ 7.93 (dt, *J* = 7.1, 1.4 Hz, 2H), 7.64–7.55 (m, 1H), 7.52–7.30 (m, 7H), 4.56 (dd, *J* = 8.1, 6.0 Hz, 1H), 3.73 (dd, *J* = 18.0, 8.0 Hz, 1H), 3.51 (dd, *J* = 18.0, 5.9 Hz, 1H). ^13^C NMR (101 MHz, CDCl_3_) δ 194.7, 135.7, 135.3, 134.0, 129.4 (2C), 128.9 (2C), 128.5, 128.2 (2C), 127.6 (2C), 120.8, 44.6, 32.0. HRMS (ES TOF) calculated for (M + Na)^+^ C_16_H_13_NNaO 258.0889, found 258.0888 (0.6 ppm).

## Figures and Tables

**Table 1:**
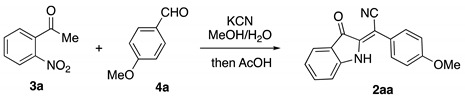
Optimization of the reaction conditions for one-pot conversion of 2-nitroacetophenone (**3a**) and *p*-anisaldehyde (**4a**) into (*E*)-2-(3-oxoindolin-2-ylidene)-2-arylacetonitriles (**2aa**).

	KCN *^a^*	Temperature (°C)/Time (h)	Methanol/Water/AcOH *^b^*	Yield of 2aa, % *^c^*
1	130	65/1	2000/130/150	64
2	260	65/0.5	2000/260/300	65
**3**	**130**	**65/0.5**	**1000/130/150**	**95(87) ** *^d^*
4	130	20/12	1000/130/150	58
5	130	65/0.5	1000/0/150	79
6	130	65/0.5	1000/130/195 *^e^*	38
7	130	65/0.5	1000/130/92 *^f^*	49

*^a^* Amount of KCN in mg per 1 mmol of starting material **3a** is listed. *^b^* Volumes of methanol, water, and acetic acid in μL per 1 mmol of starting material **3a** are listed. *^c^* NMR yields are reported. The best result is shown in bold. *^d^* Isolated yield of purified product **2aa** is provided in parentheses. *^e^* H_3_PO_4_ was used instead of AcOH. *^f^* HCOOH was used instead of AcOH.

## Data Availability

Supporting Information data include NMR spectral charts.

## Supplementary Materials

The following are available online at https://www.mdpi.com/article/10.3390/molecules27092808/s1, ^1^H and ^13^C NMR spectral charts (Figures S1–S52), HRMS spectral charts (Figures S53–S78), and X-Ray crystallography data (Figure S79, Tables S1–S7). References [27,28,29] are cited in the supplementary materials.

## Sample Availability

Not available.
