# Supplementary material for: One-Pot Synthesis of (E)-2-(3-Oxoindolin-2-ylidene)-2-arylacetonitriles
Source: Molecules. 2022 Apr 28;27(9):2808. doi: 10.3390/molecules27092808 (PMC9099830; doi:10.3390/molecules27092808)
Supplement: Supplementary file 1 [file molecules-27-02808-s001.zip › molecules-1652786-supplementary.pdf]

Electronic Supplementary Material (ESI)

One-pot synthesis of (E)-2-(3-oxoindolin-2-ylidene)-2-arylacetonitriles

Nicolai A. Aksenov[1,*], Alexander V. Aksenov[1], Igor A. Kurenkov[1], Nikita K. Kirillov[1], Dmitrii A. Aksenov[1], Nikolai A. Arutiunov[1], Daria S. Aksenova[1], and Michael Rubin[1,2,*]

[1] Department of Chemistry, North Caucasus Federal University, 1a Pushkin St., 355017 Stavropol, Russia; aaksenov@ncfu.ru (A.V.A.); kurenkman@icloud.com (I.A.K.); lyncheron@gmail.com (N.K.K.); daksenov@ncfu.ru (D.A.A.); naarutiunov@ncfu.ru (N.A.A.); dasha.severilo@gmail.com (D.S.A.)
[2] Department of Chemistry, University of Kansas, 1567 Irving Hill Road, Lawrence, KS 66045, USA;
* Correspondence: naksenov@ncfu.ru (N.A.A.); mrubin@ku.edu (M.R.)

Supporting Information

NMR Spectral Charts……………………………………………………………………………………………. S2
^{1}H and ^{13}C NMR spectral charts for (E)-2-aryl-2-(3-oxoindolin-2-ylidene)acetonitriles (2aa-2cb)…………………………… S2
^{1}H and ^{13}C NMR spectral charts for(*E*)-2-(1-hydroxy-3-oxoindolin-2-ylidene)-2-phenylacetonitrile (10ab):………………… S48
^{1}H and ^{13}C NMR spectral charts for 4-oxo-2,4-diphenylbutanenitrile (12)…………………………………………………….. S50
^{1}H and ^{13}C NMR spectral charts for 5*b*-hydroxy-5*b*,10-dihydrobenzo[4',5']thieno[2',3':3,4]cyclopenta[1,2-*b*]indole-11-carbonitrile (2ap):……… S52
HRMS spectral charts…………………………………………………………………………………………………… S54
HRMS spectral charts for (E)-2-aryl-2-(3-oxoindolin-2-ylidene)acetonitriles (2aa-2cb)………………………………………. S54
HRMS spectral charts for (*E*)-2-(1-hydroxy-3-oxoindolin-2-ylidene)-2-phenylacetonitrile (10ab):…………………………… S62
HRMS spectral charts for 4-oxo-2,4-diphenylbutanenitrile (12).……………………………………………………………….. S63
HRMS spectral charts for 5*b*-hydroxy-5*b*,10-dihydrobenzo[4',5']thieno[2',3':3,4]cyclopenta[1,2-*b*]indole-11-carbonitrile (2ap):…….. S64
X-Ray crystallography data………………………………………………………………………………………… S65
References…… S75

^{1}H and ^{13}C NMR spectral charts for (E)-2-aryl-2-(3-oxoindolin-2-ylidene)acetonitriles (2aa-2cb)

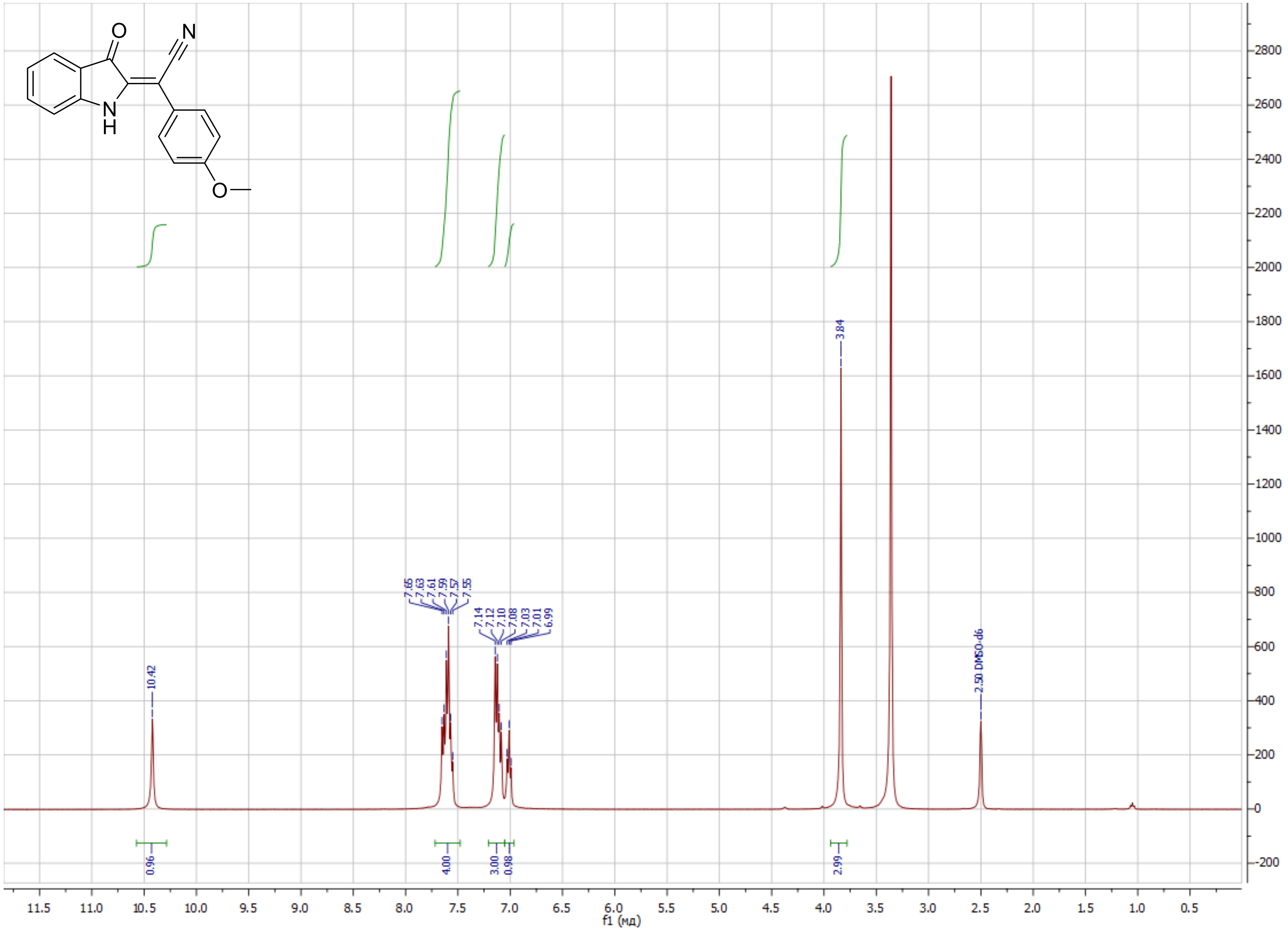

Figure S1. ^{1}H NMR spectrum of **2aa** in DMSO-d_6 (400 MHz)

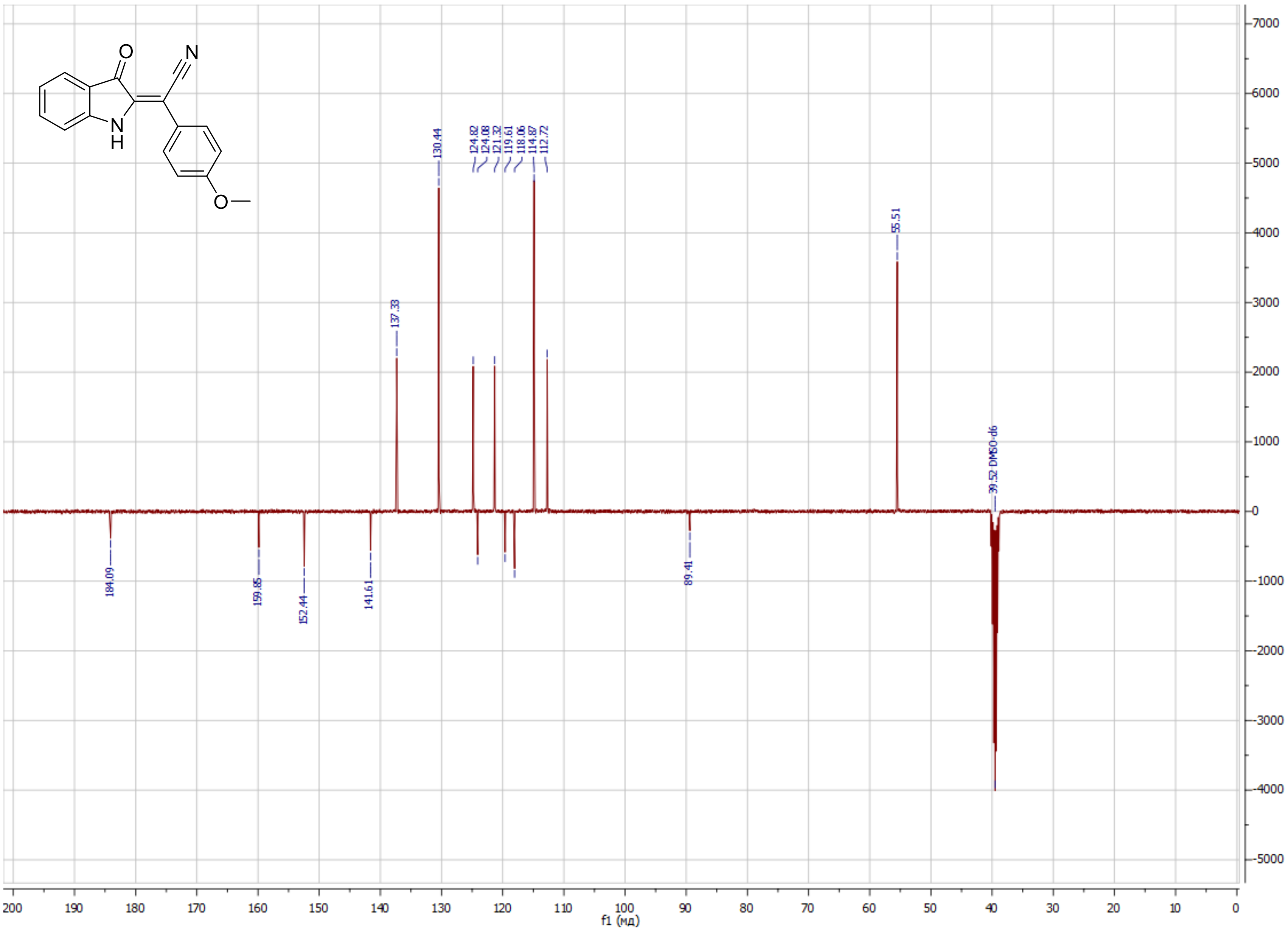

Figure S2. ^{13}C DEPTQ NMR spectrum of **2aa** in DMSO-d_6 (100 MHz)

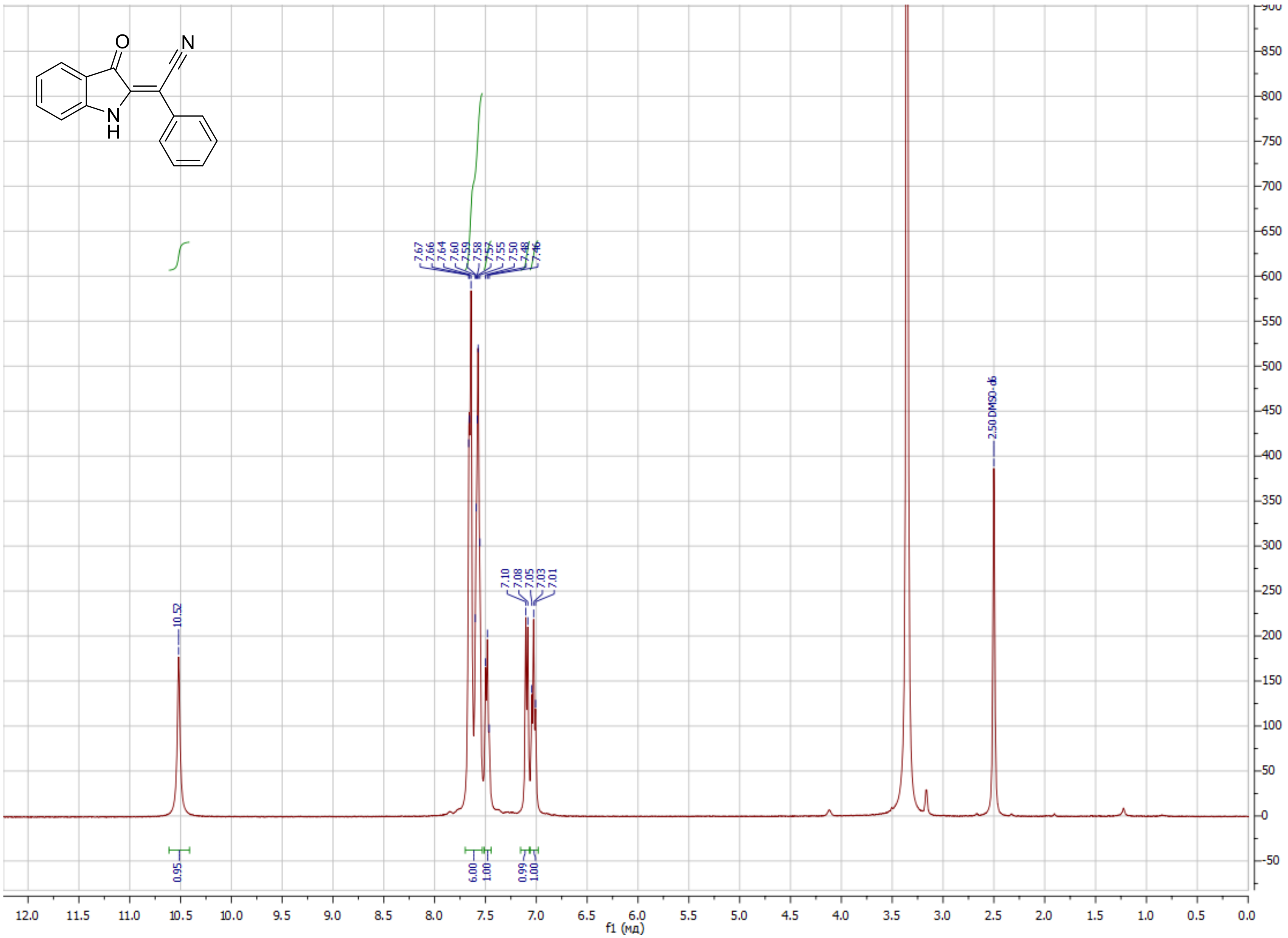

Figure S3. ^{1}H NMR spectrum of **2ab** in DMSO-d_6 (400 MHz)

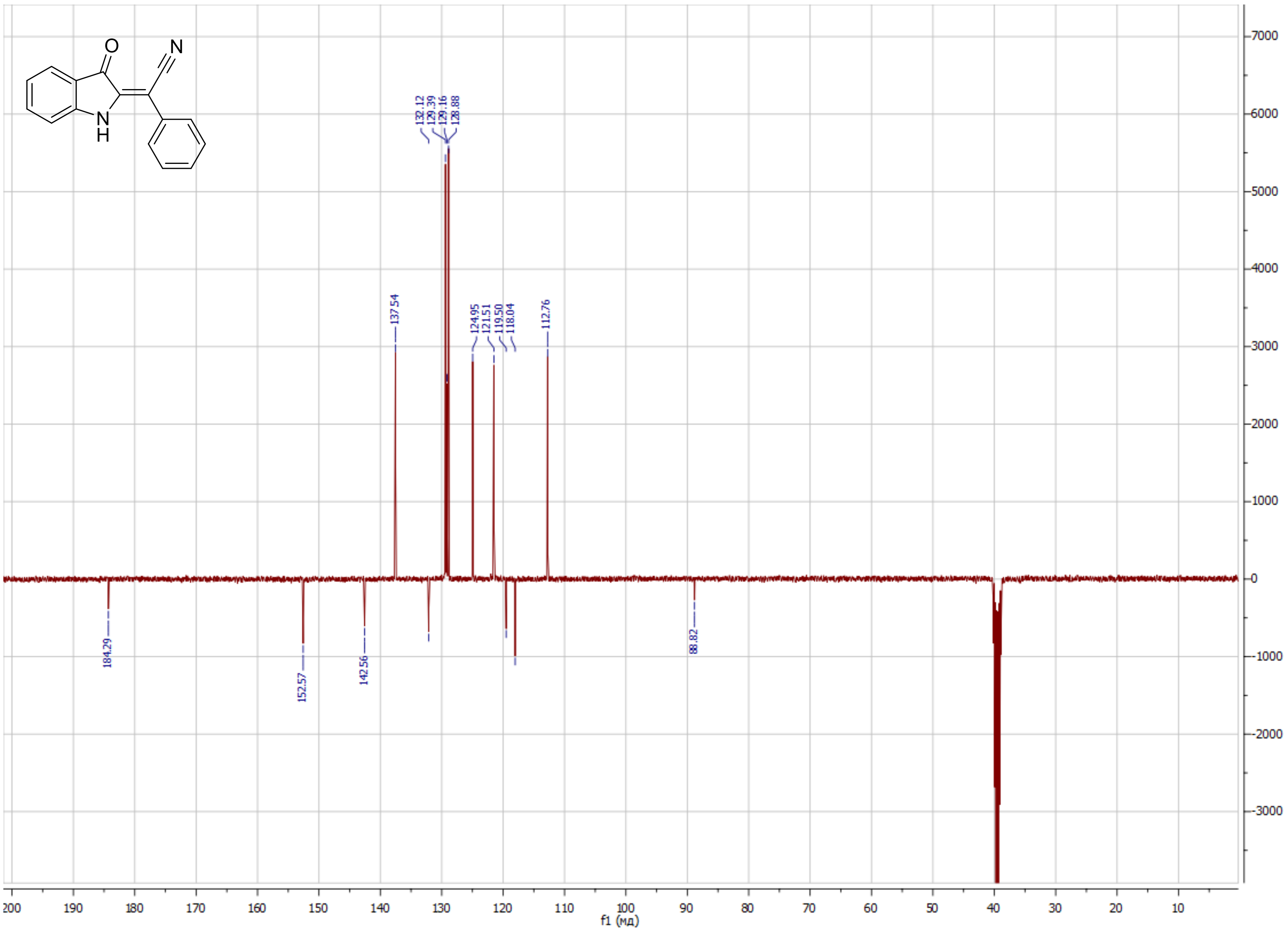

Figure S4. ^{13}C DEPTQ NMR spectrum of **2ab** in DMSO-d_6 (100 MHz)

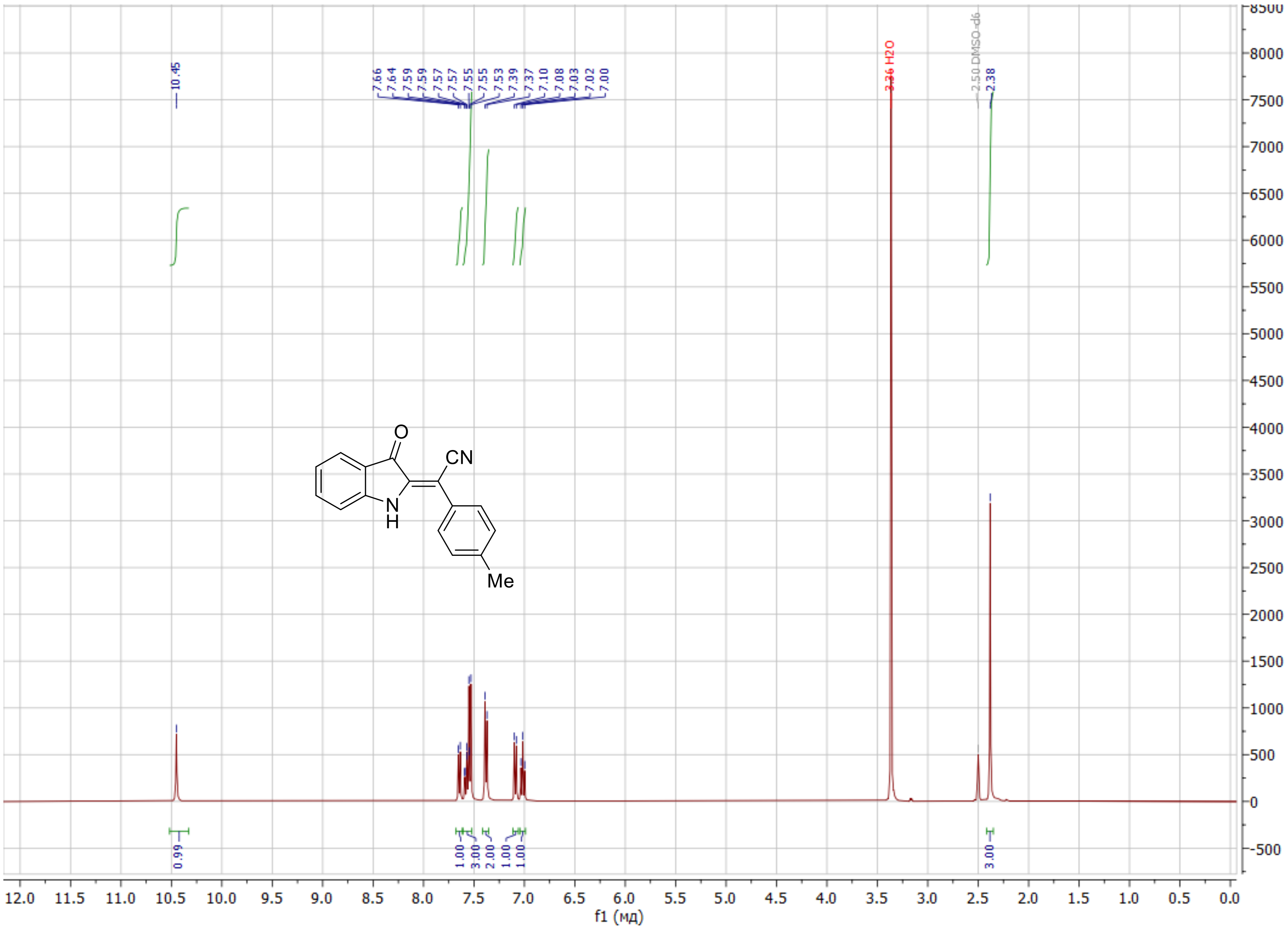

Figure S5. ^{1}H NMR spectrum of **2ac** in DMSO-d_6 (400 MHz)

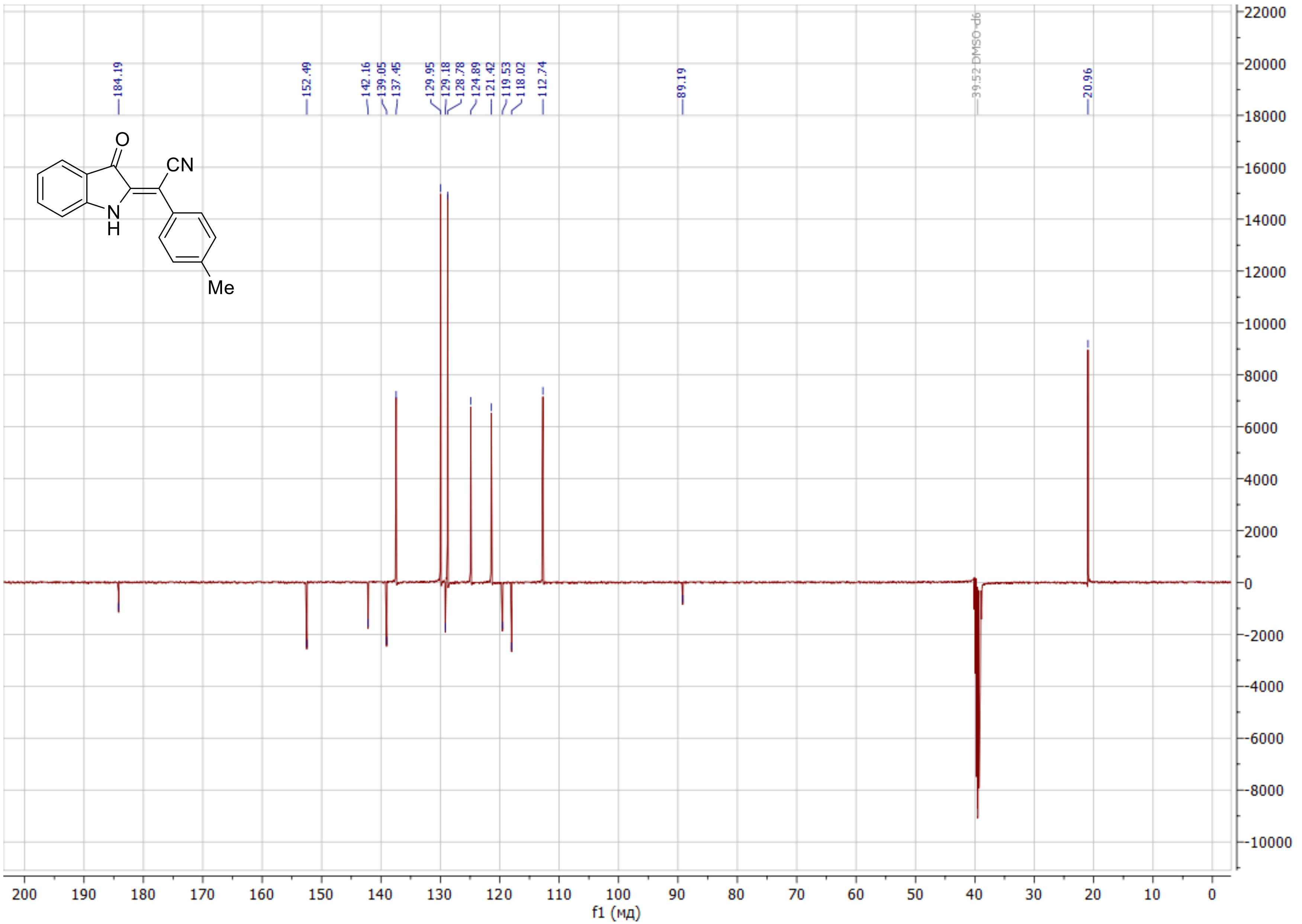

Figure S6. ^{13}C DEPTQ NMR spectrum of **2ac** in DMSO-d_6 (100 MHz)

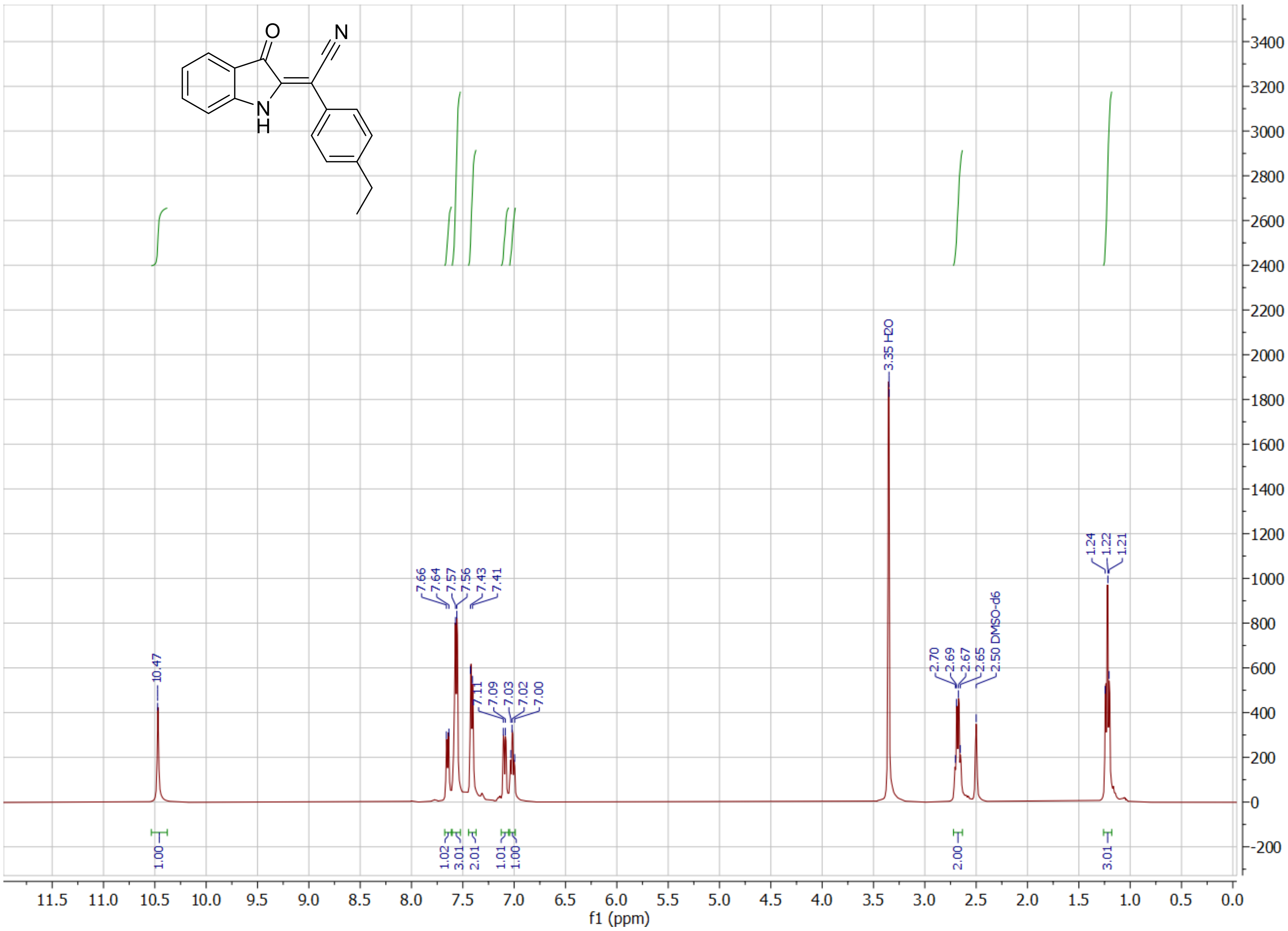

Figure S7. ^{1}H NMR spectrum of **2ad** in DMSO-d_6 (400 MHz)

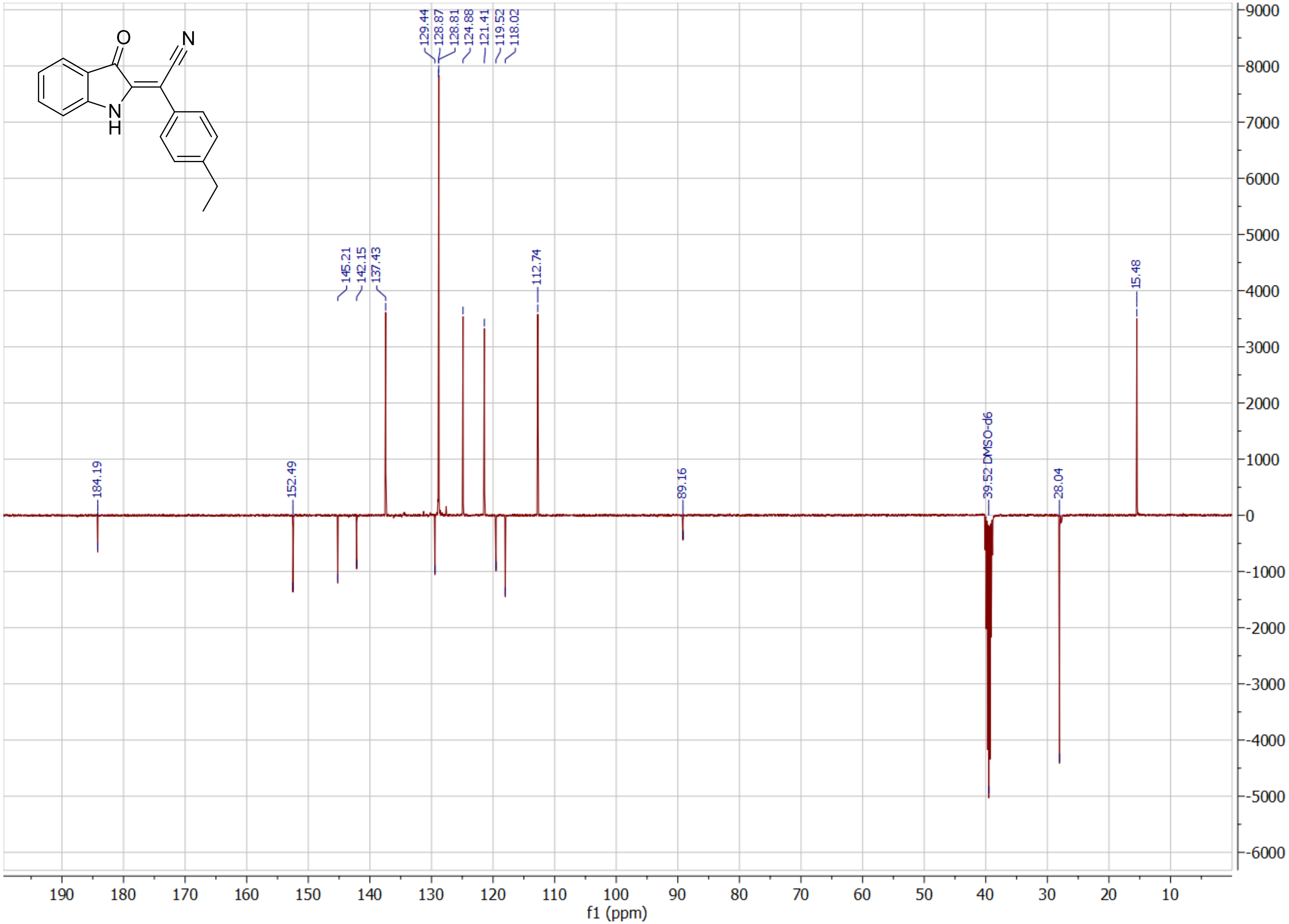

Figure S8. ^{13}C DEPTQ NMR spectrum of **2ad** in DMSO-d_6 (100 MHz)

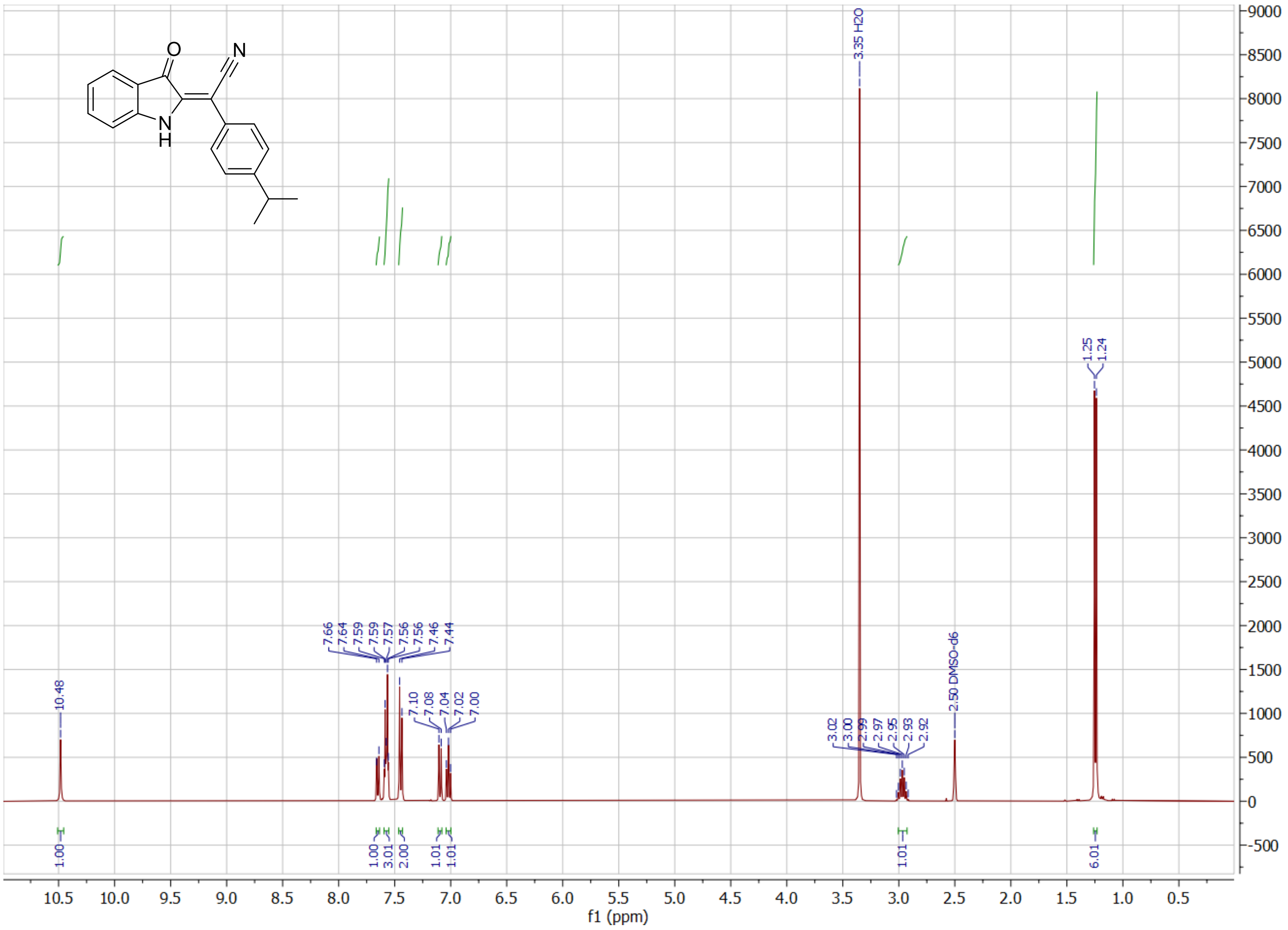

Figure S9. ^{1}H NMR spectrum of **2ae** in DMSO-d_6 (400 MHz)

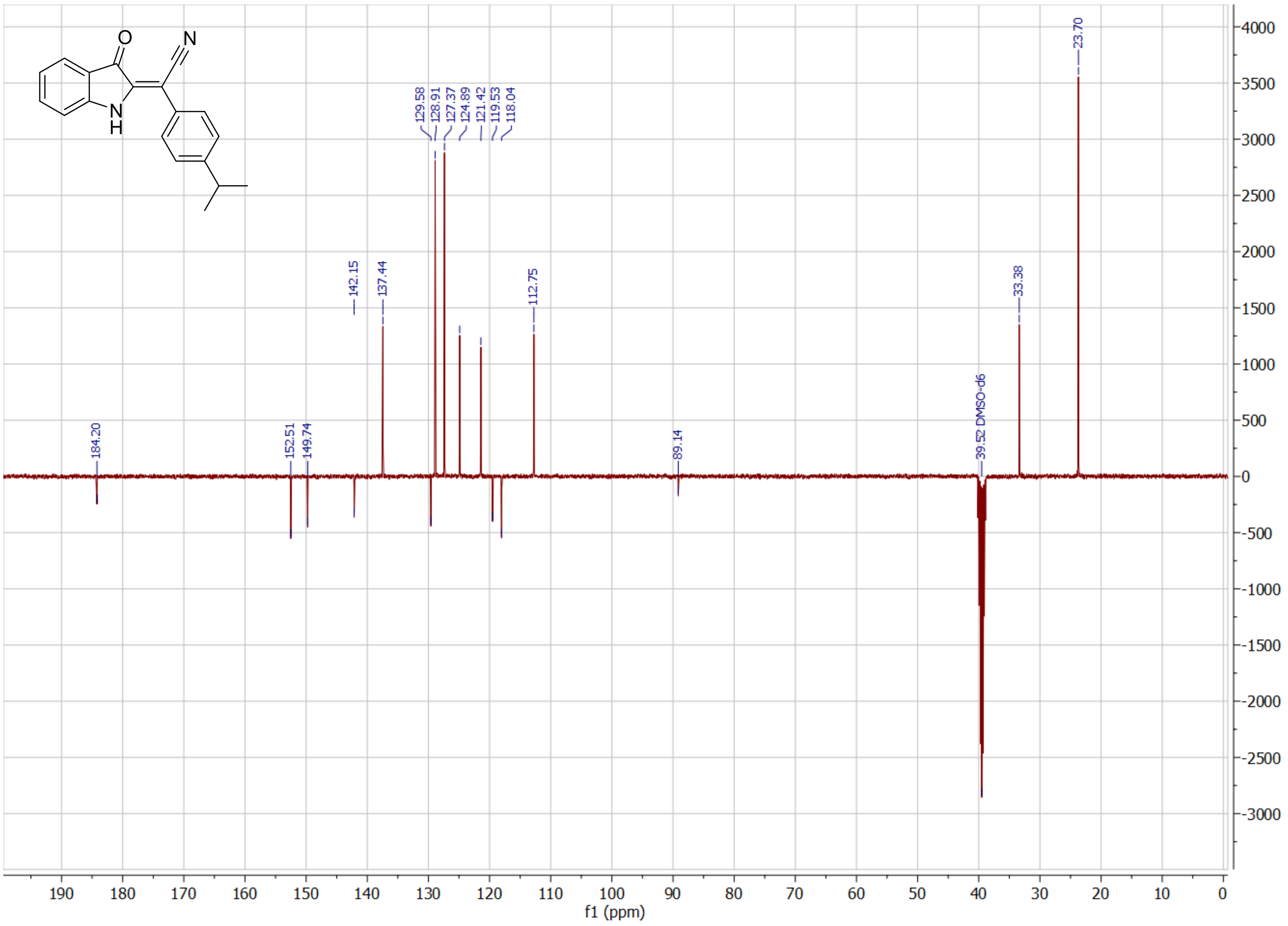

Figure S10. ^{13}C DEPTQ NMR spectrum of **2ae** in DMSO-d_6 (100 MHz)

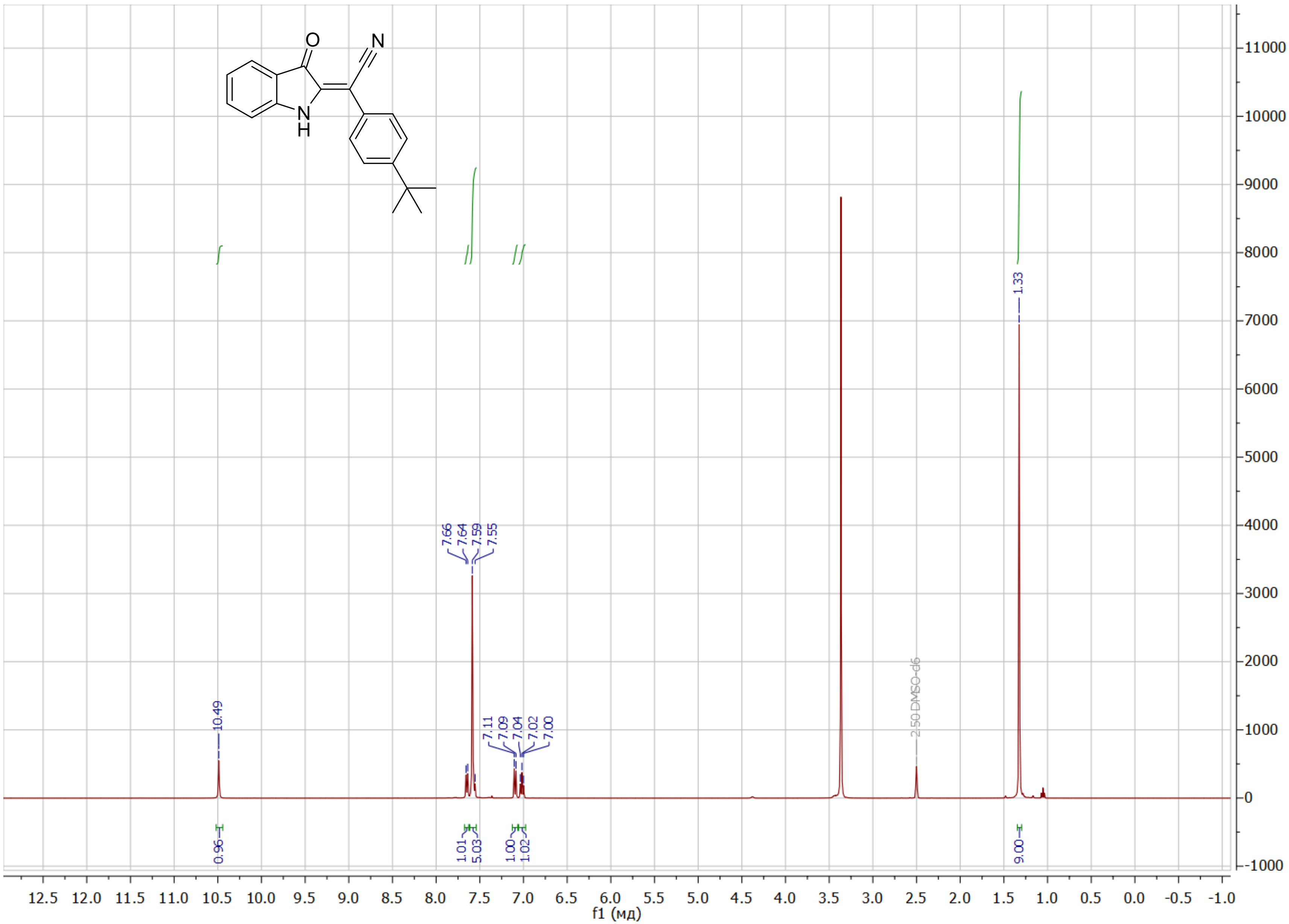

Figure S11. ^{1}H NMR spectrum of **2af** in DMSO-d_6 (400 MHz)

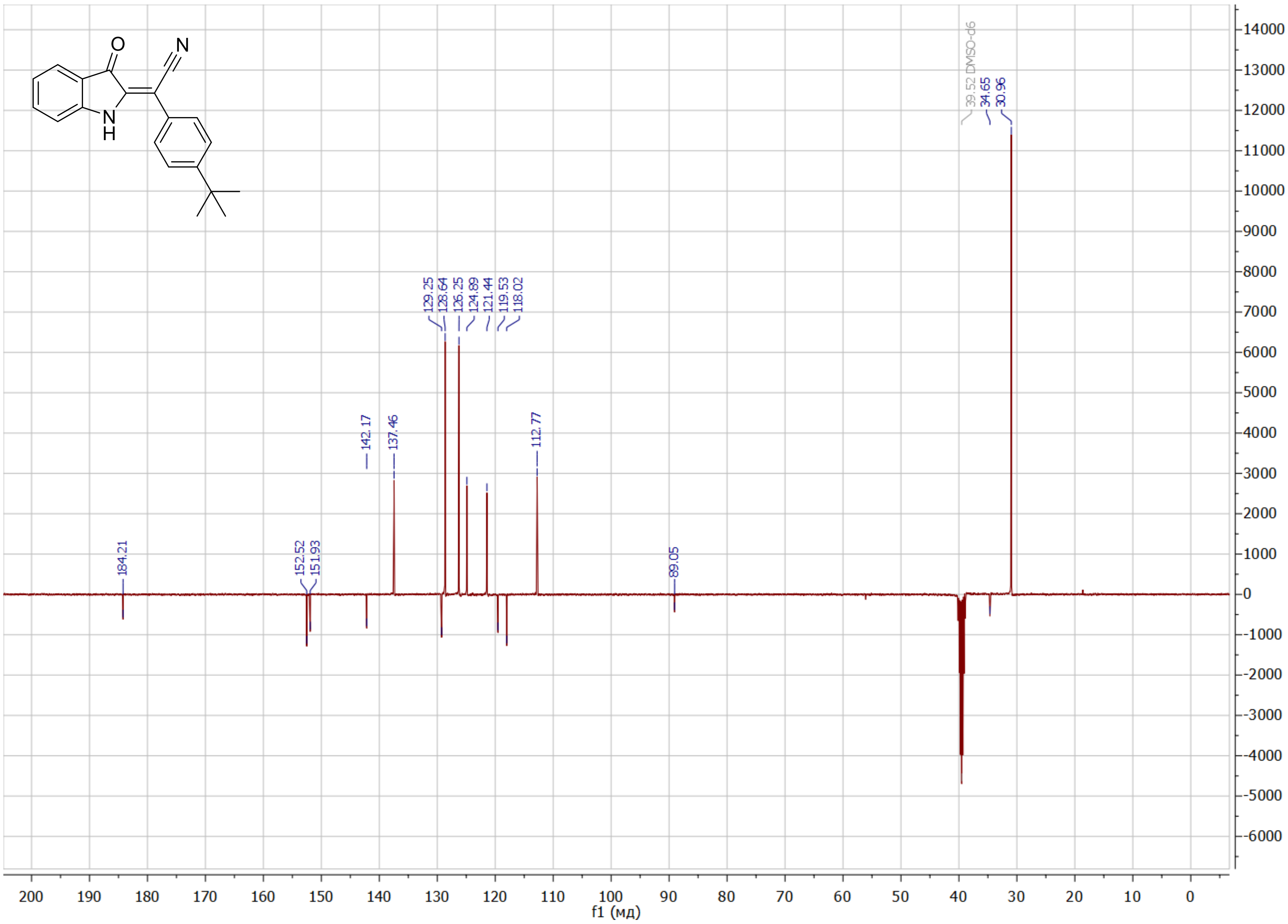

Figure S12. ^{13}C DEPTQ NMR spectrum of **2af** in DMSO-d_6 (100 MHz)

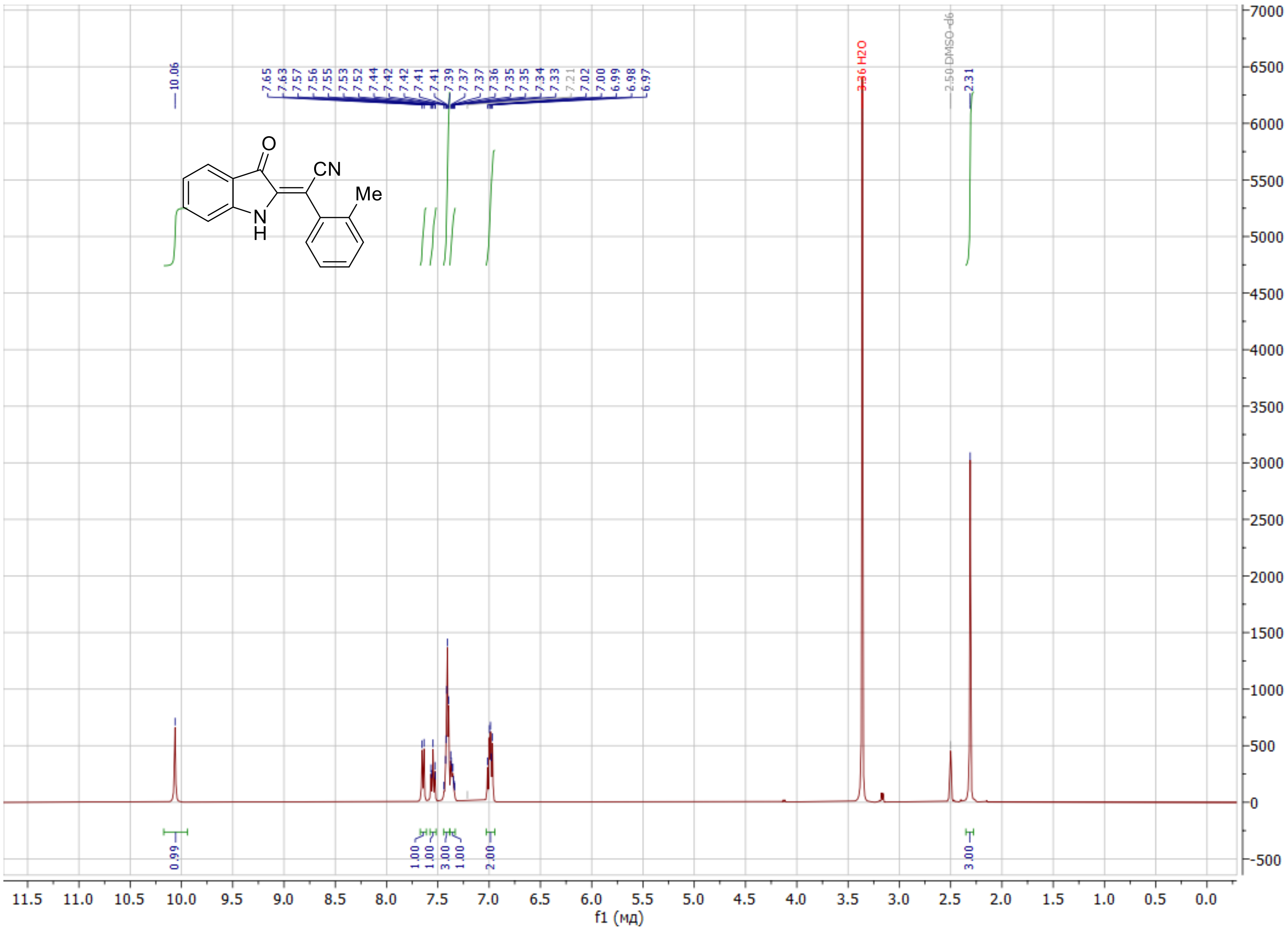

Figure S13. ^{1}H NMR spectrum of **2ag** in DMSO-d_6 (400 MHz)

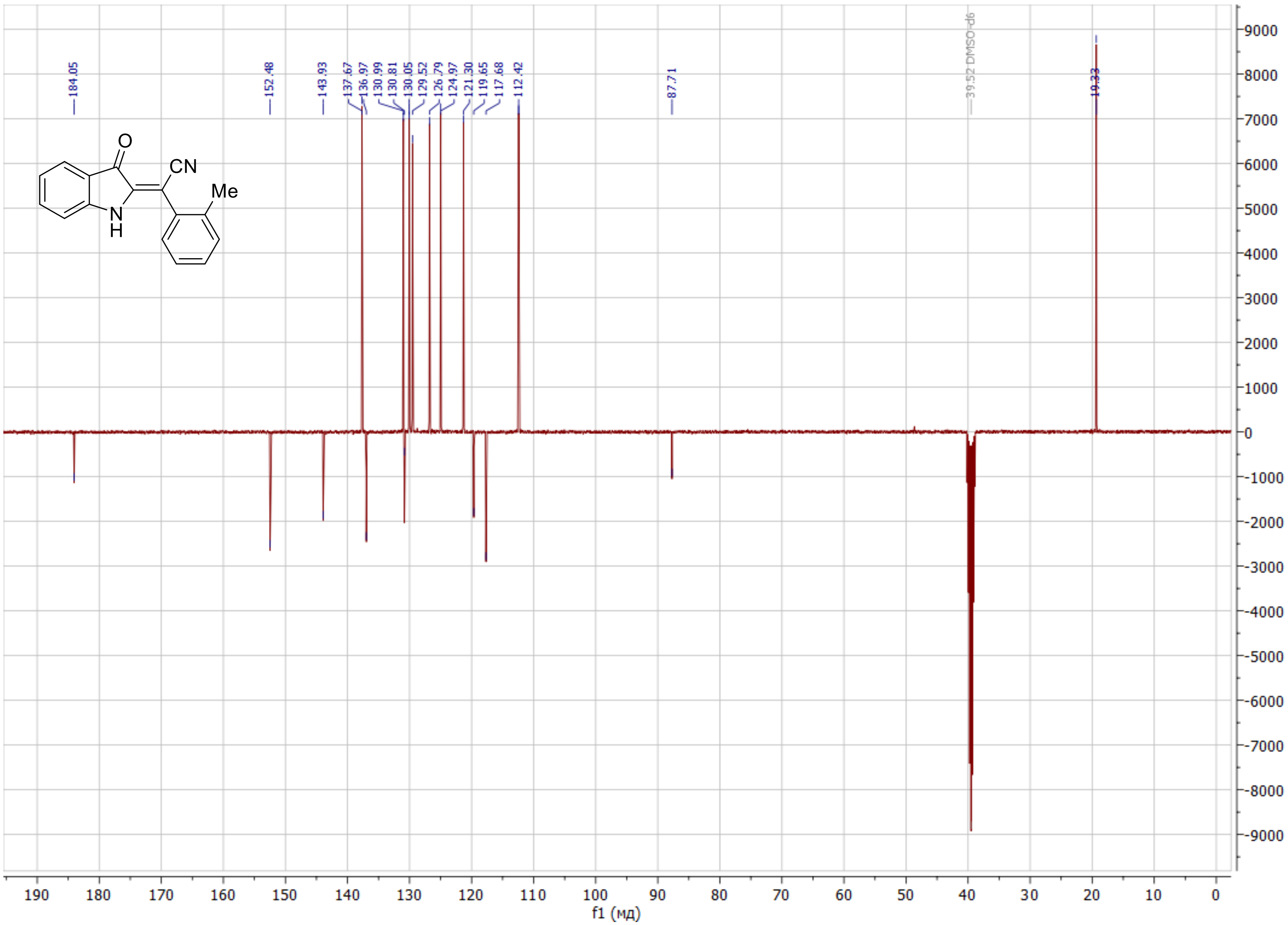

Figure S14. ^{13}C DEPTQ NMR spectrum of **2ag** in DMSO-d_6 (100 MHz)

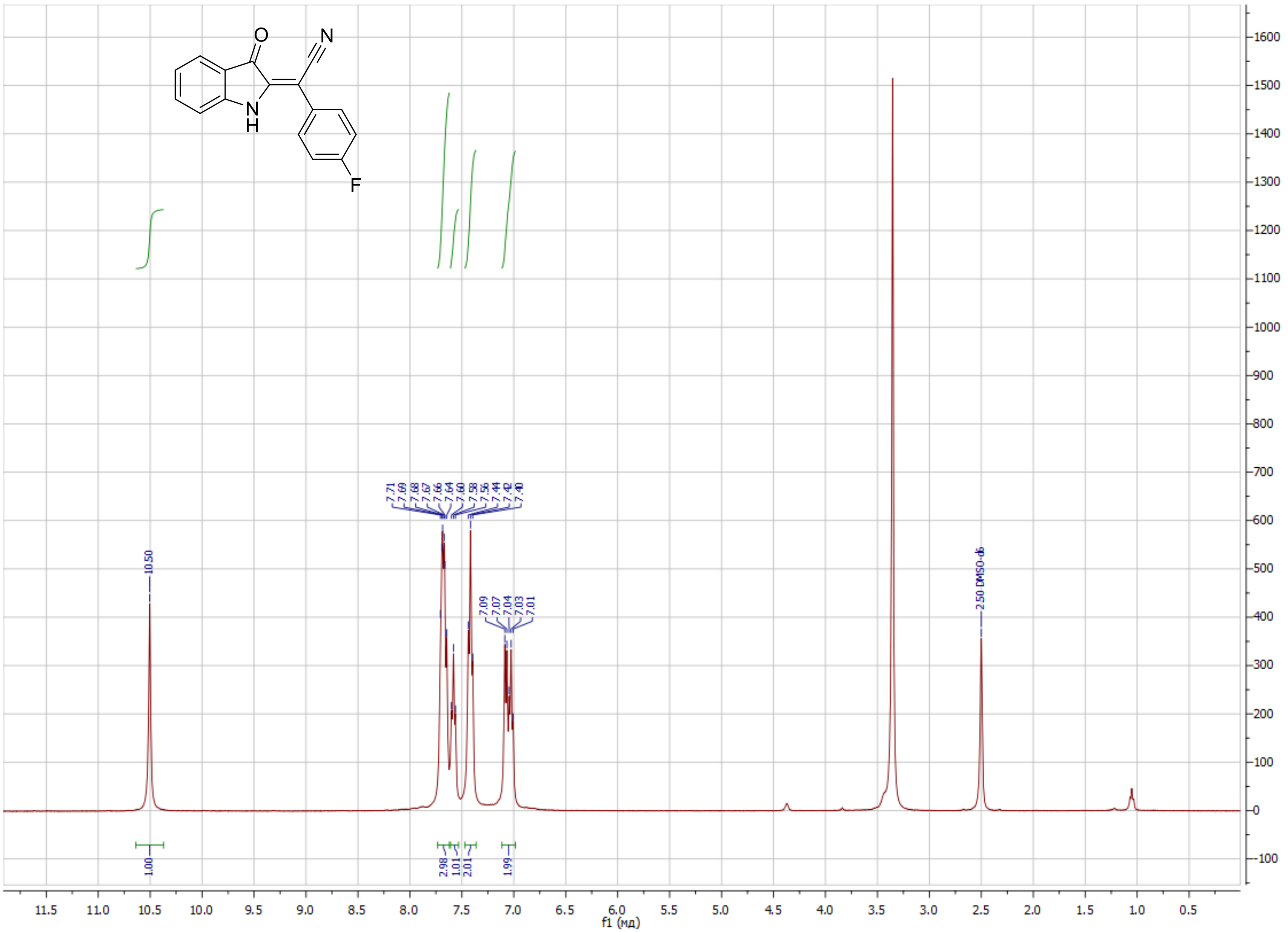

Figure S15. ^{1}H NMR spectrum of **2ah** in DMSO-d_6 (400 MHz)

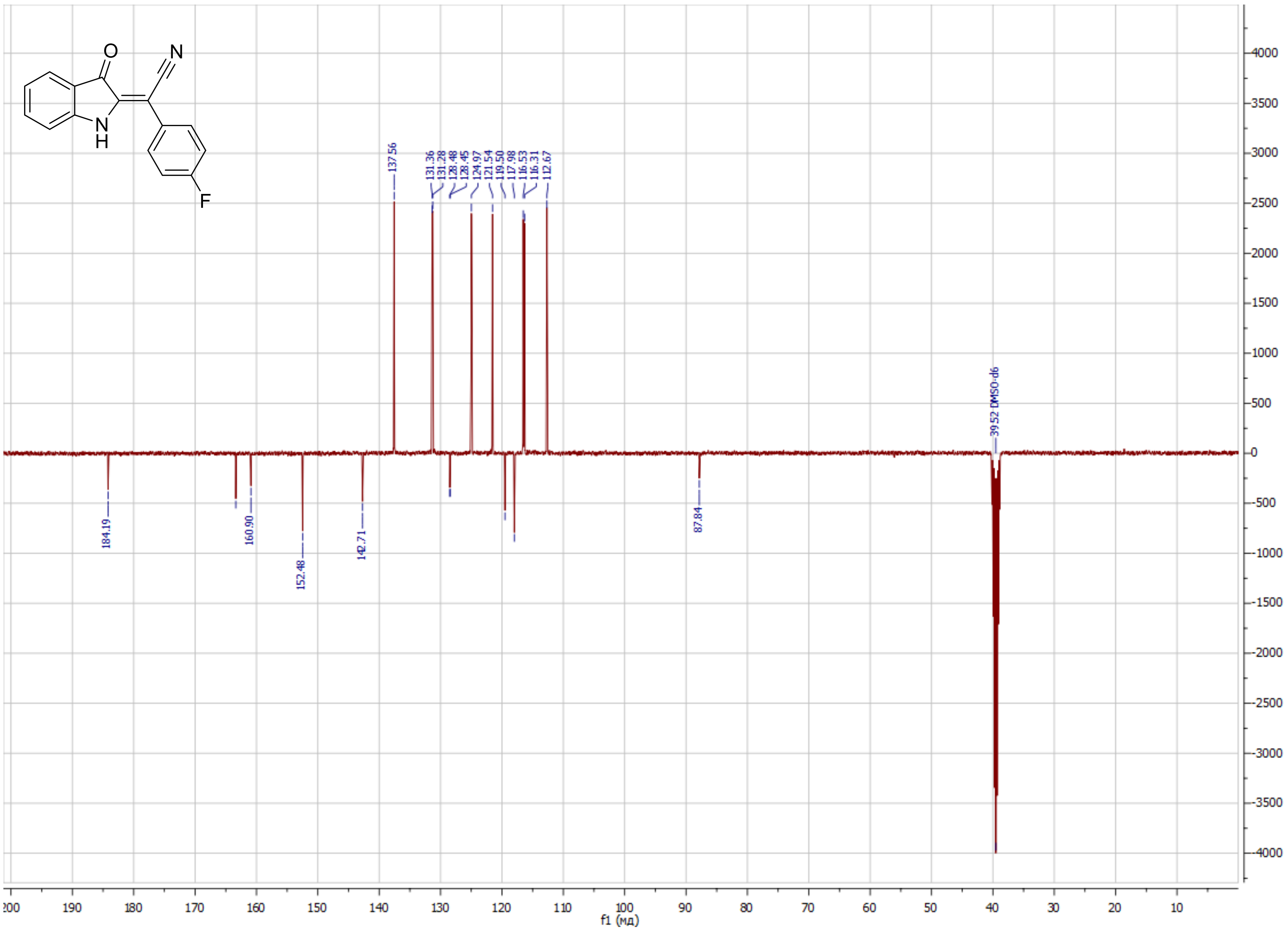

Figure S16. ^{13}C DEPTQ NMR spectrum of **2ah** in DMSO-d_6 (100 MHz)

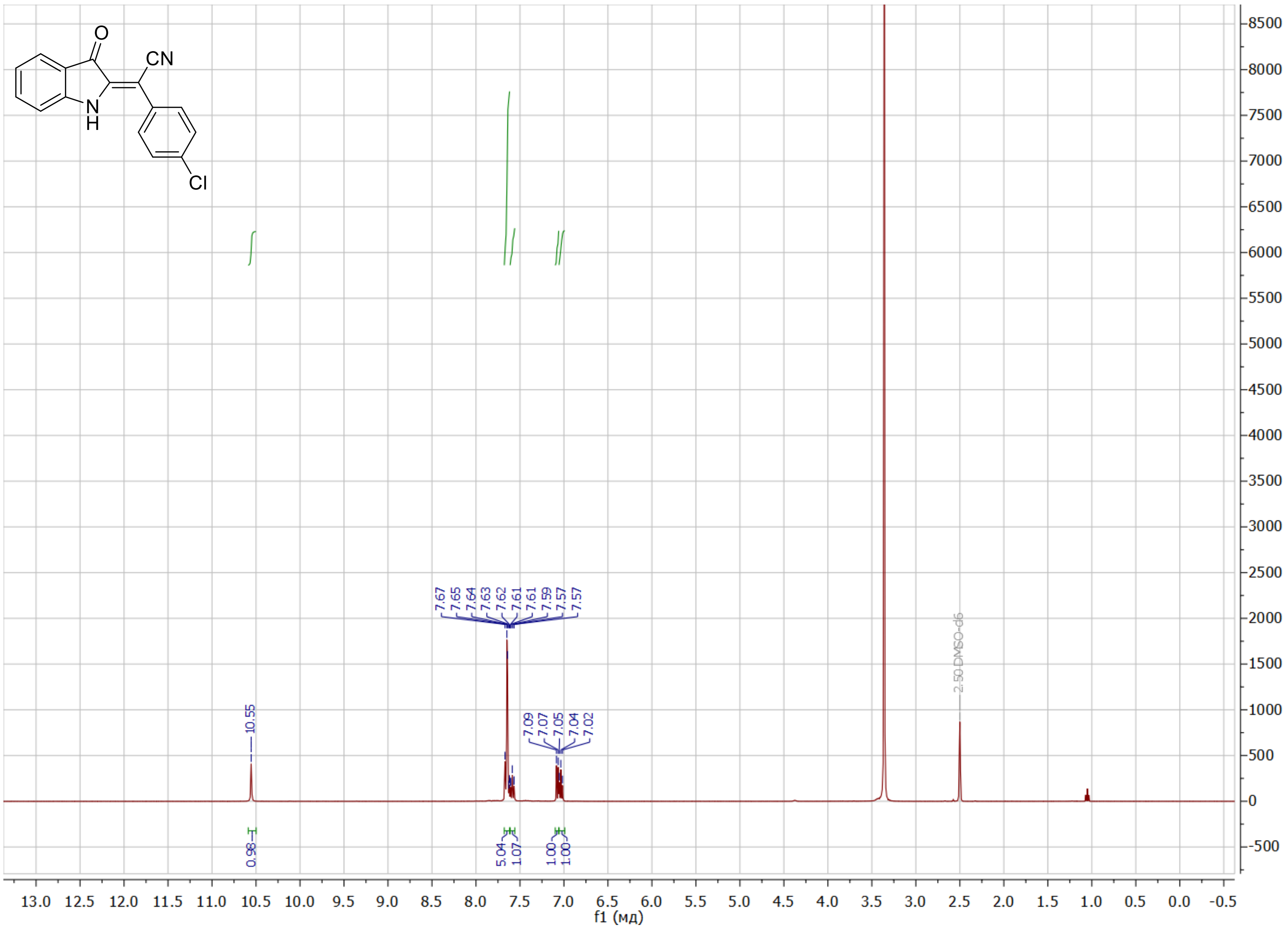

Figure S17. ^{1}H NMR spectrum of **2ai** in DMSO-d_6 (400 MHz)

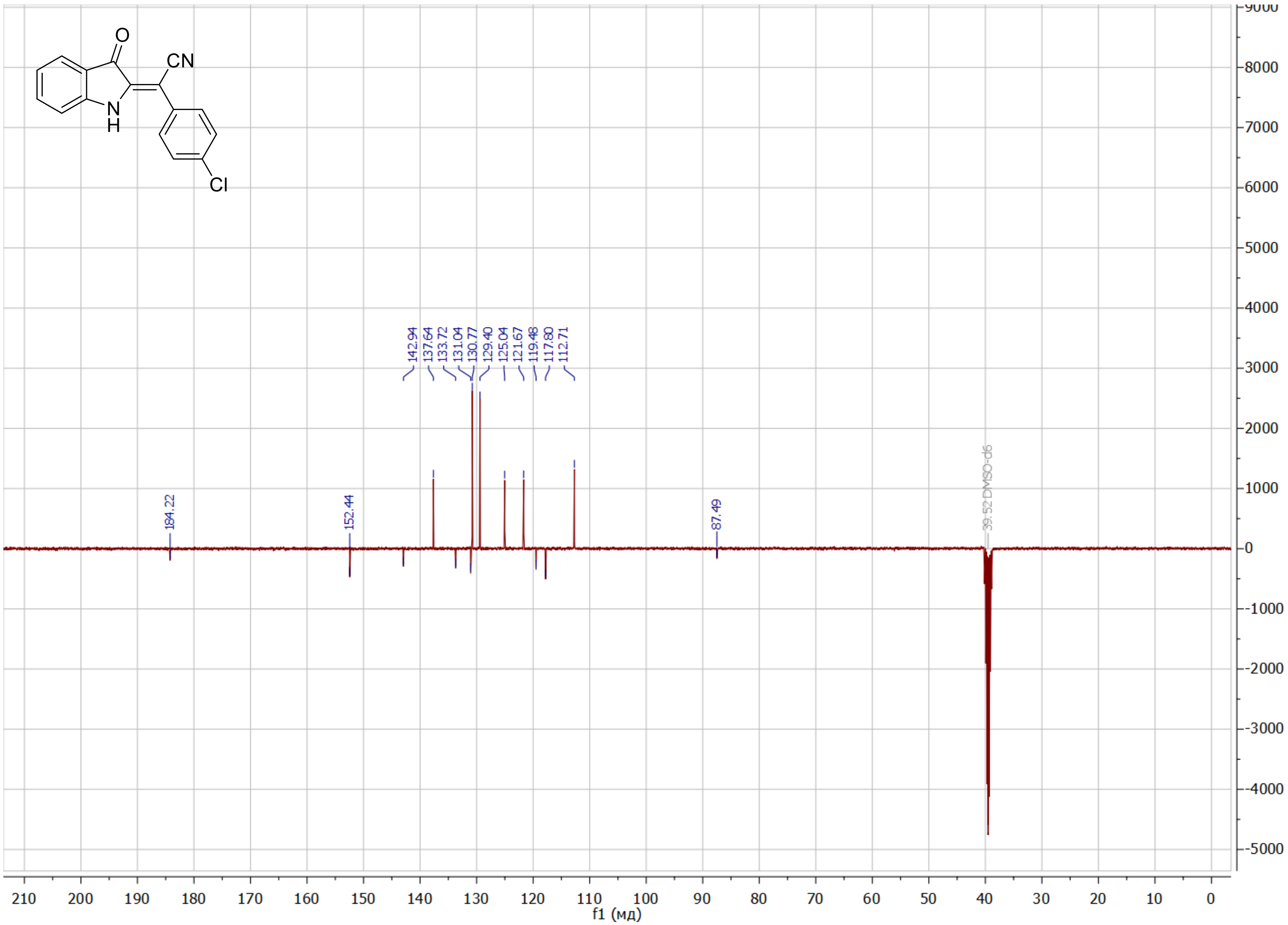

Figure S18. ^{13}C DEPTQ NMR spectrum of **2ai** in DMSO-d_6 (100 MHz)

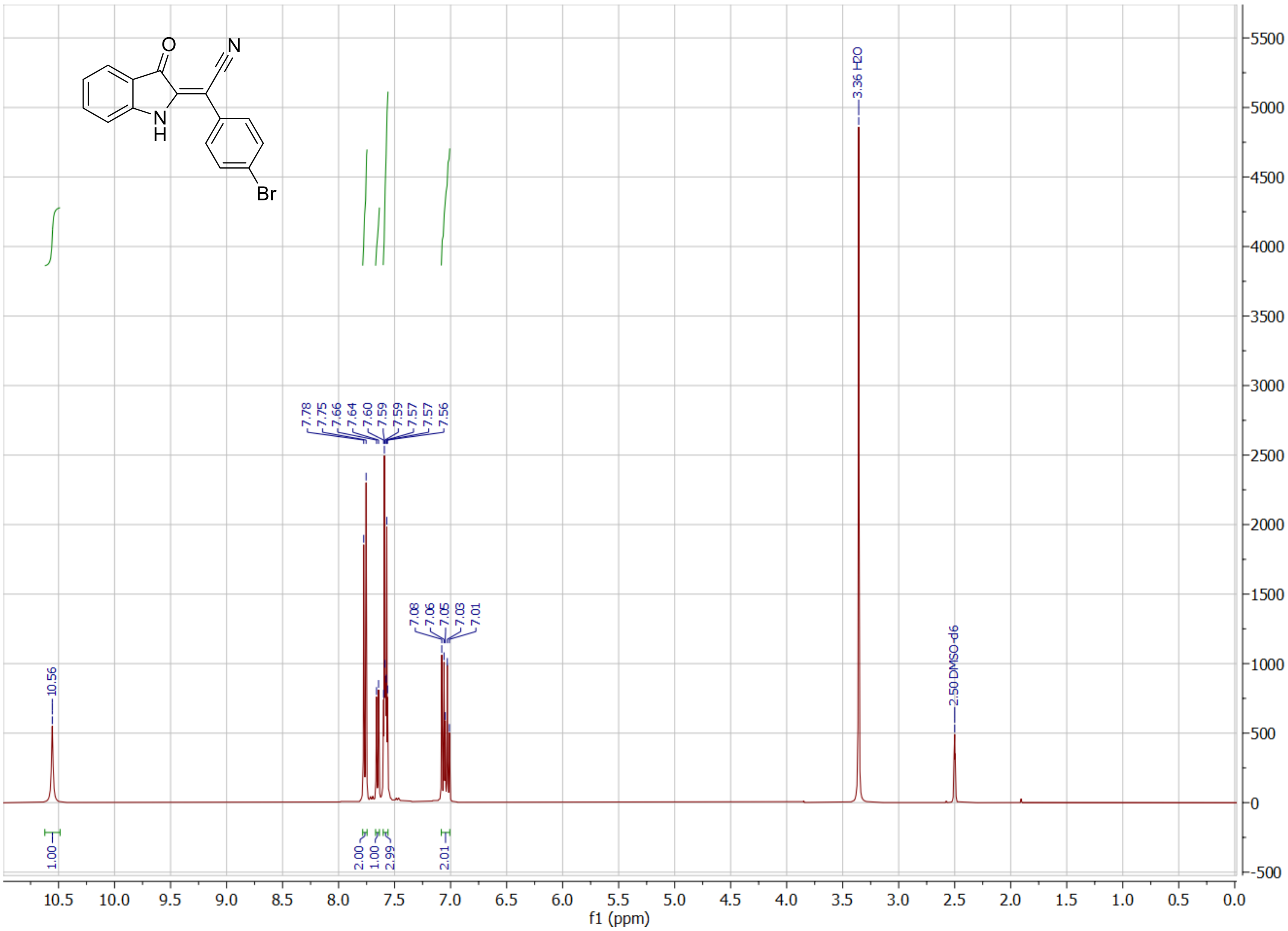

Figure S19. ^{1}H NMR spectrum of **2aj** in DMSO-d_6 (400 MHz)

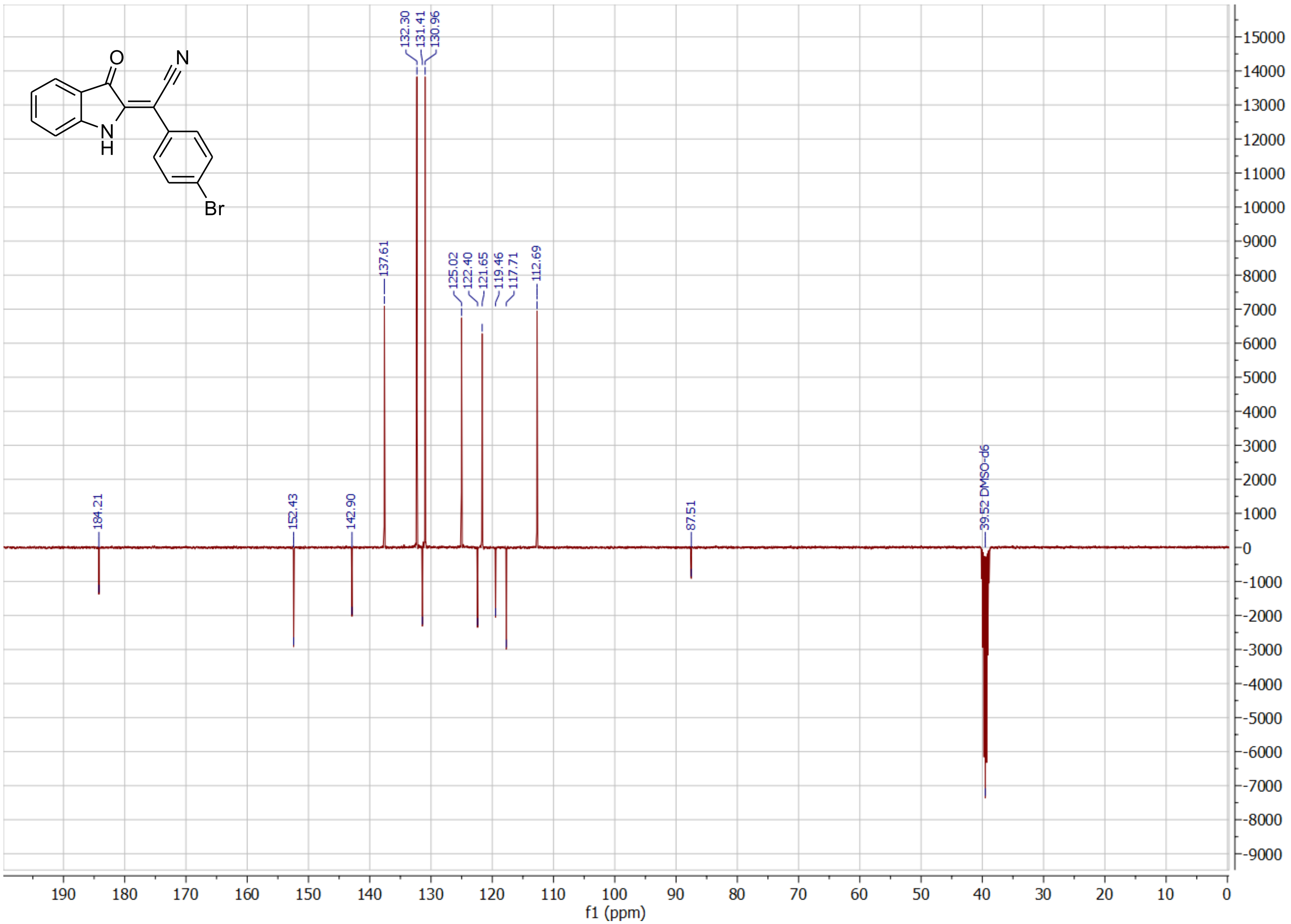

Figure S20. ^{13}C DEPTQ NMR spectrum of **2aj** in DMSO-d_6 (100 MHz)

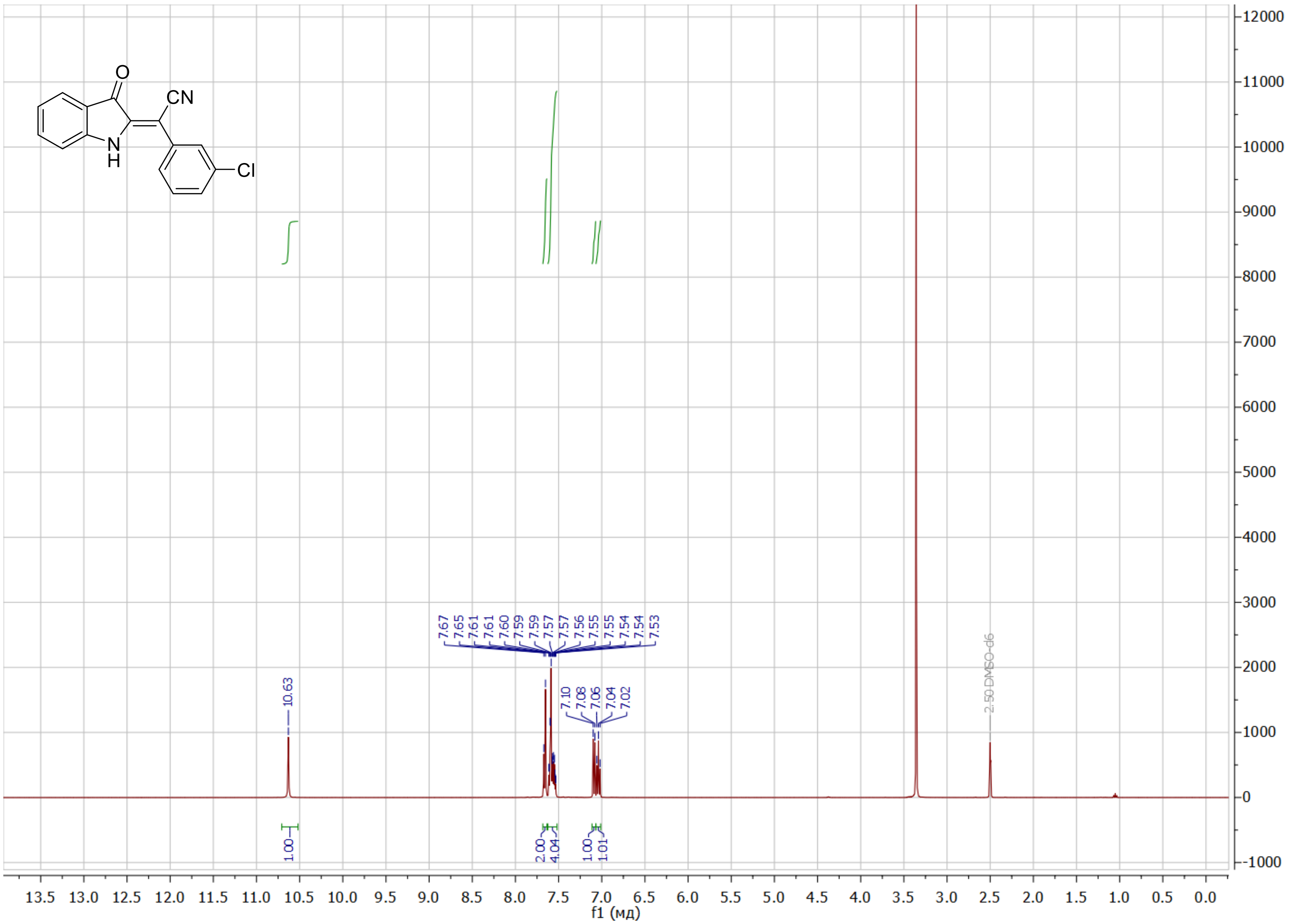

Figure S21. ^{1}H NMR spectrum of **2ak** in DMSO-d_6 (400 MHz)

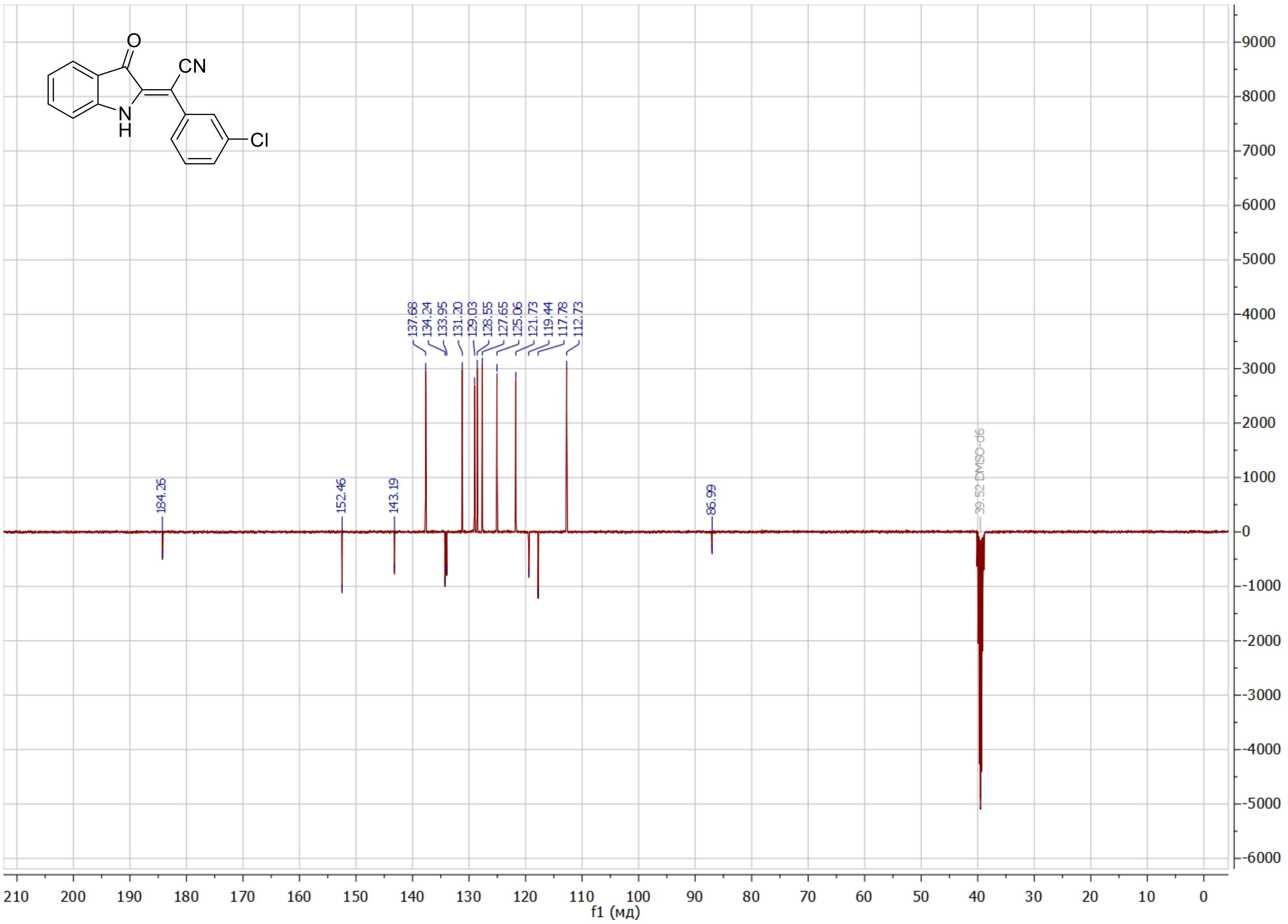

Figure S22. ^{13}C DEPTQ NMR spectrum of **2ak** in DMSO-d_6 (100 MHz)

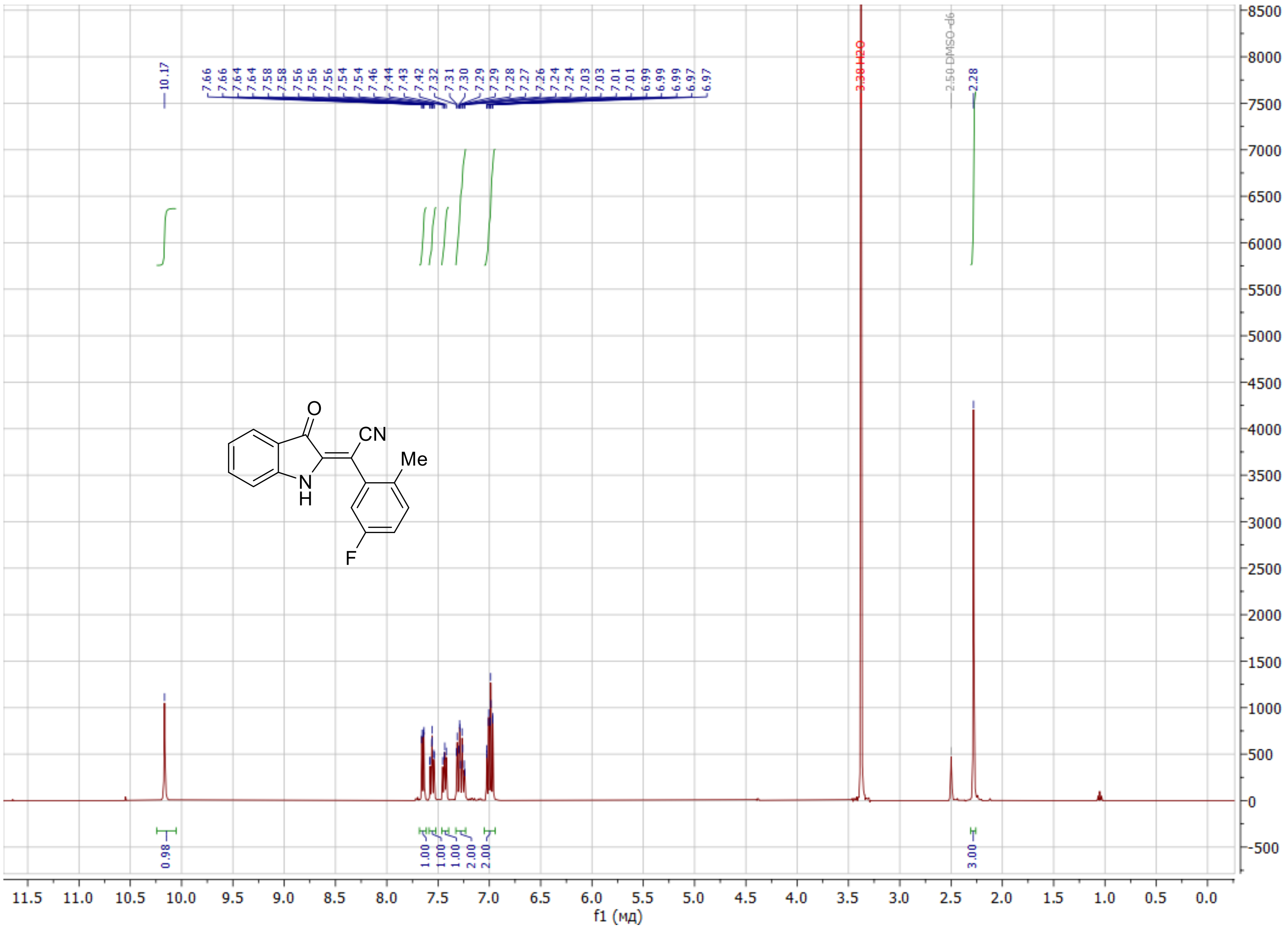

Figure S23. ^{1}H NMR spectrum of **2al** in DMSO-d_6 (400 MHz)

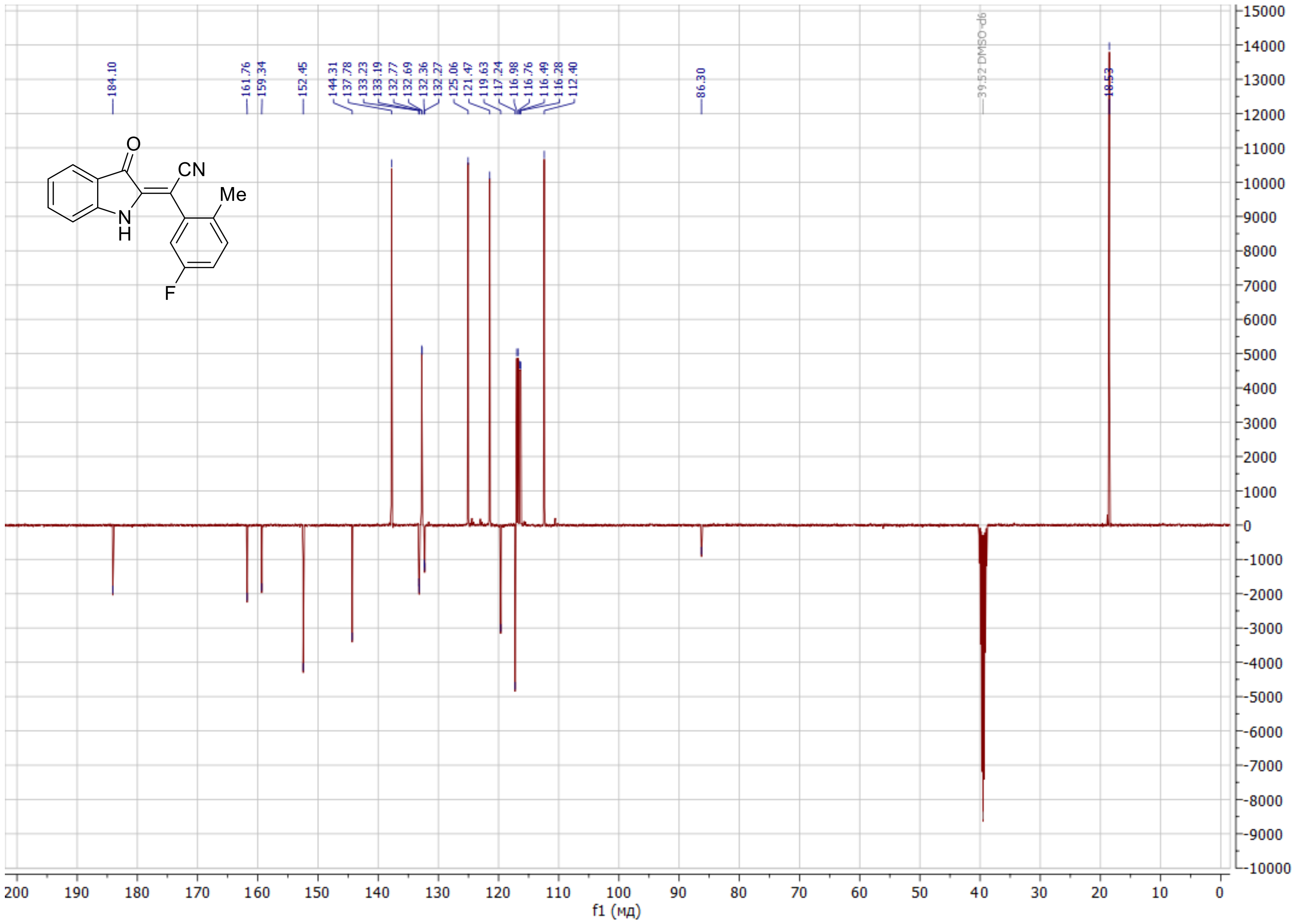

Figure S24. ^{13}C DEPTQ NMR spectrum of **2al** in DMSO-d_6 (100 MHz)

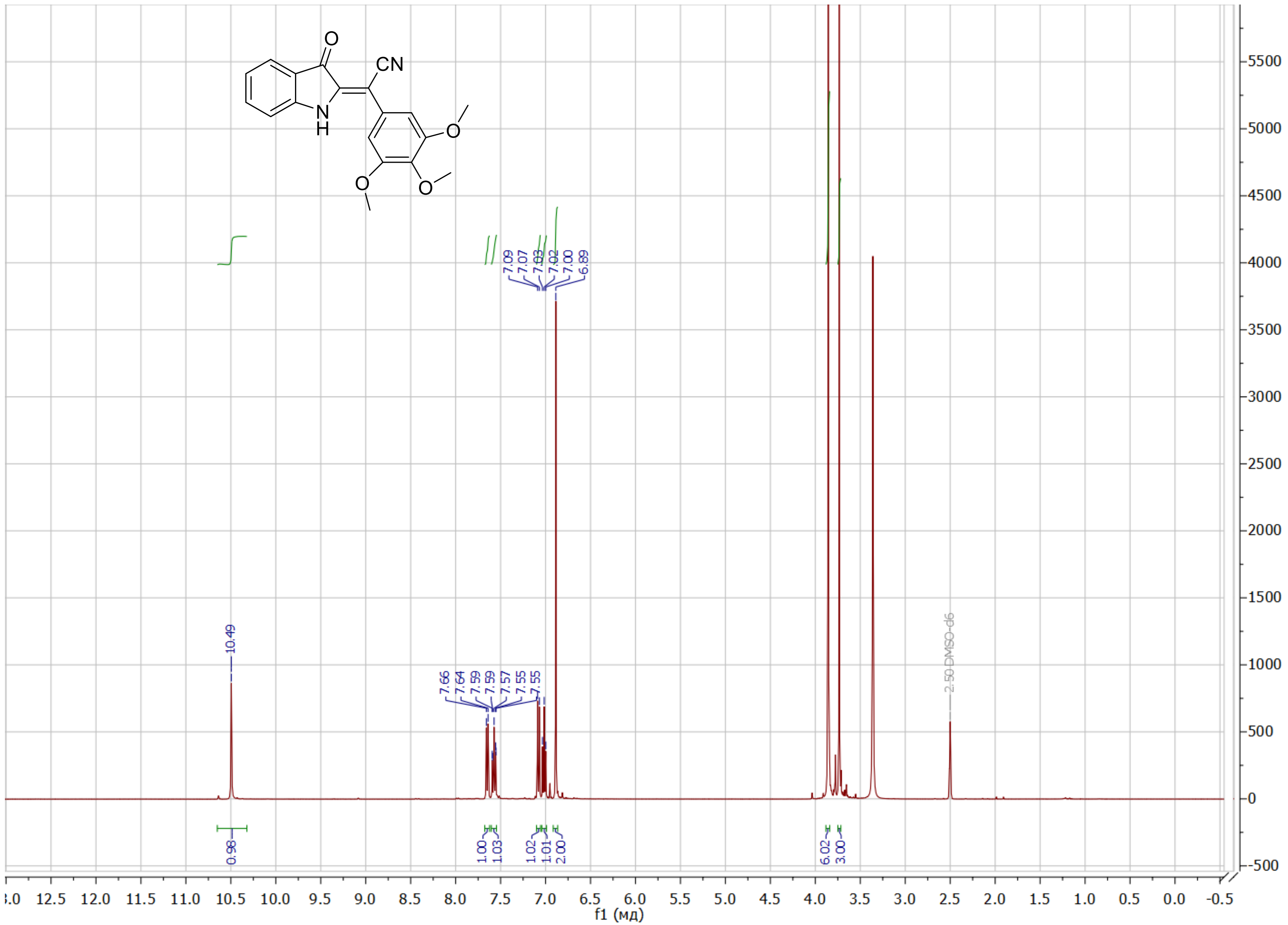

Figure S25. ^{1}H NMR spectrum of **2am** in DMSO-d_6 (400 MHz)

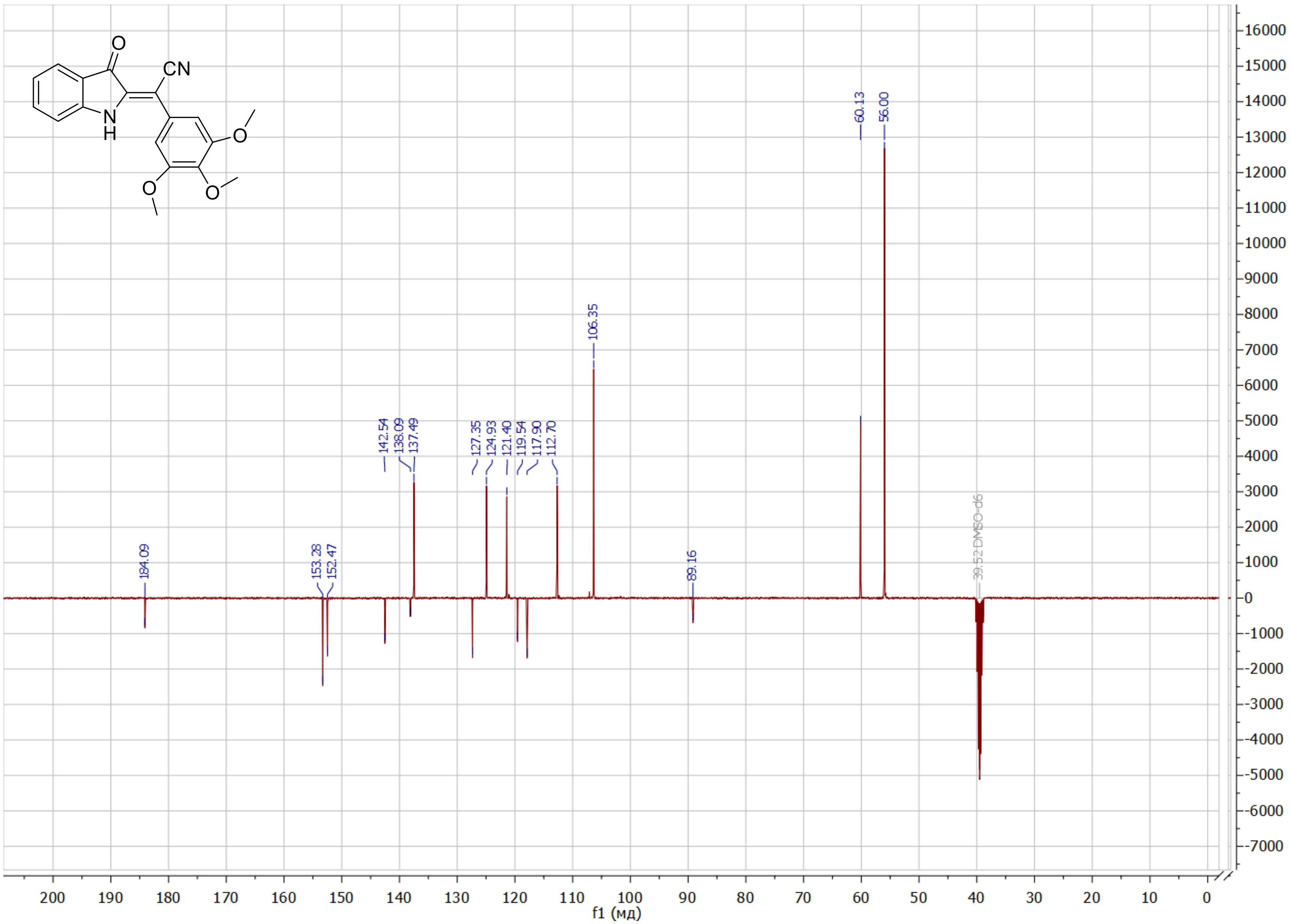

Figure S26. ^{13}C DEPTQ NMR spectrum of **2am** in DMSO-d_6 (100 MHz)

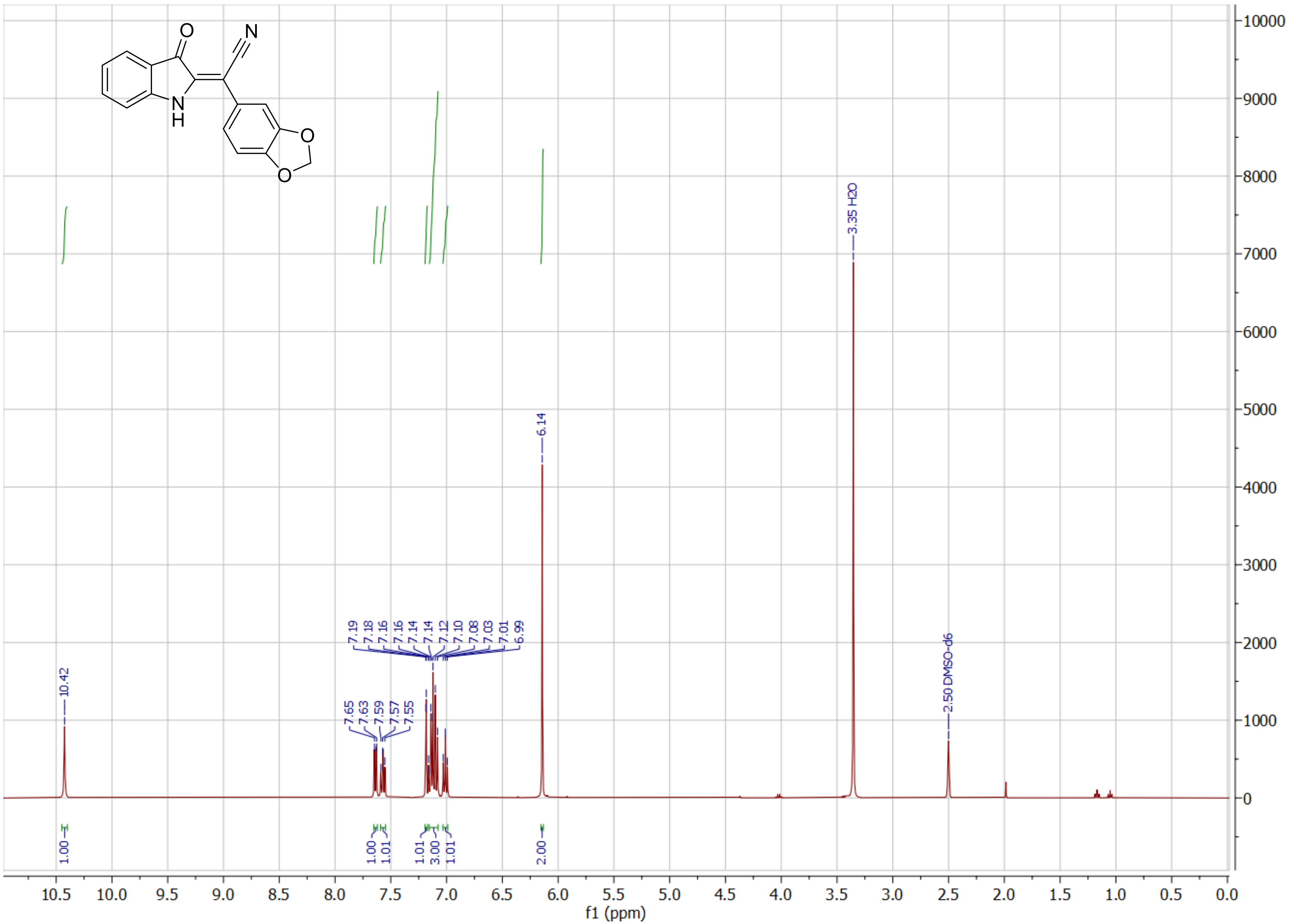

Figure S27. ^{1}H NMR spectrum of **2an** in DMSO-d_6 (400 MHz)

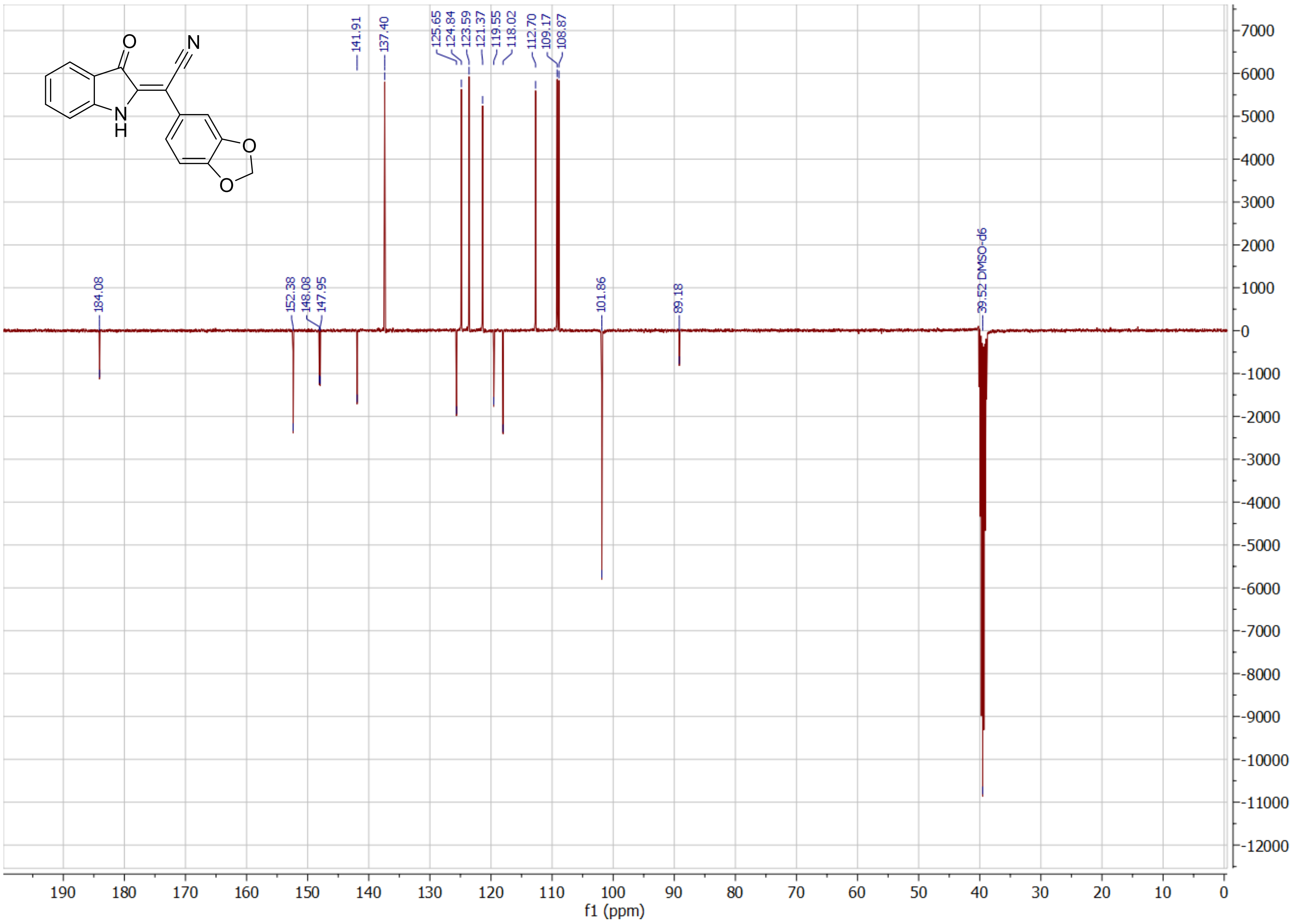

Figure S28. ^{13}C DEPTQ NMR spectrum of **2an** in DMSO-d_6 (100 MHz)

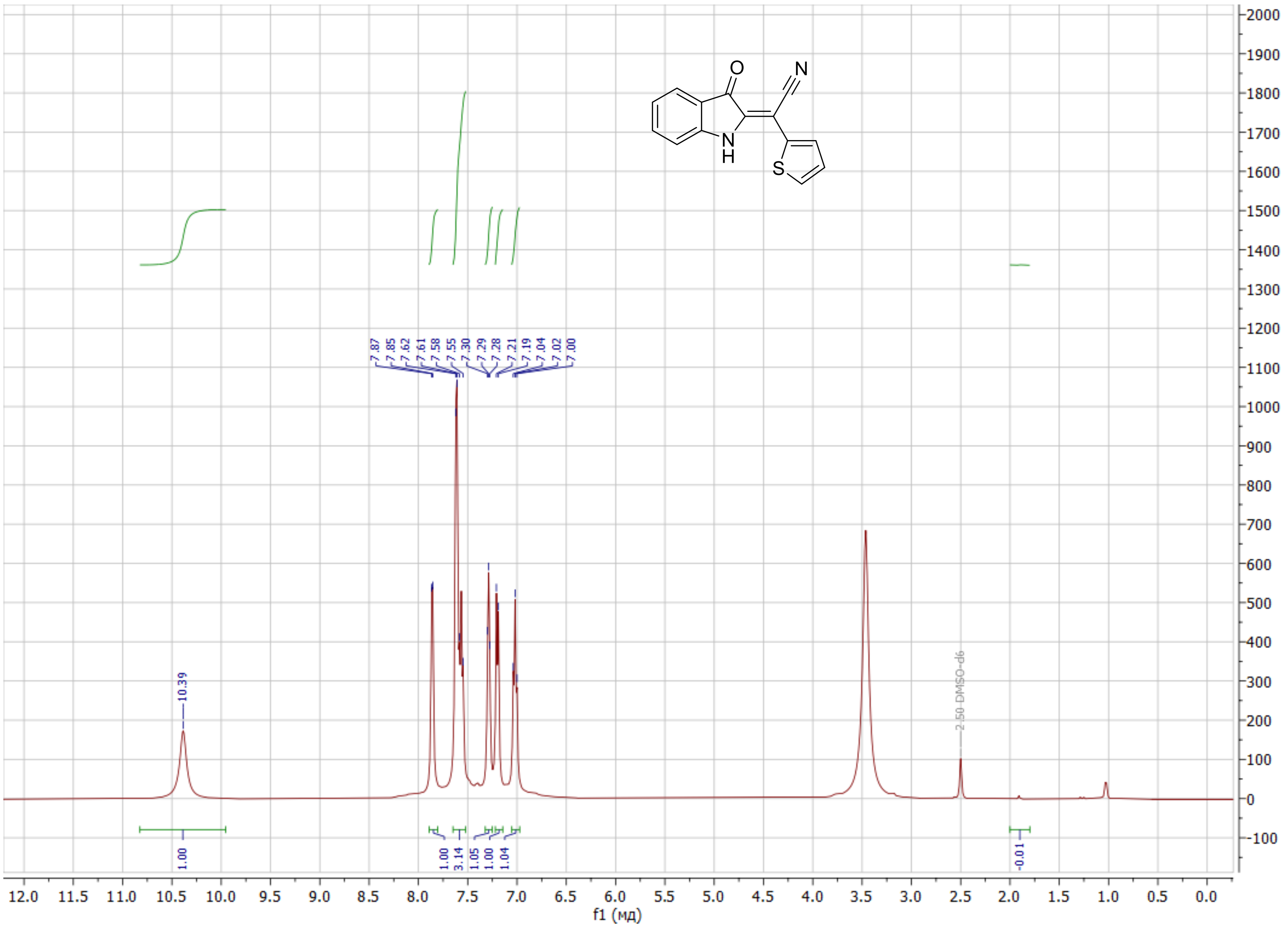

Figure S29. ^{1}H NMR spectrum of **2ao** in DMSO-d_6 (400 MHz)

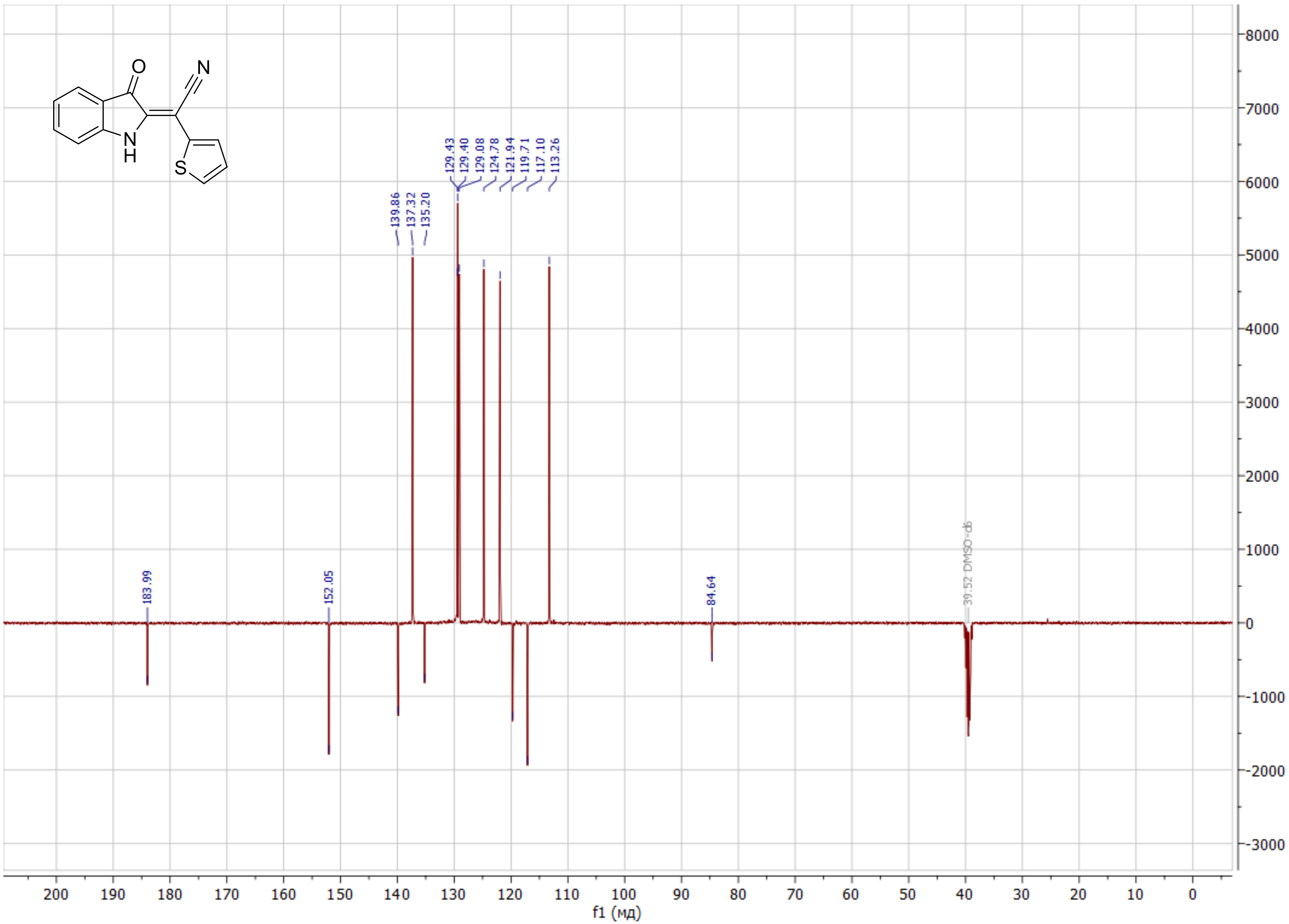

Figure S30. ^{13}C DEPTQ NMR spectrum of **2ao** in DMSO-d_6 (100 MHz)

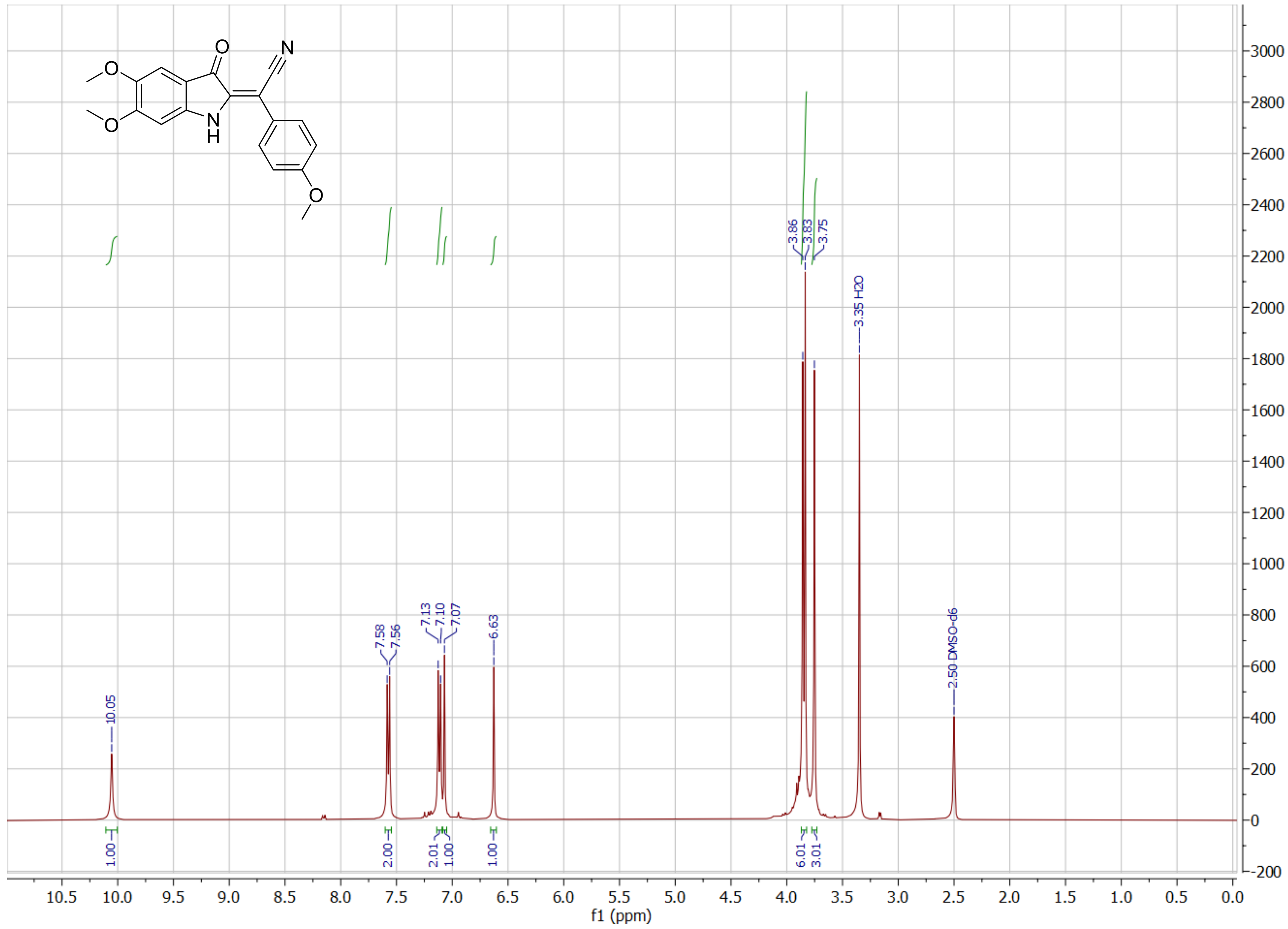

Figure S31. ^{1}H NMR spectrum of **2ba** in DMSO-d_6 (400 MHz)

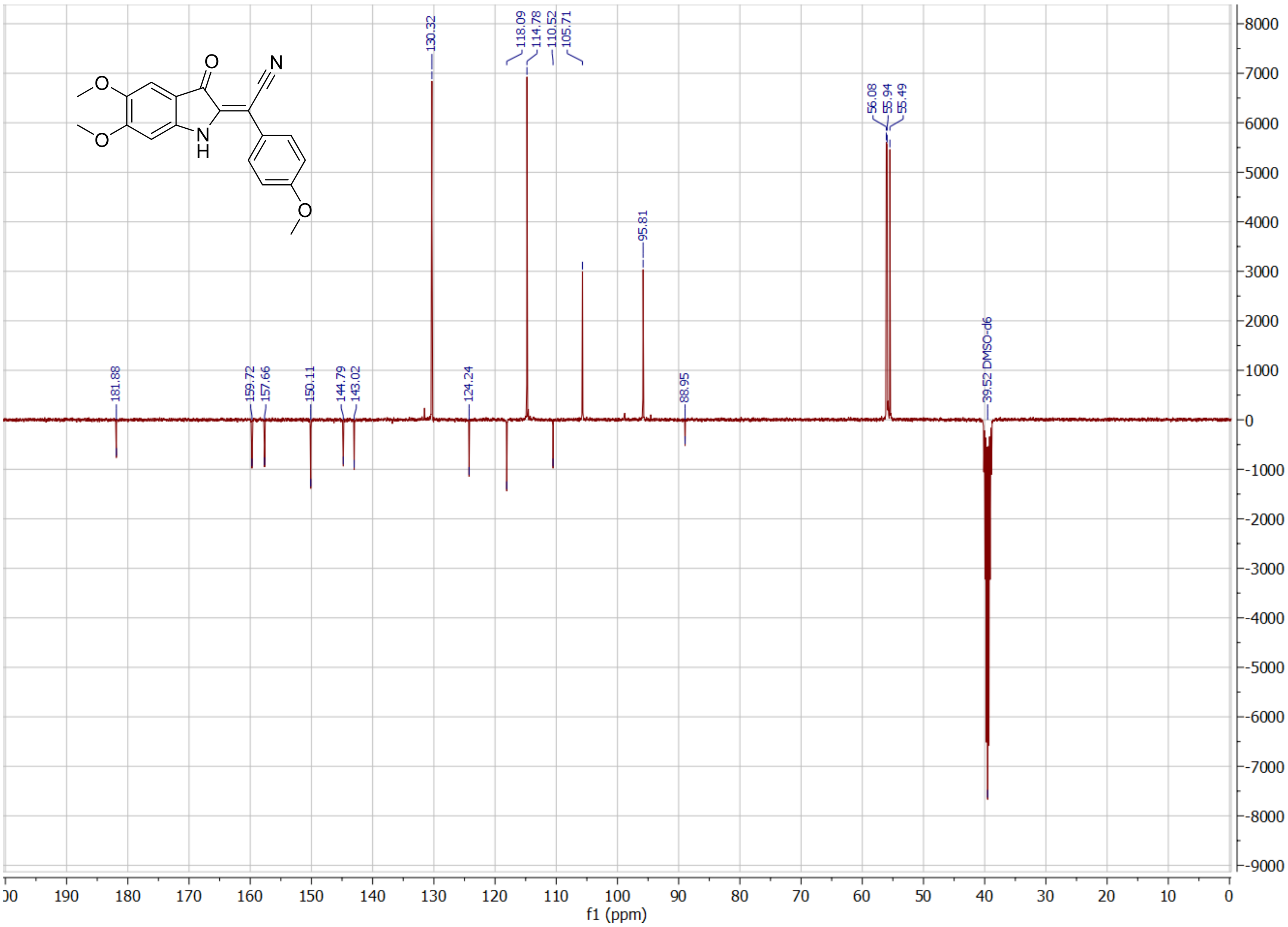

Figure S32. ^{13}C DEPTQ NMR spectrum of **2ba** in DMSO-d_6 (100 MHz)

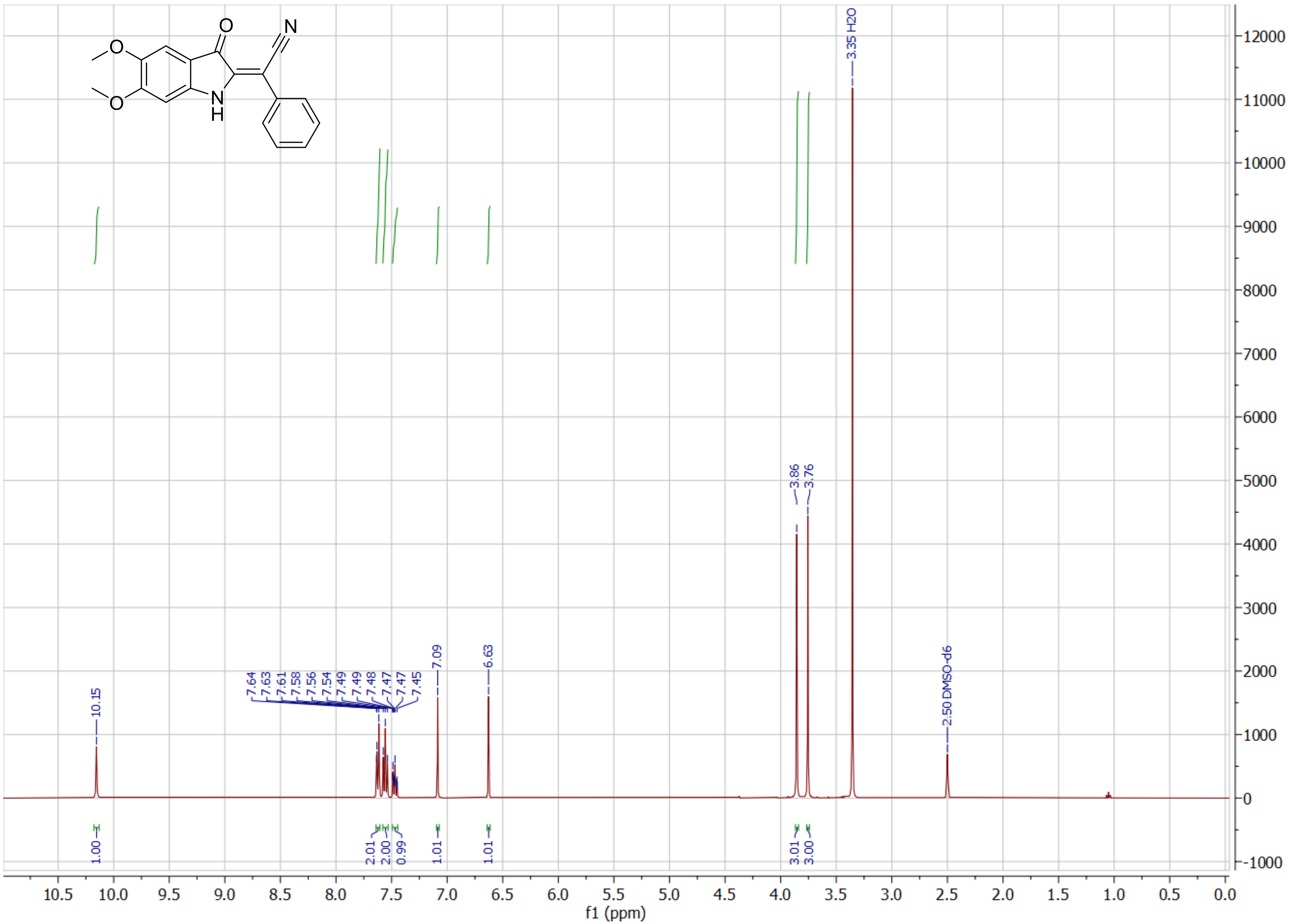

Figure S33. ^{1}H NMR spectrum of **2bb** in DMSO-d_6 (400 MHz)

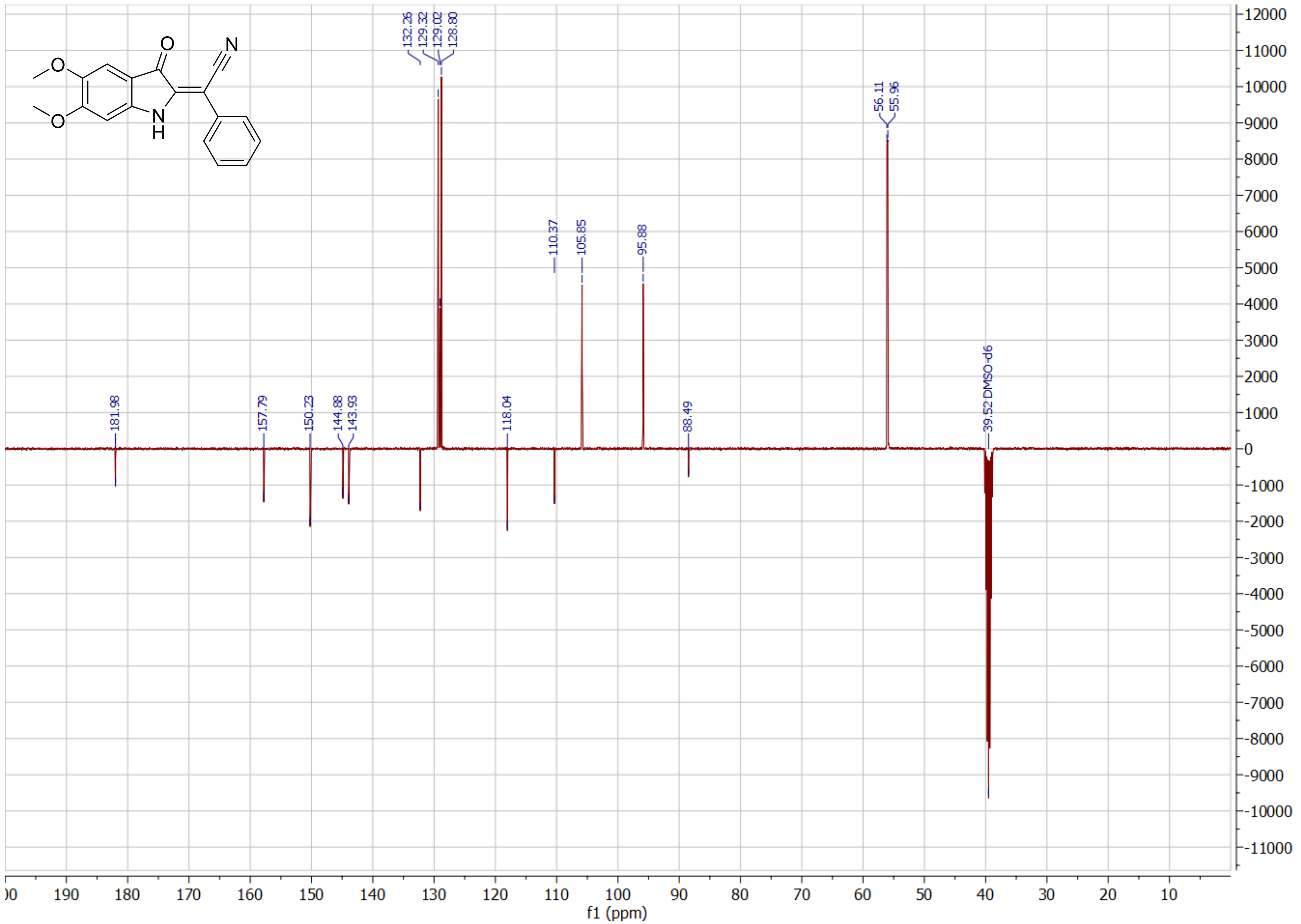

Figure S34. ^{13}C DEPTQ NMR spectrum of **2bb** in DMSO-d_6 (100 MHz)

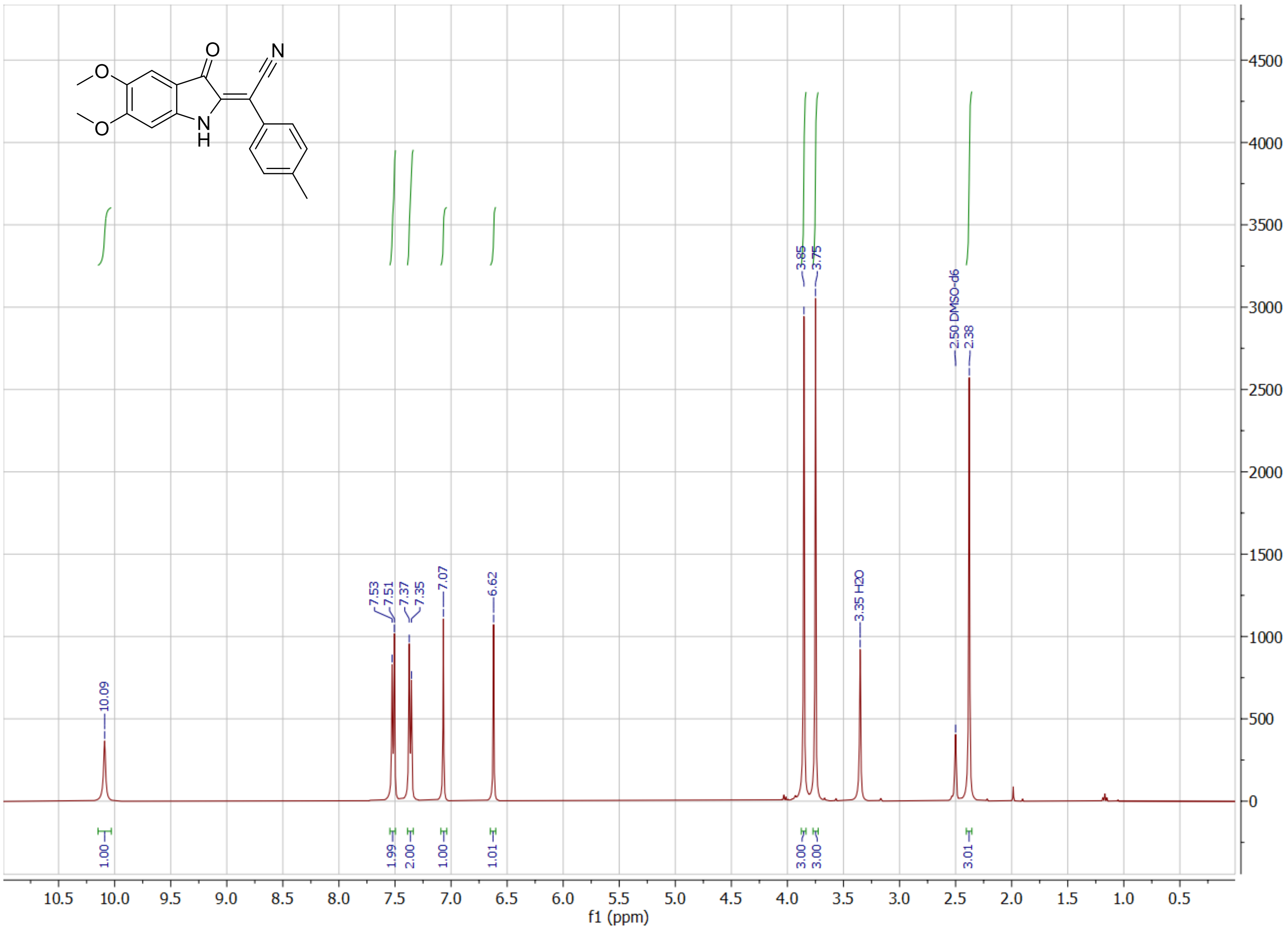

Figure S35. ^{1}H NMR spectrum of **2bc** in DMSO-d_6 (400 MHz)

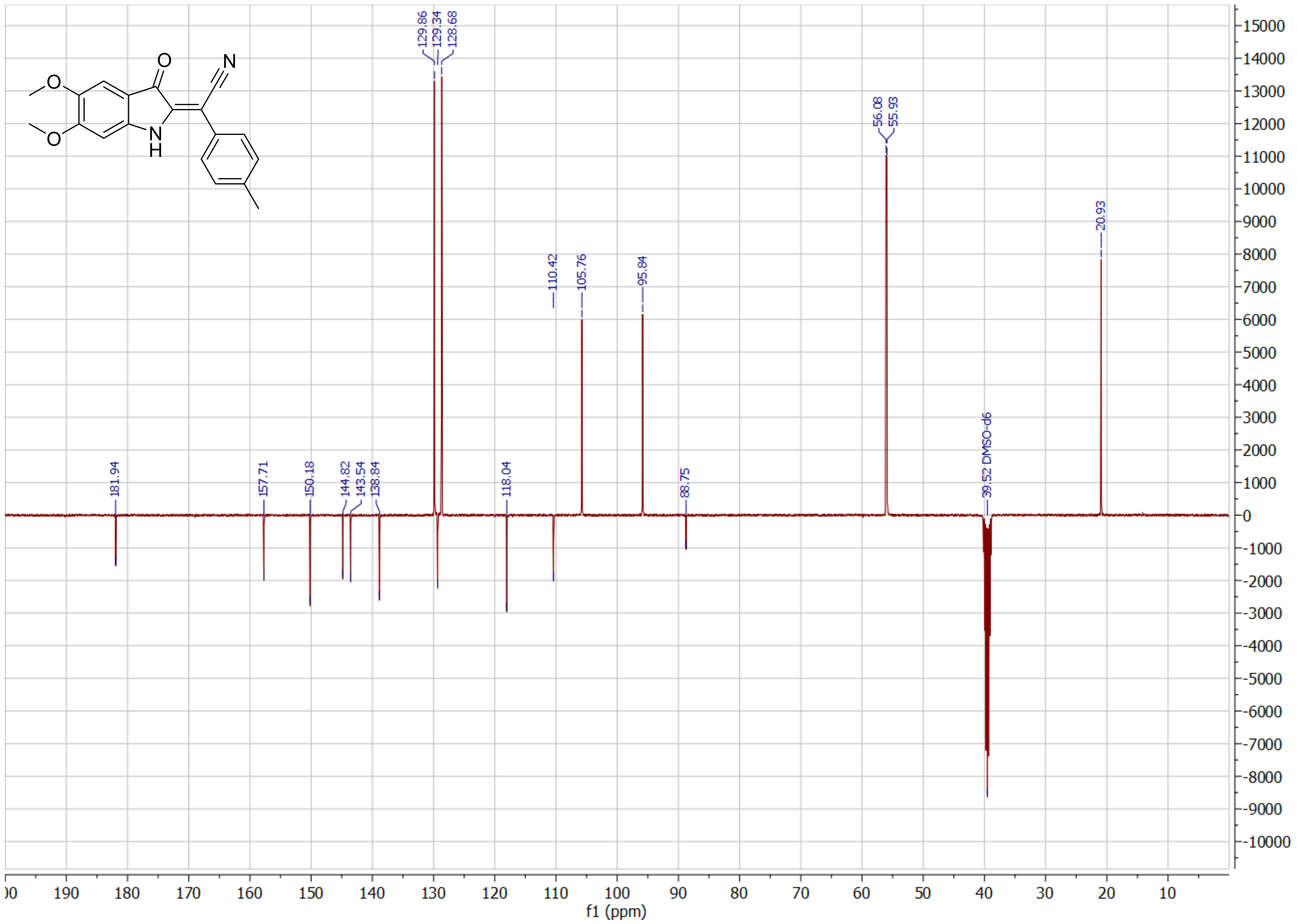

Figure S36. ^{13}C DEPTQ NMR spectrum of **2bc** in DMSO-d_6 (100 MHz)

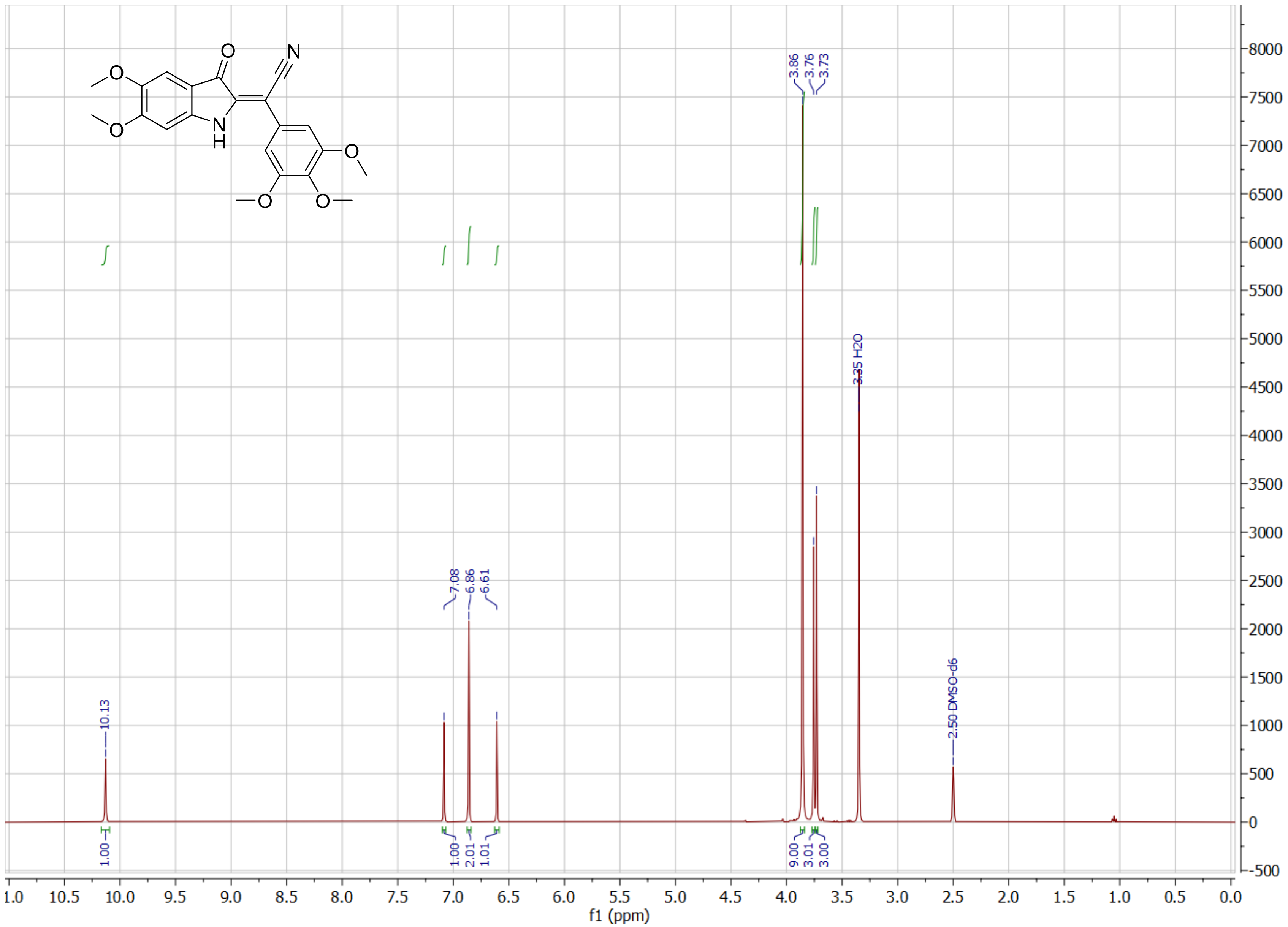

Figure S37. ^{1}H NMR spectrum of **2bm** in DMSO-d_6 (400 MHz)

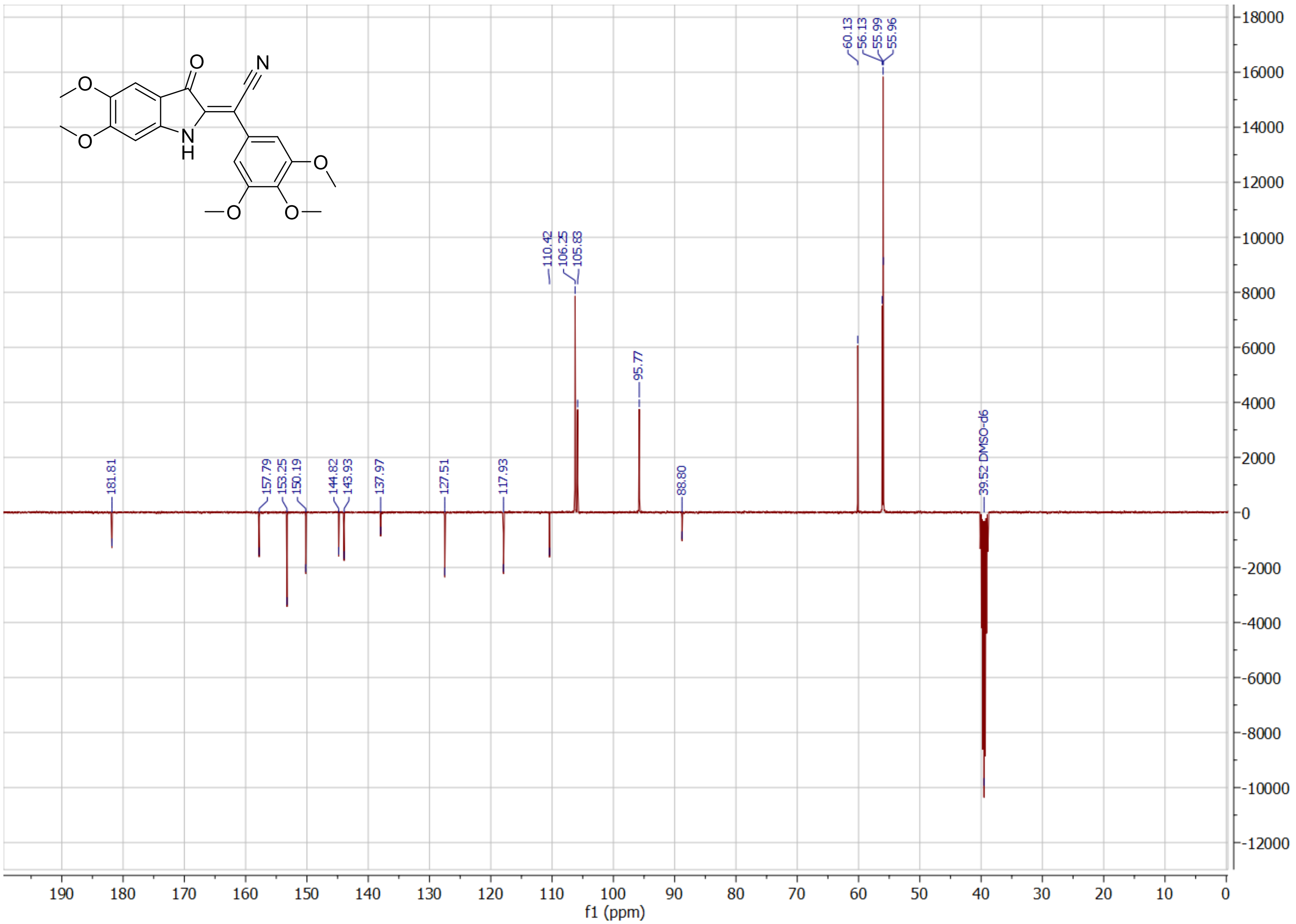

Figure S38. ^{13}C DEPTQ NMR spectrum of **2bm** in DMSO-d_6 (100 MHz)

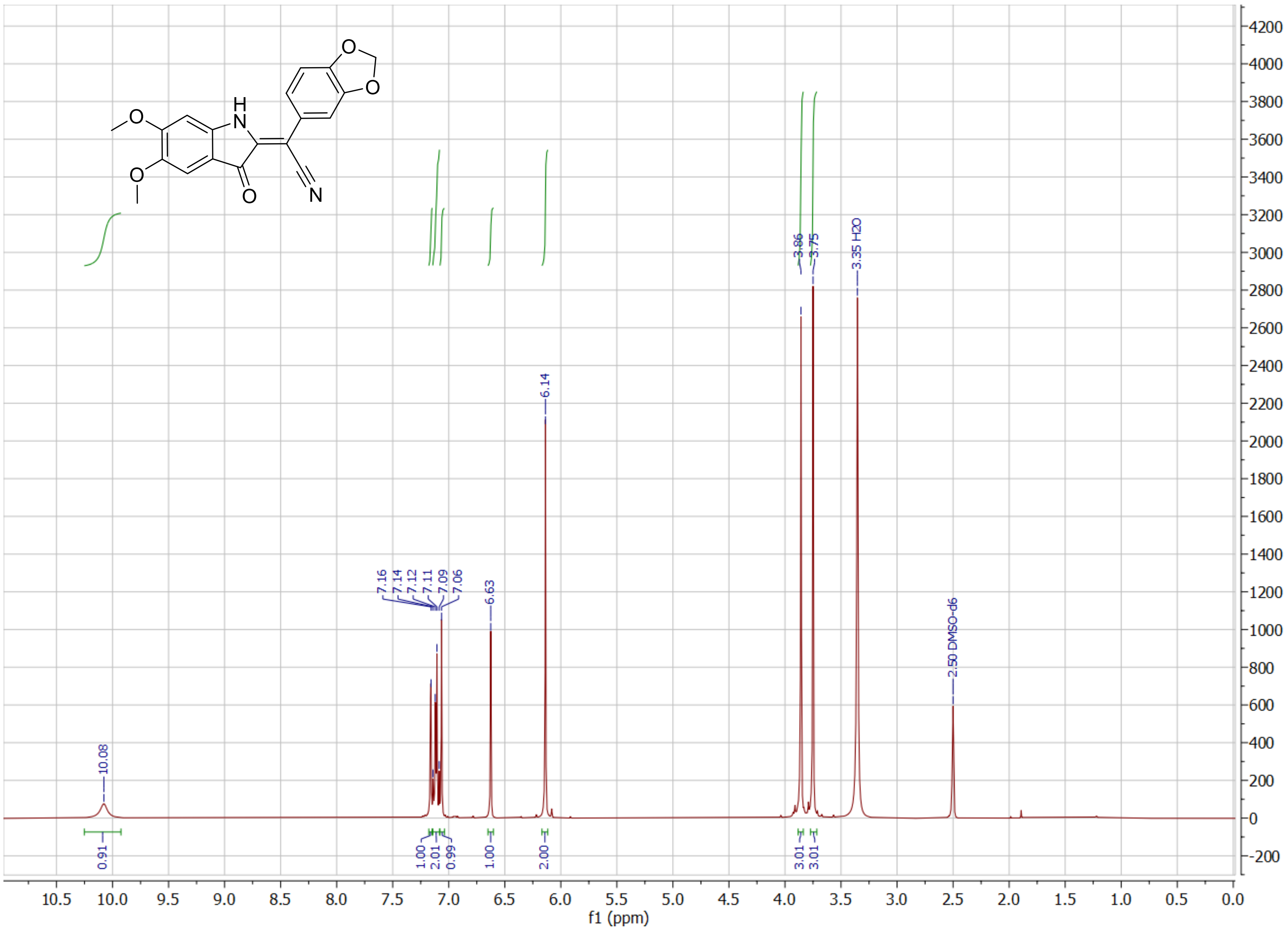

Figure S39. ^{1}H NMR spectrum of **2bn** in DMSO-d_6 (400 MHz)

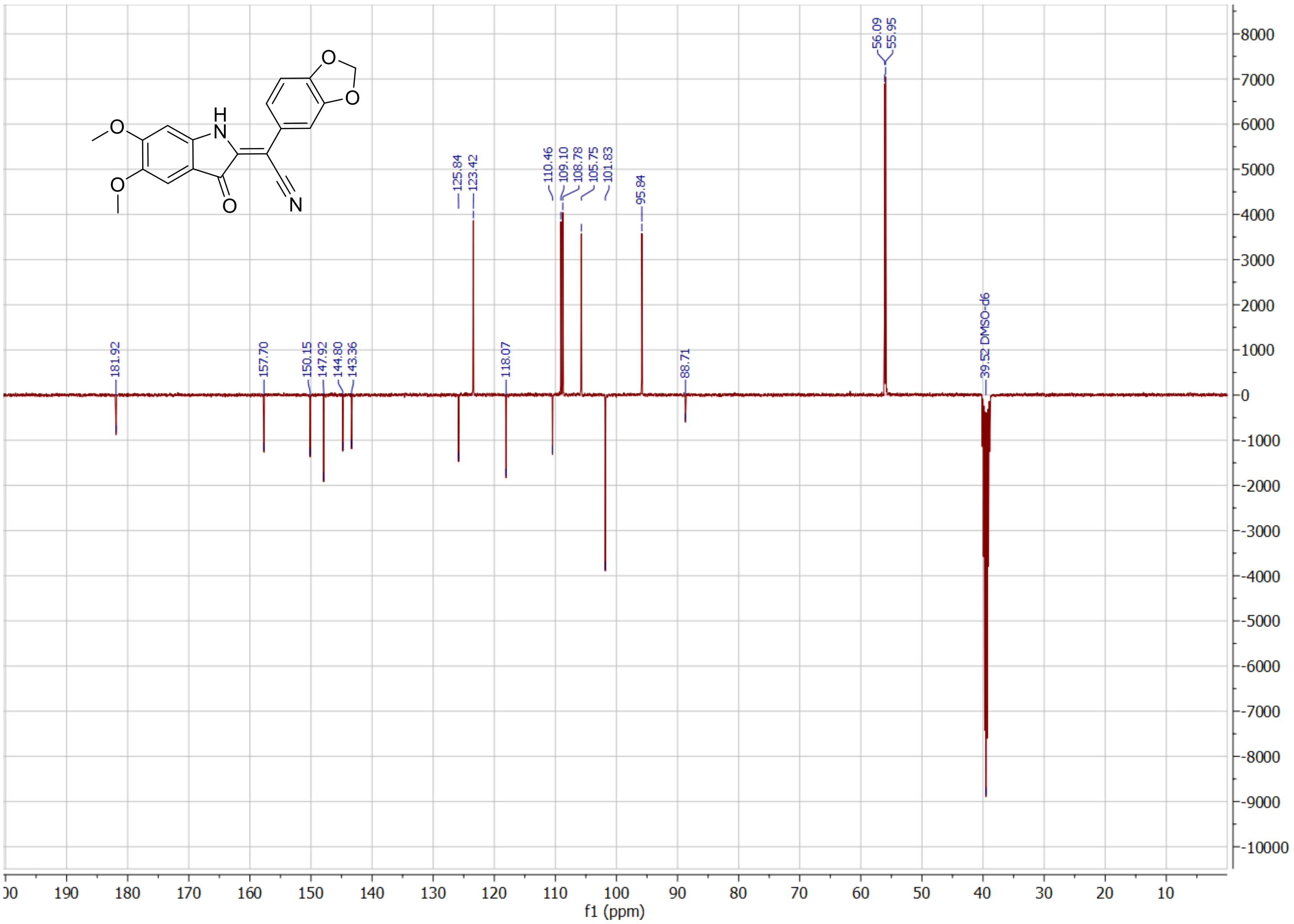

Figure S40. ^{13}C DEPTQ NMR spectrum of **2bn** in DMSO-d_6 (100 MHz)

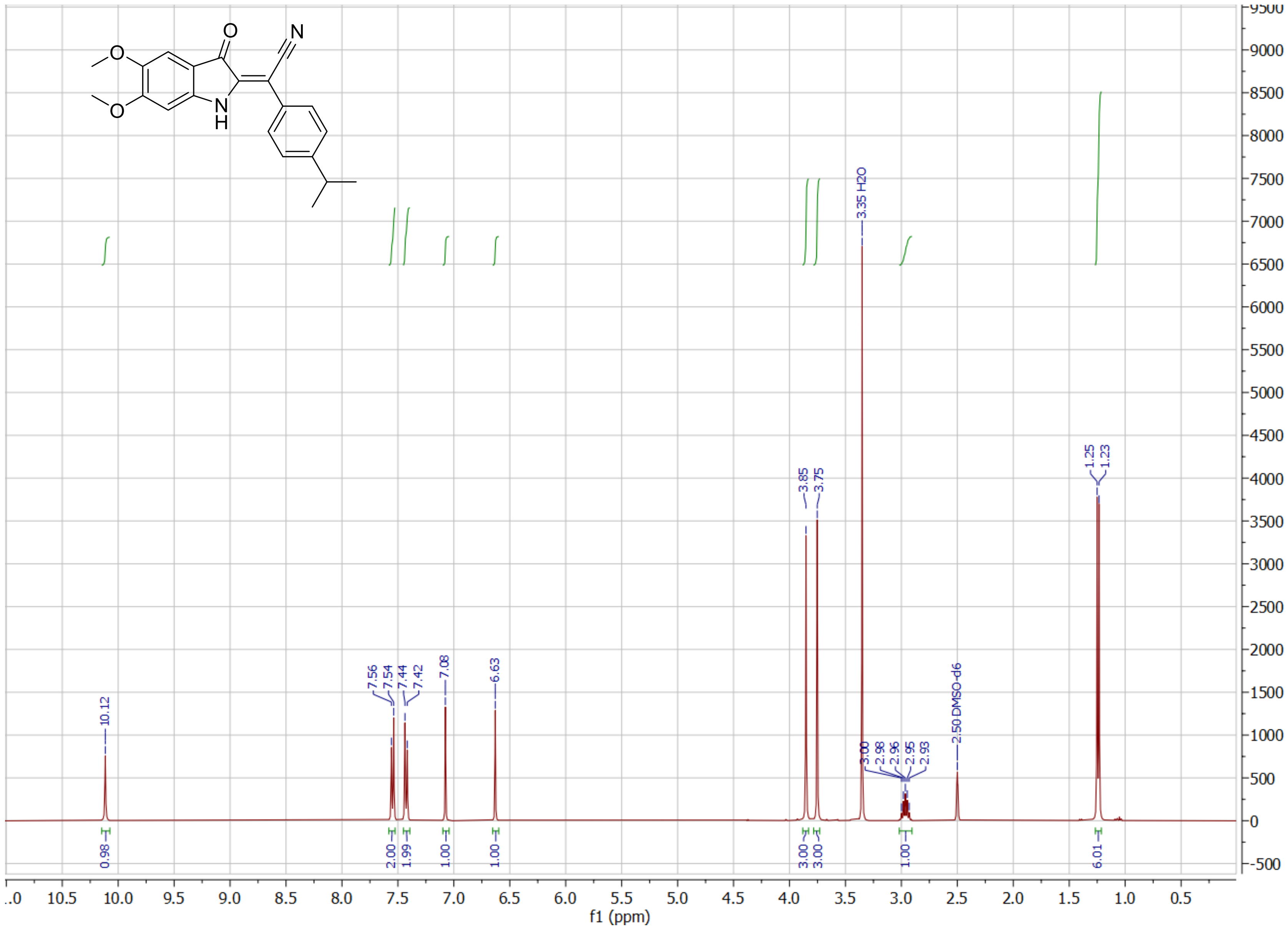

Figure S41. ^{1}H NMR spectrum of **2be** in DMSO-d_6 (400 MHz)

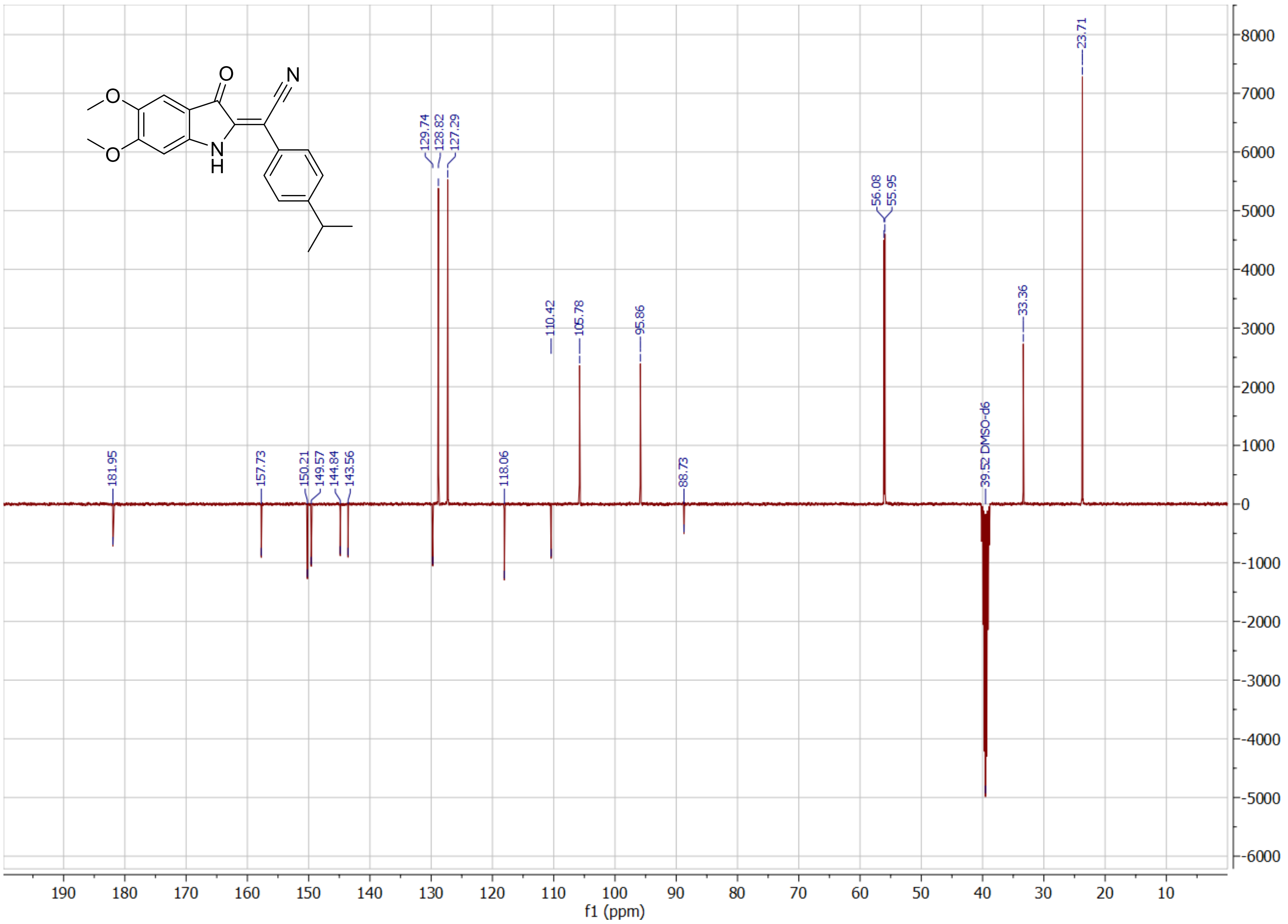

Figure S42. ^{13}C DEPTQ NMR spectrum of **2be** in DMSO-d_6 (100 MHz)

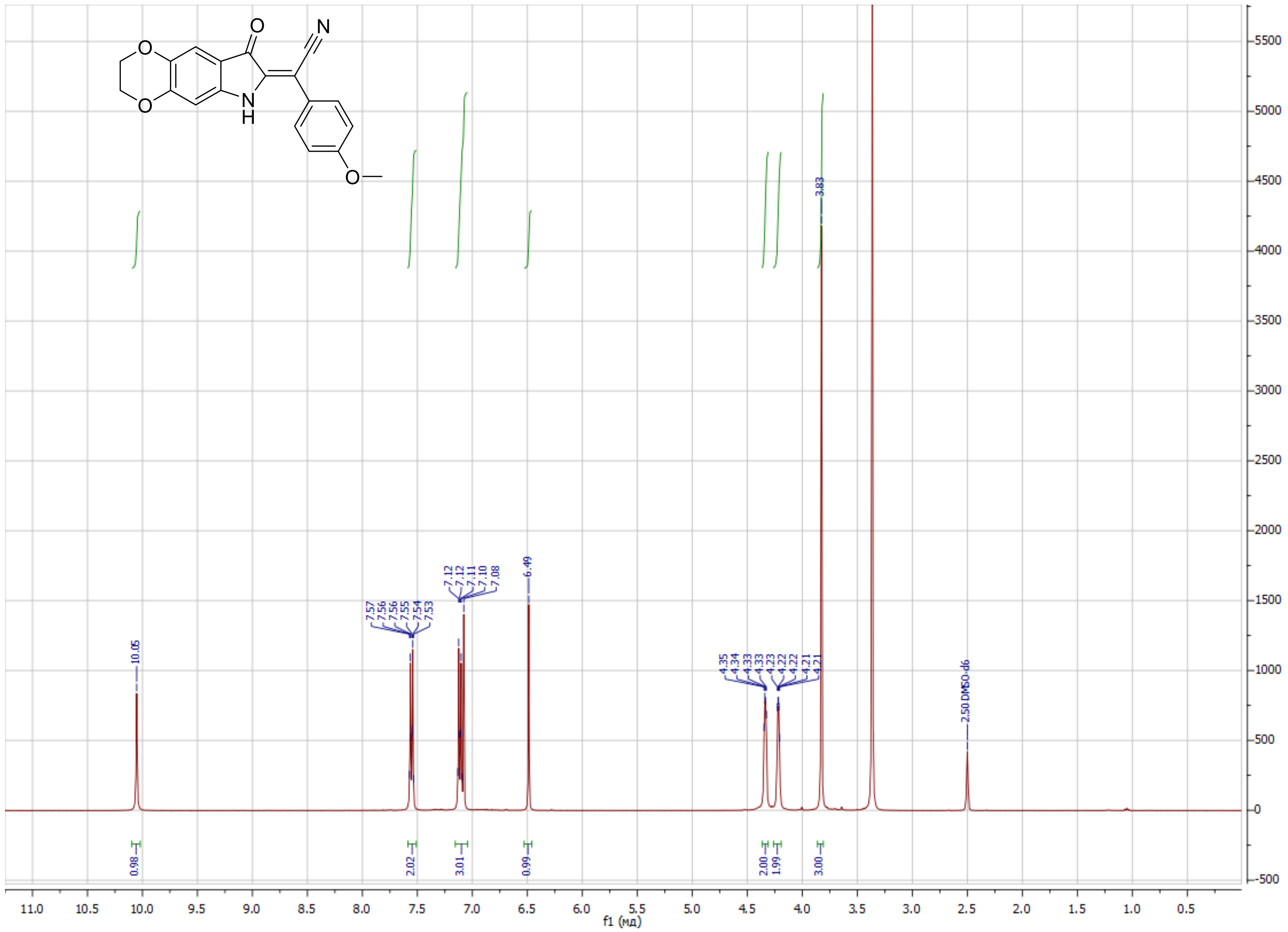

Figure S43. ^{1}H NMR spectrum of **2ca** in DMSO-d_6 (400 MHz)

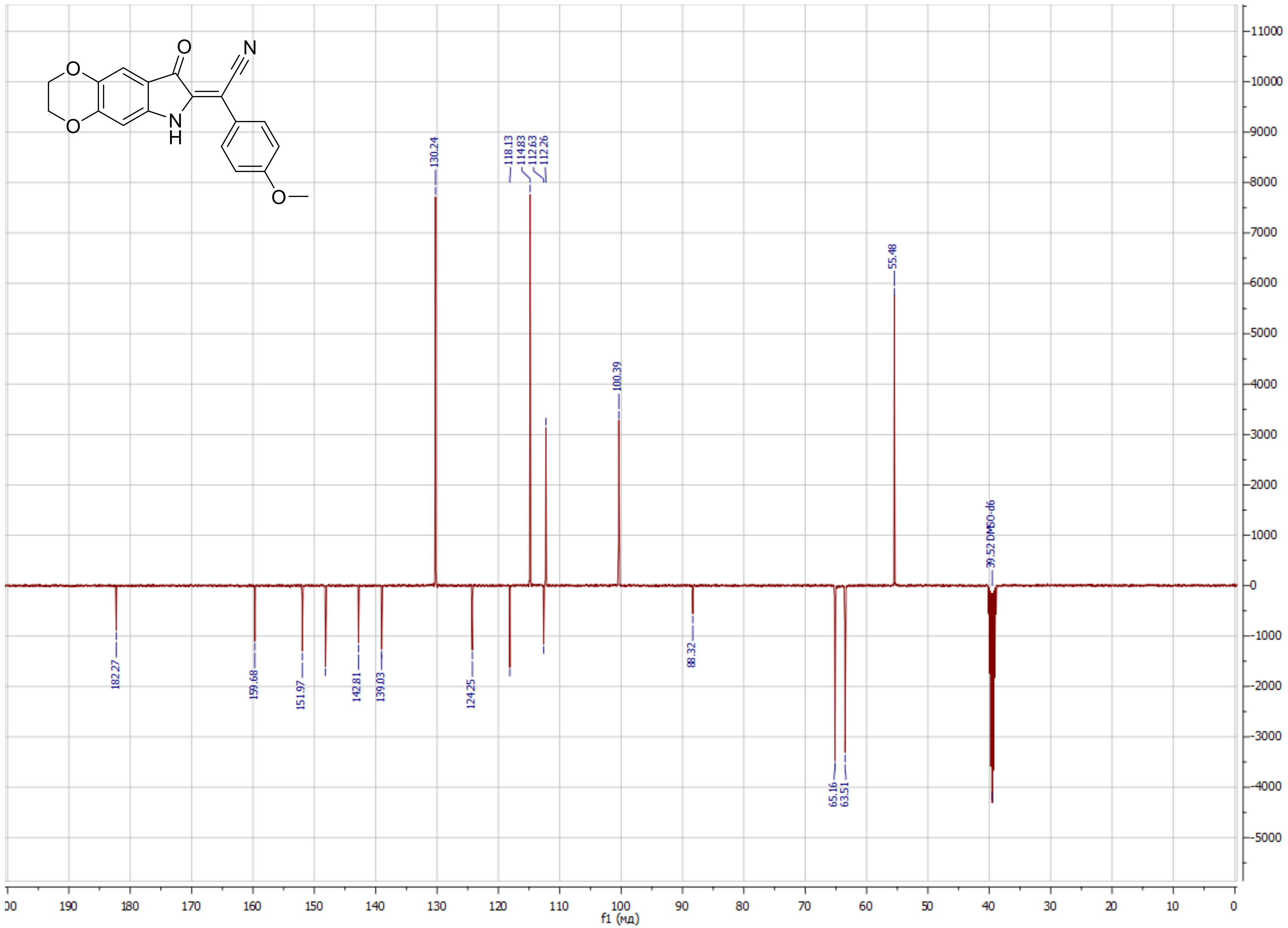

Figure S44. ^{13}C DEPTQ NMR spectrum of **2ca** in DMSO-d_6 (100 MHz)

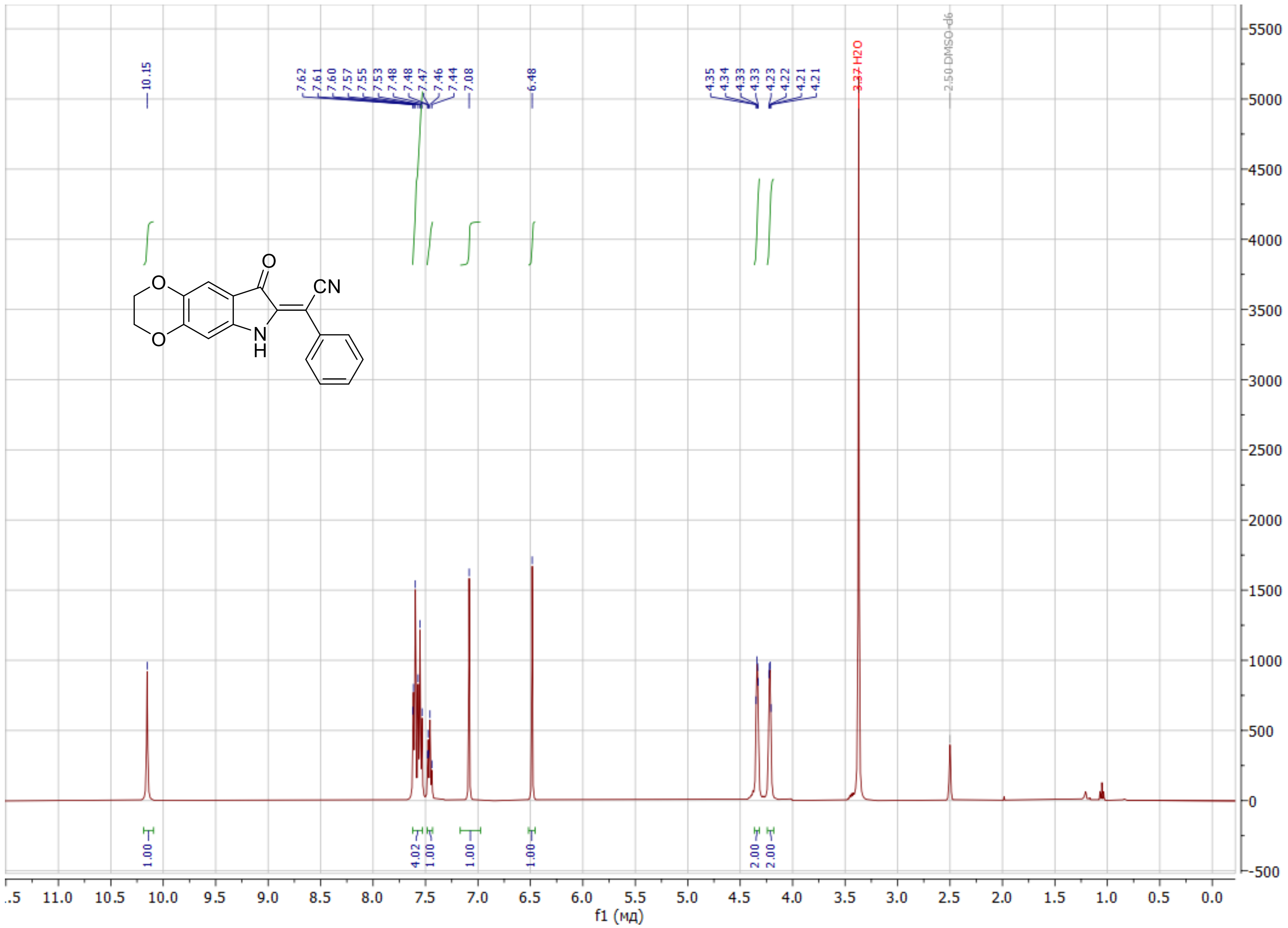

Figure S45. ^{1}H NMR spectrum of **2cb** in DMSO-d_6 (400 MHz)

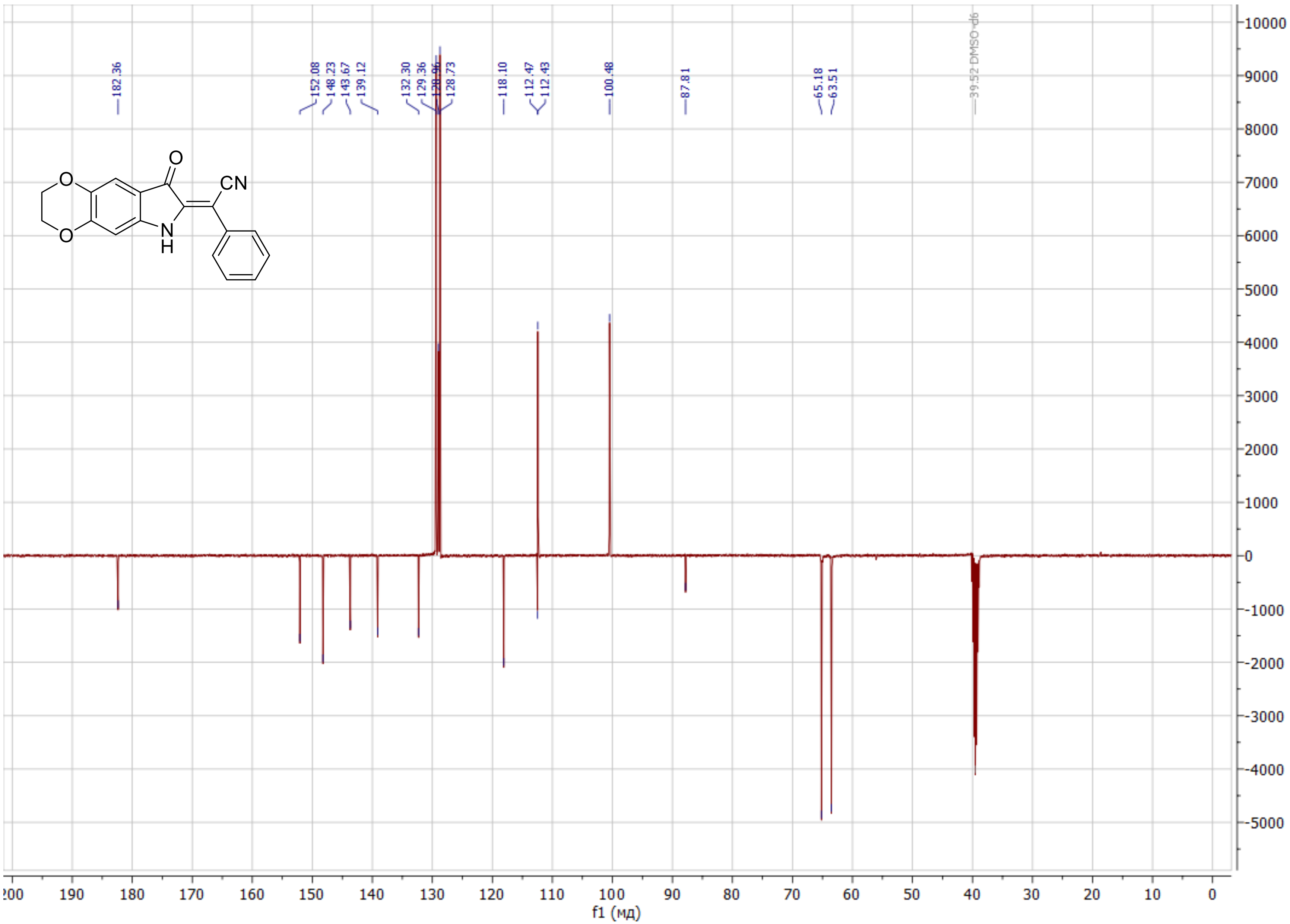

Figure S46. ^{13}C DEPTQ NMR spectrum of **2cb** in DMSO-d_6 (100 MHz)

^{1}H and ^{13}C NMR spectral charts for(*E*)-2-(1-hydroxy-3-oxoindolin-2-ylidene)-2-phenylacetonitrile (10ab):

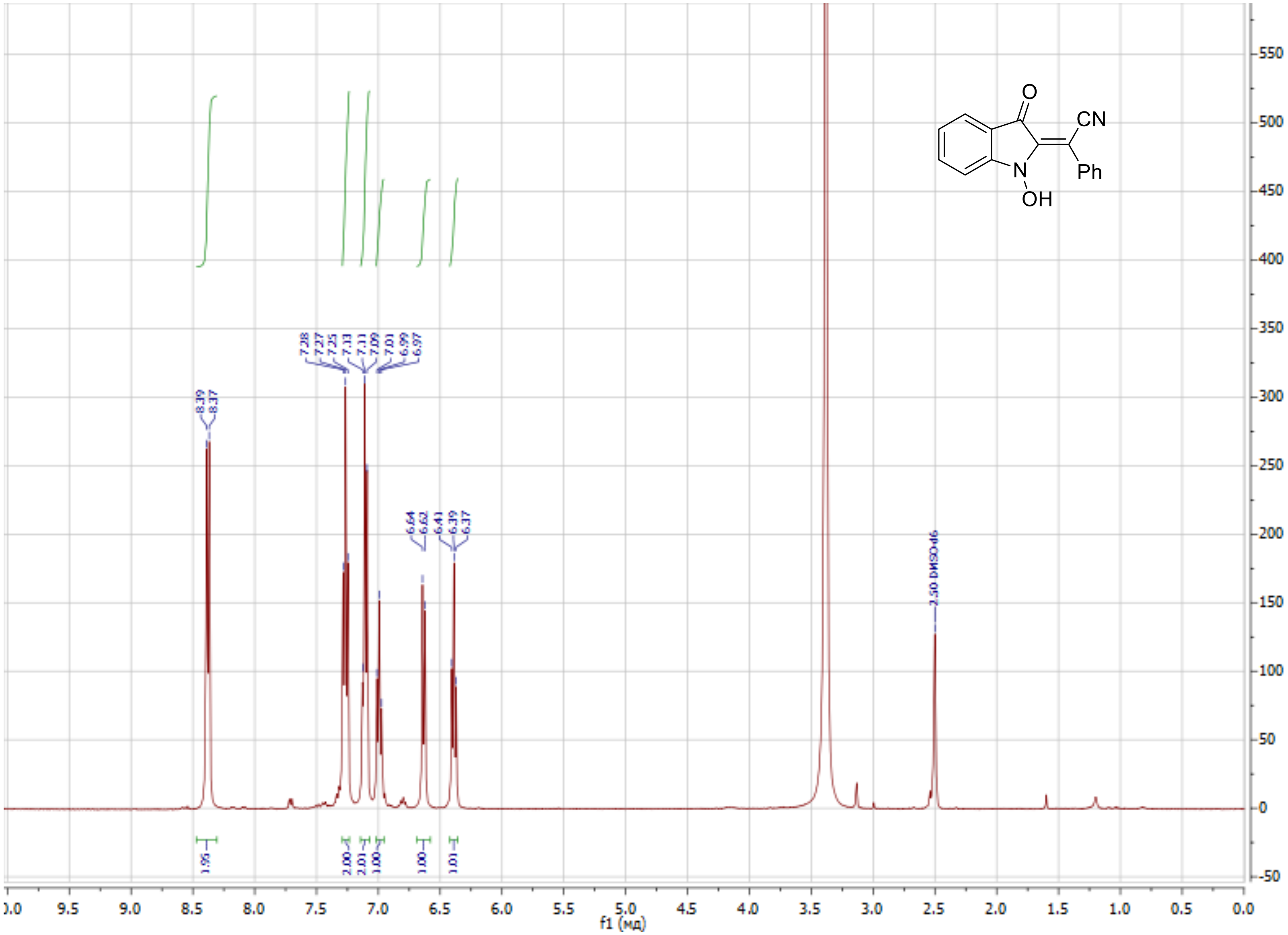

Figure S47. ^{1}H NMR spectrum of **10ab** in DMSO-d_6 (400 MHz)

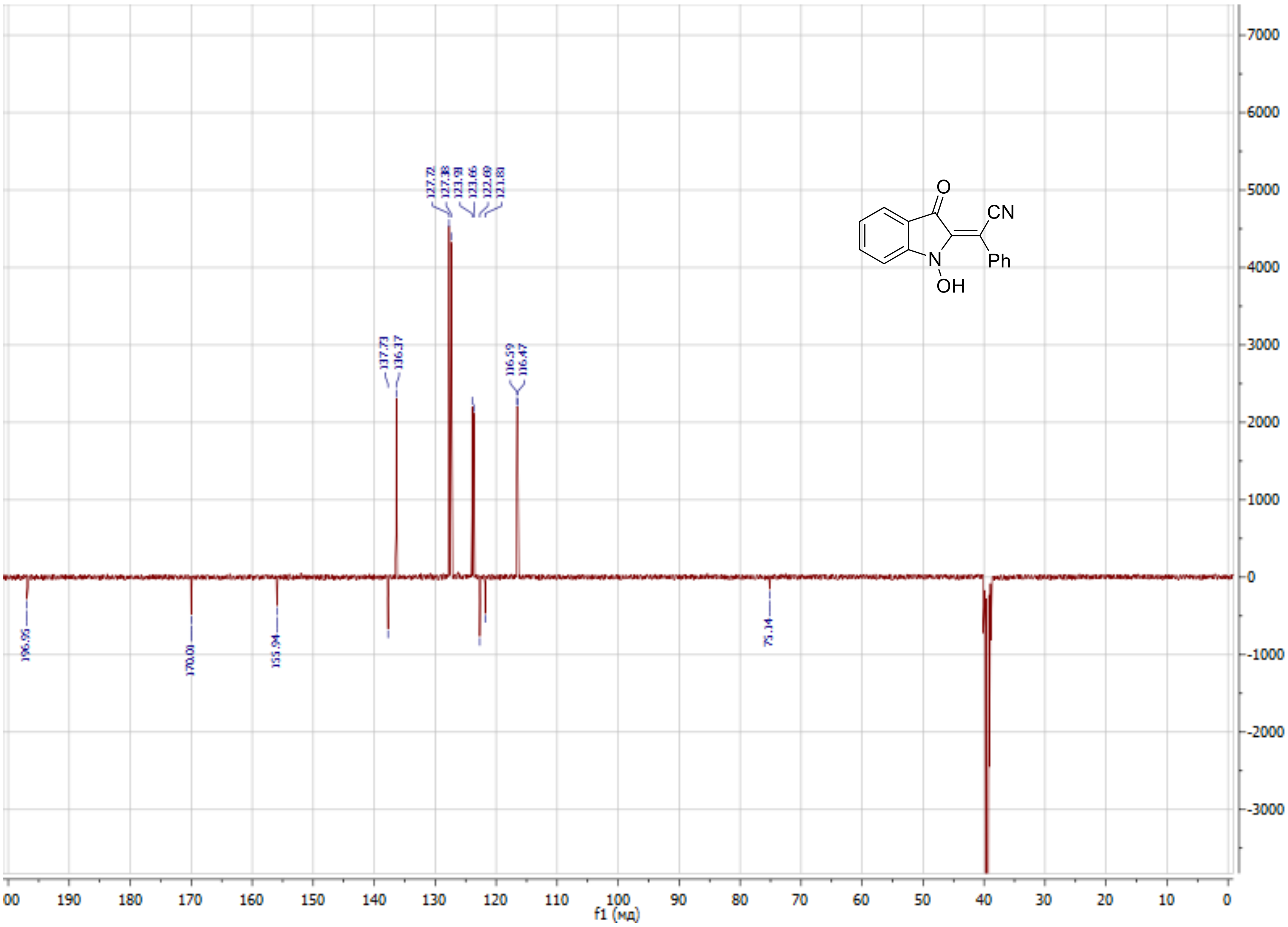

Figure S48. ^{13}C DEPTQ NMR spectrum of 10ab in DMSO-d_6 (100 MHz)

^{1}H and ^{13}C NMR spectral charts for 4-oxo-2,4-diphenylbutanenitrile (12):

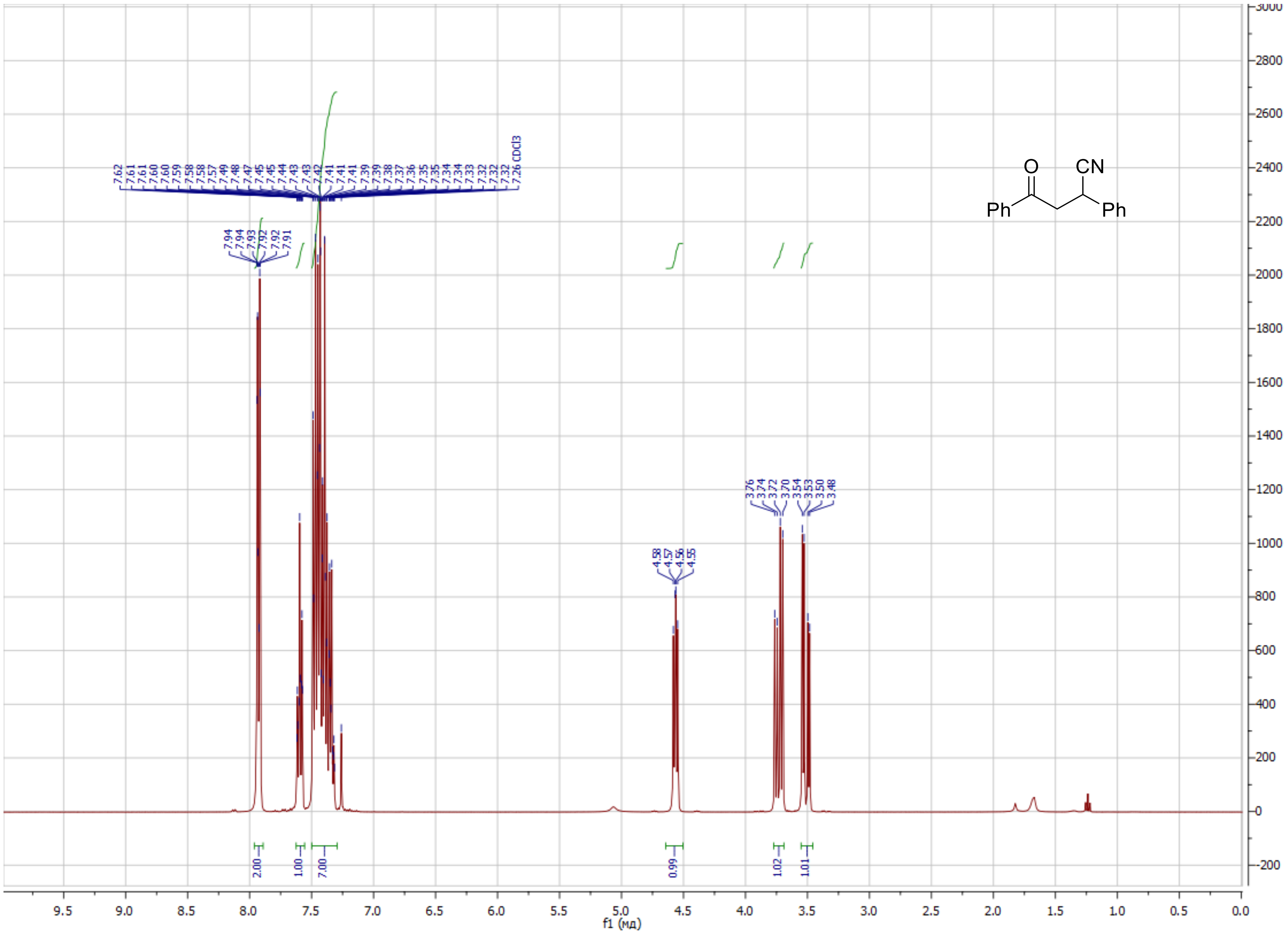

Figure S49. ^{1}H NMR spectrum of 12 in DMSO-d_6 (400 MHz)

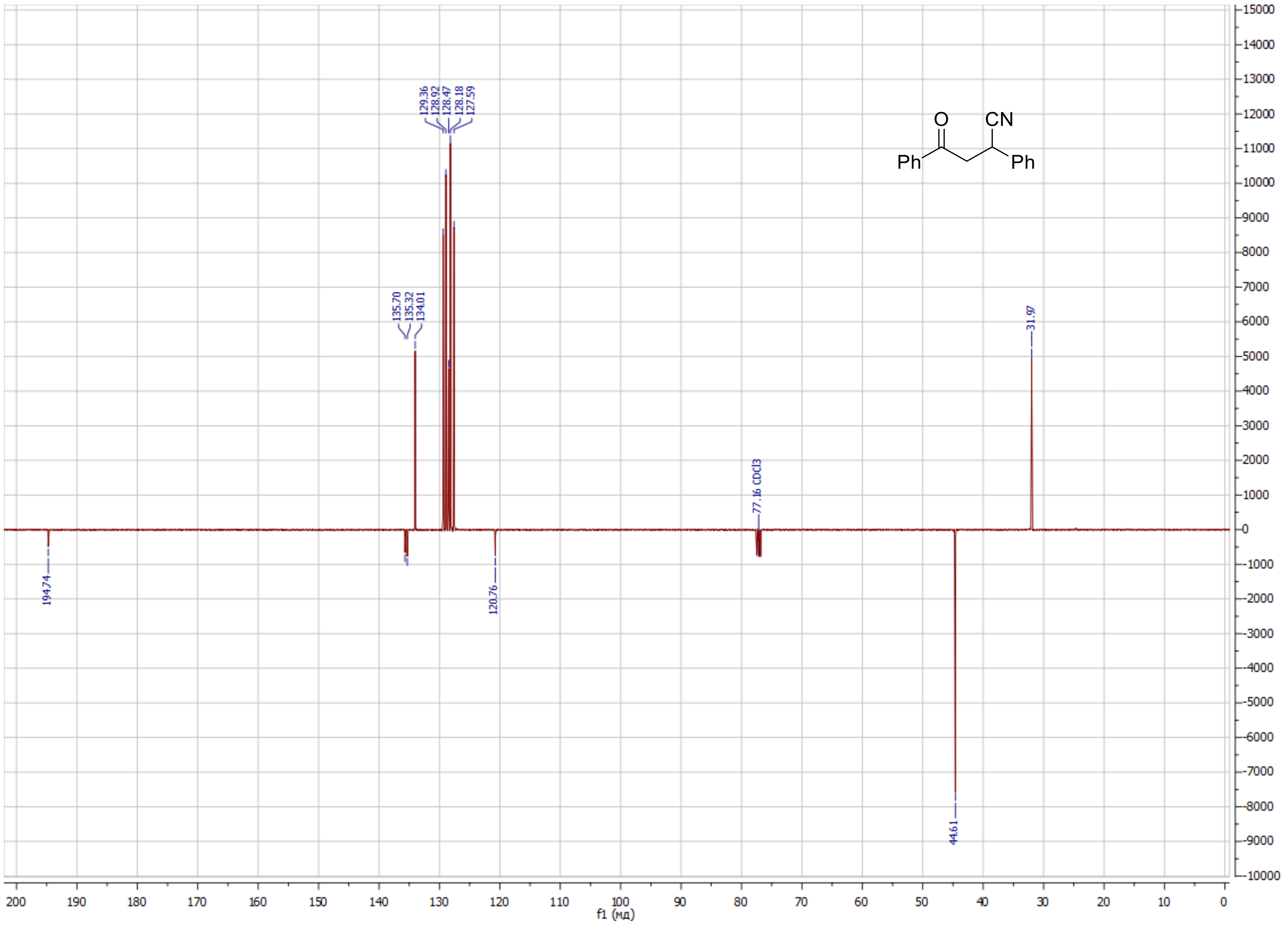

Figure S50. ^{13}C DEPTQ NMR spectrum of 12 in DMSO-d_6 (100 MHz)

^{1}H and ^{13}C NMR spectral charts for 5*b*-hydroxy-5*b*,10-dihydrobenzo[4',5']thieno[2',3':3,4]cyclopenta[1,2-*b*]indole-11-carbonitrile (2ap):

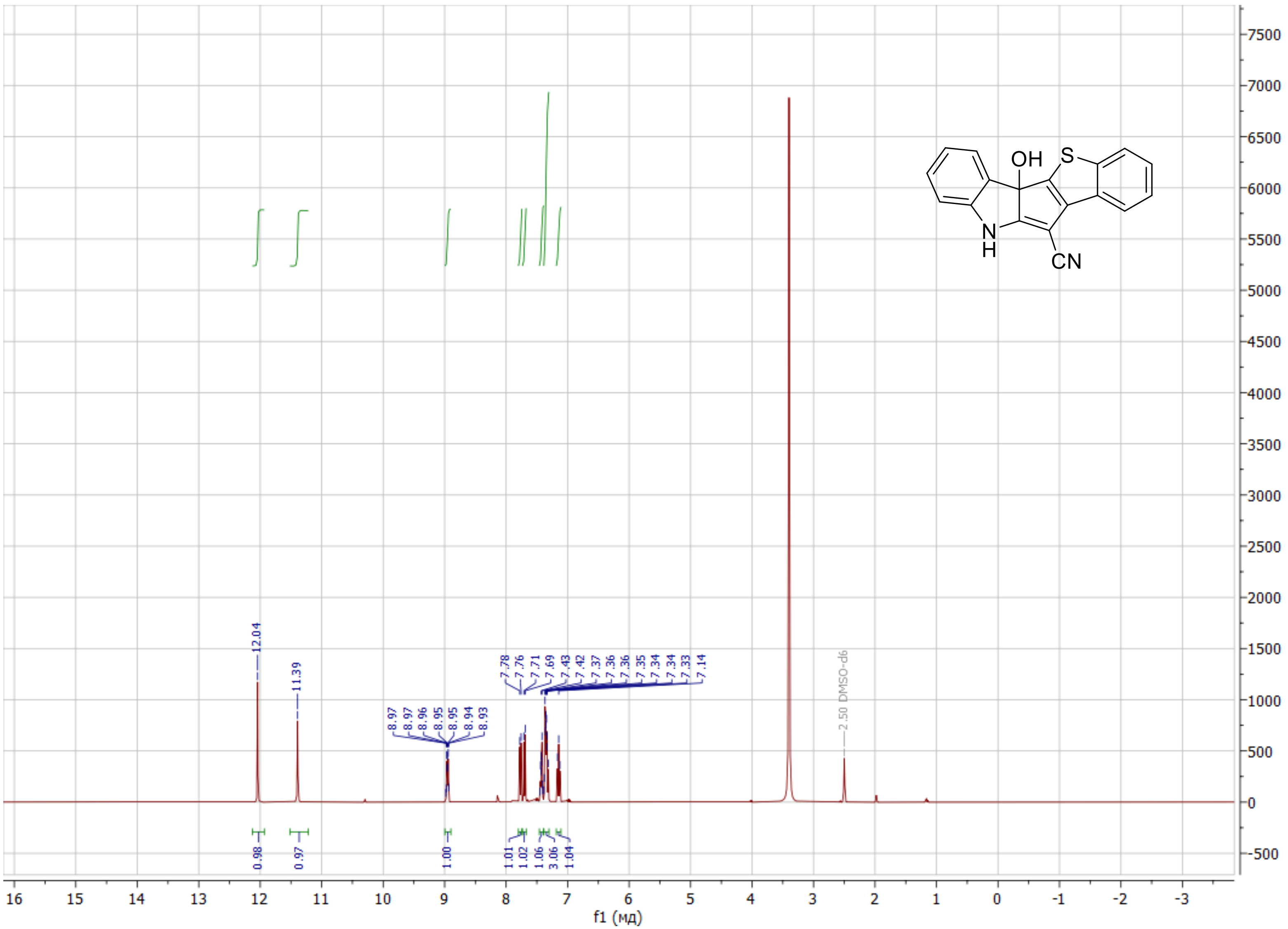

Figure S51. ^{1}H NMR spectrum of **2ap** in DMSO-d_6 (400 MHz)

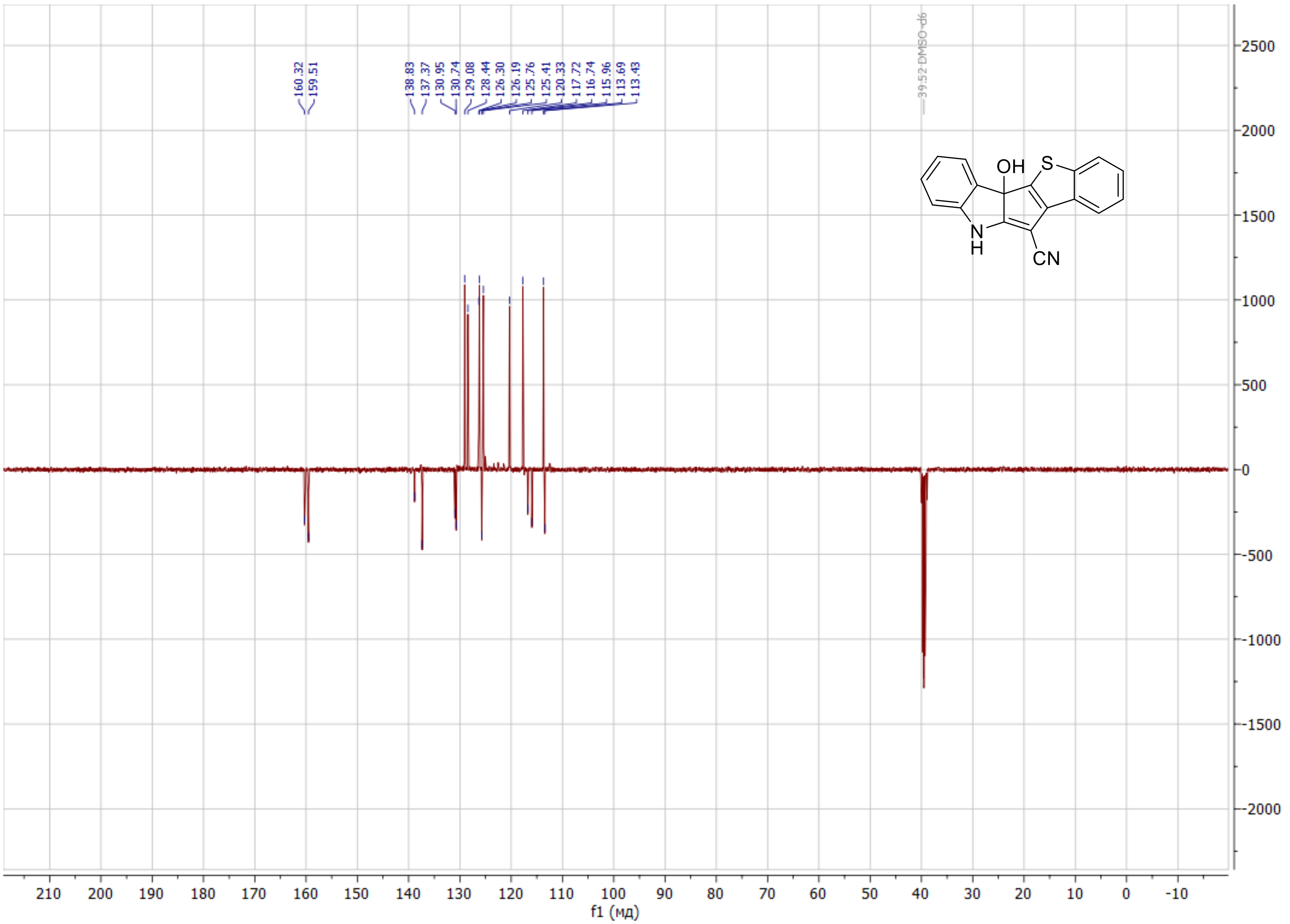

Figure S52. ^{13}C DEPTQ NMR spectrum of 2ap in DMSO-d_6 (100 MHz)

HRMS spectral charts

HRMS spectral charts for (E)-2-aryl-2-(3-oxoindolin-2-ylidene)acetonitriles (2aa-2cb)

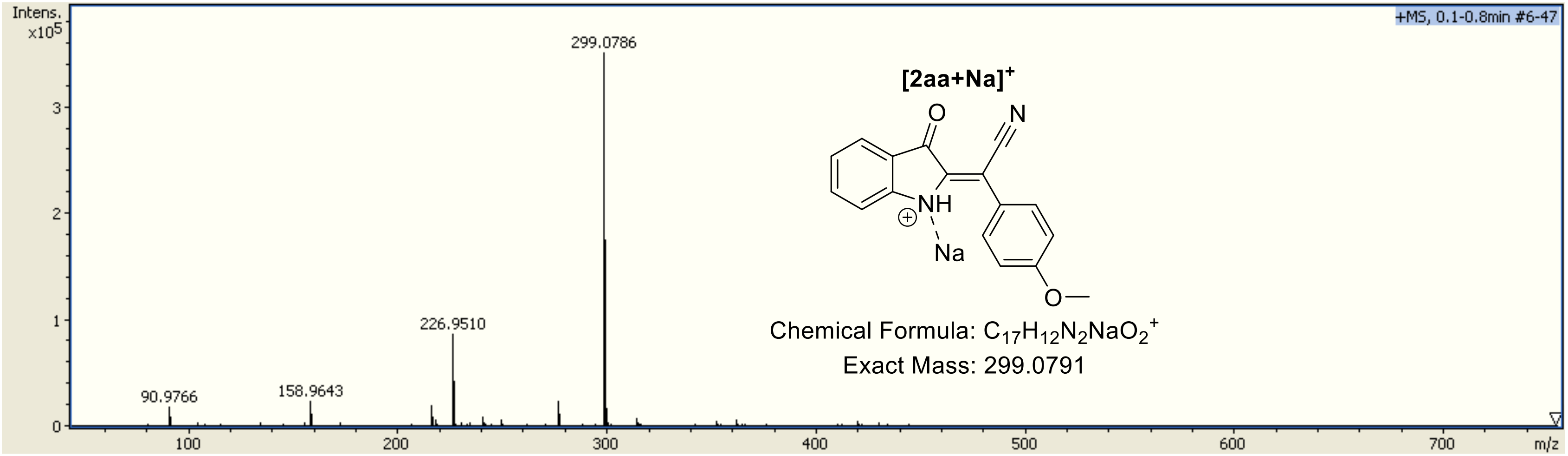

Figure S53. HRMS spectral chart for **2aa**

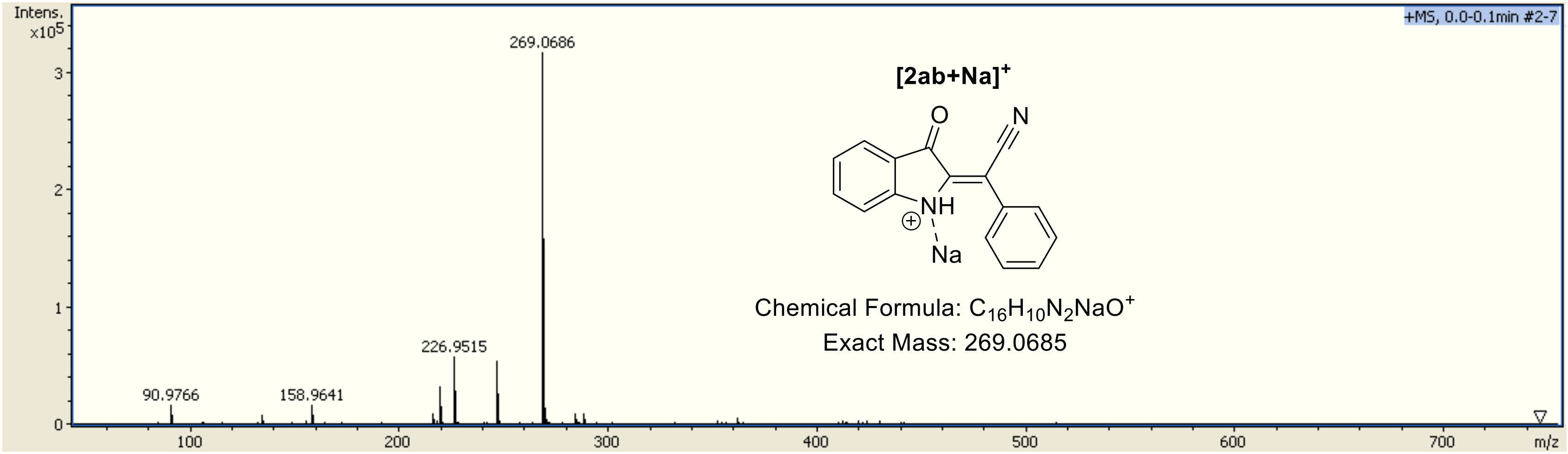

Figure S54. HRMS spectral chart for **2ab**

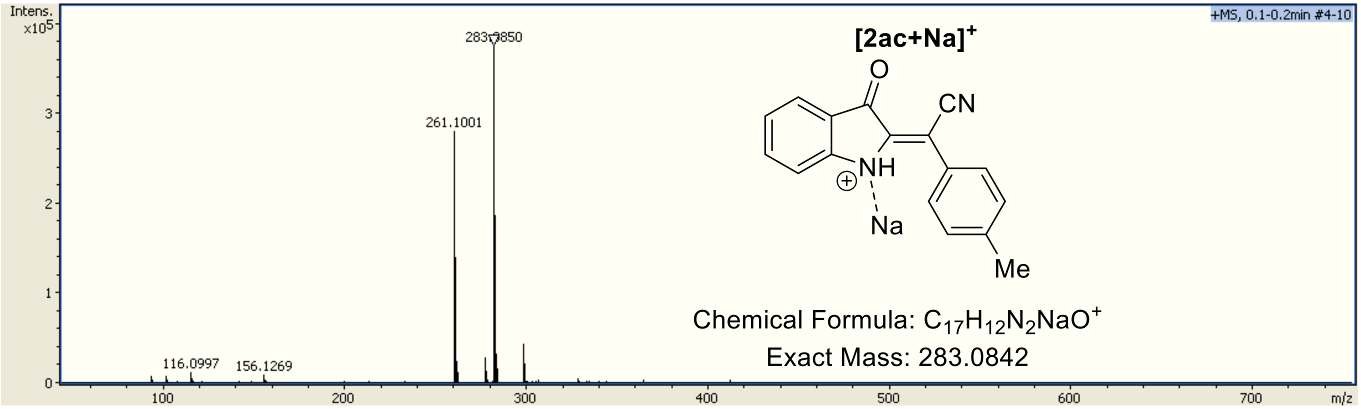

Figure S55. HRMS spectral chart for **2ac**

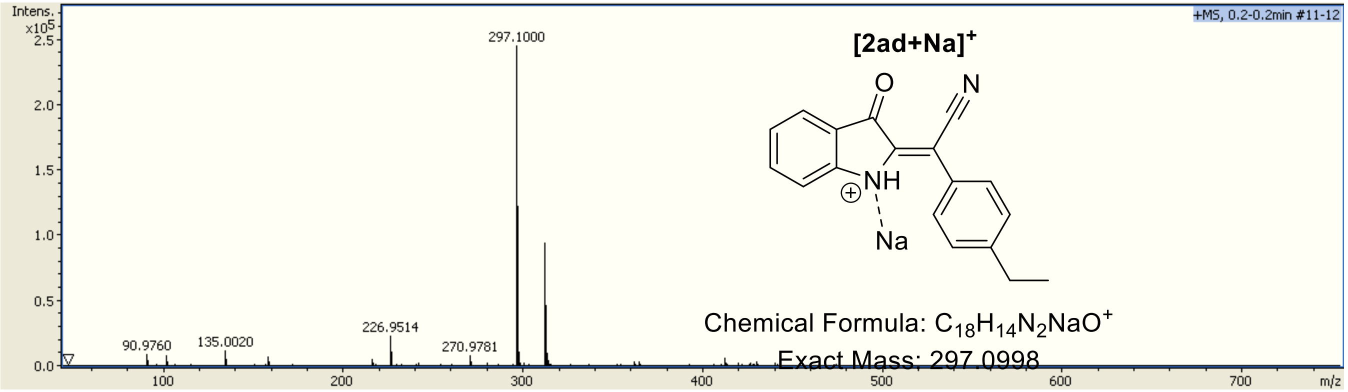

Figure S56. HRMS spectral chart for **2ad**

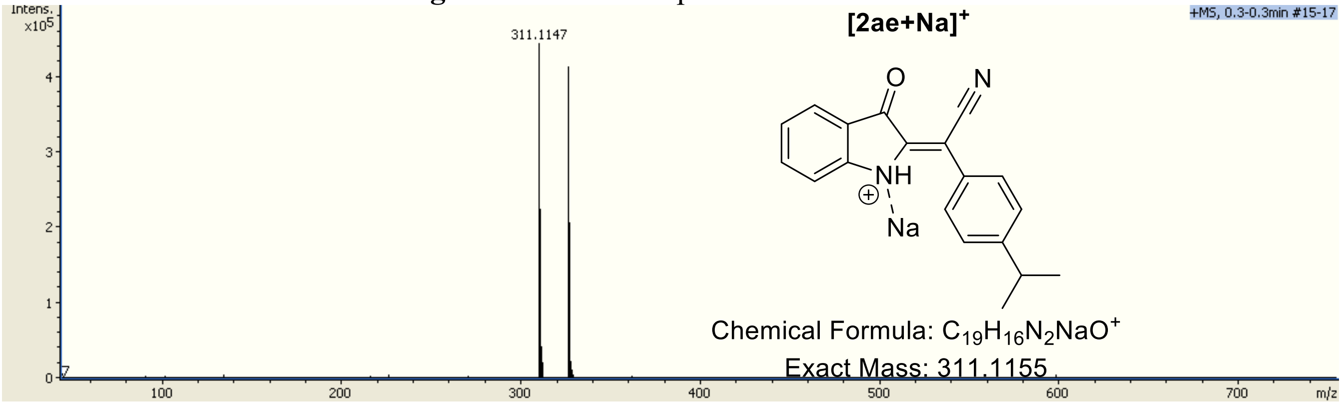

Figure S57. HRMS spectral chart for **2ae**

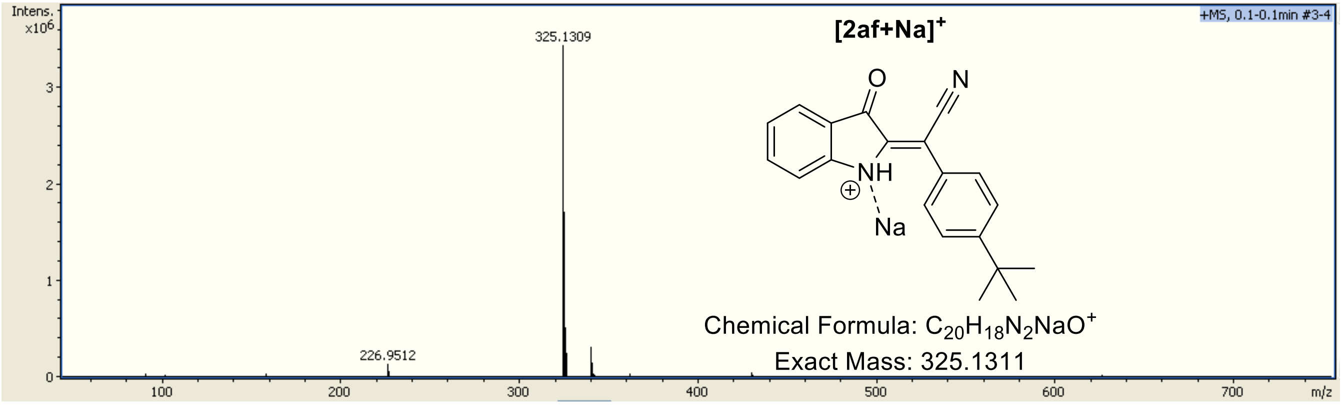

Figure S58. HRMS spectral chart for **2af**

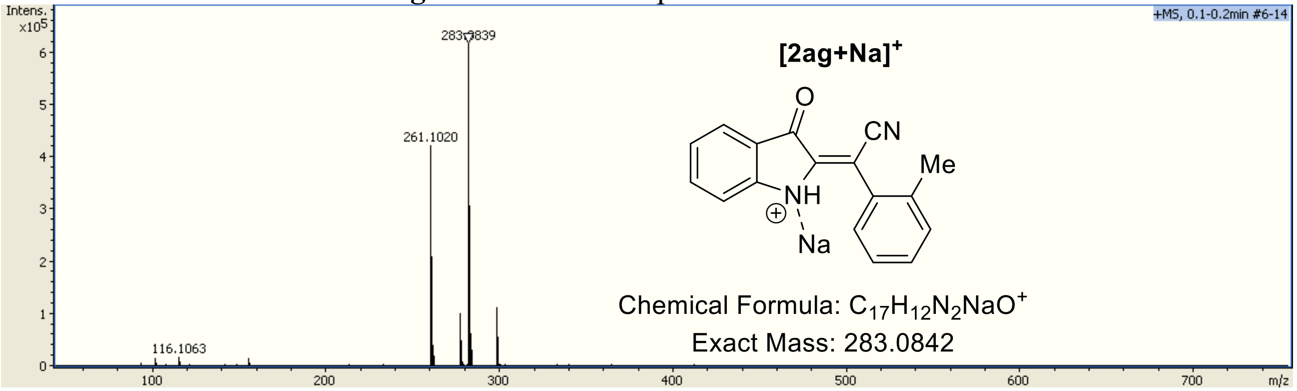

Figure S59. HRMS spectral chart for **2ag**

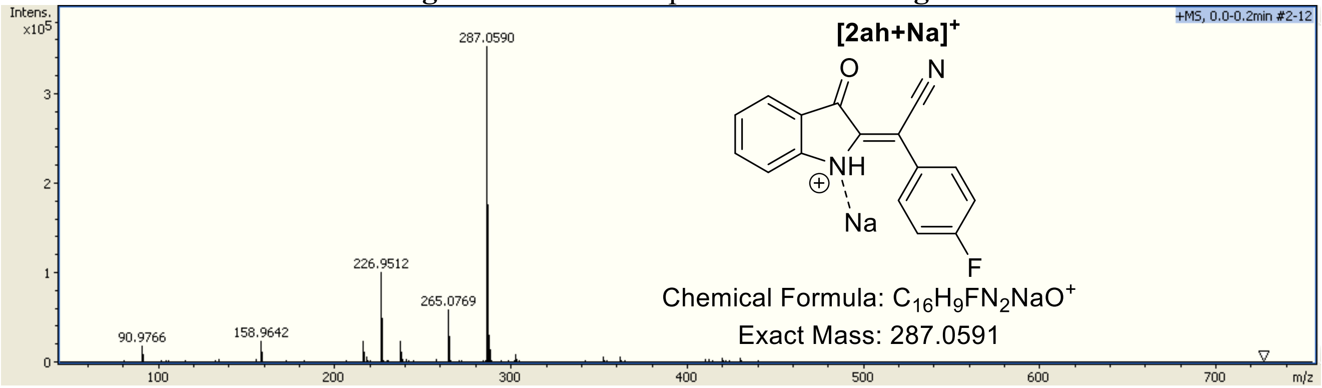

Figure S60. HRMS spectral chart for **2ah**

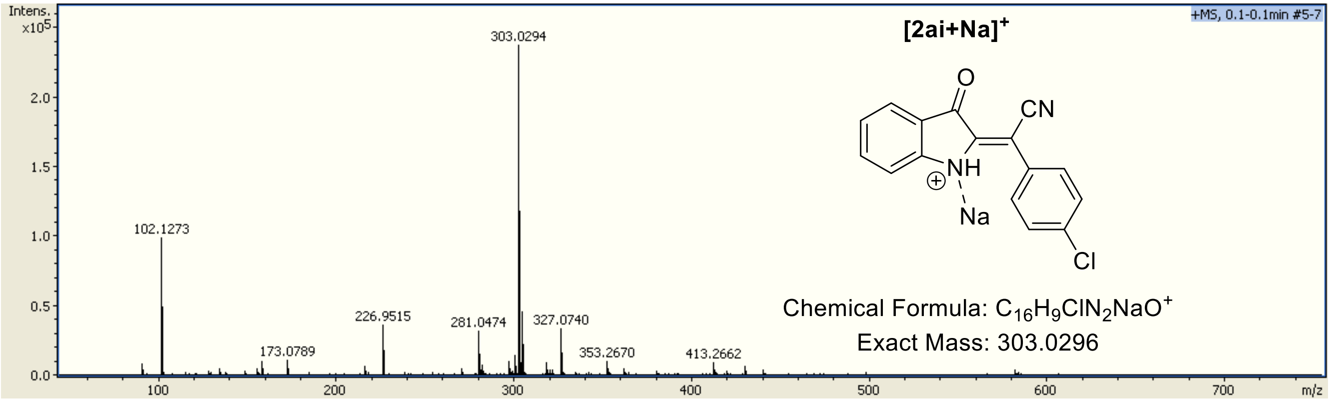

Figure S61. HRMS spectral chart for **2ai**

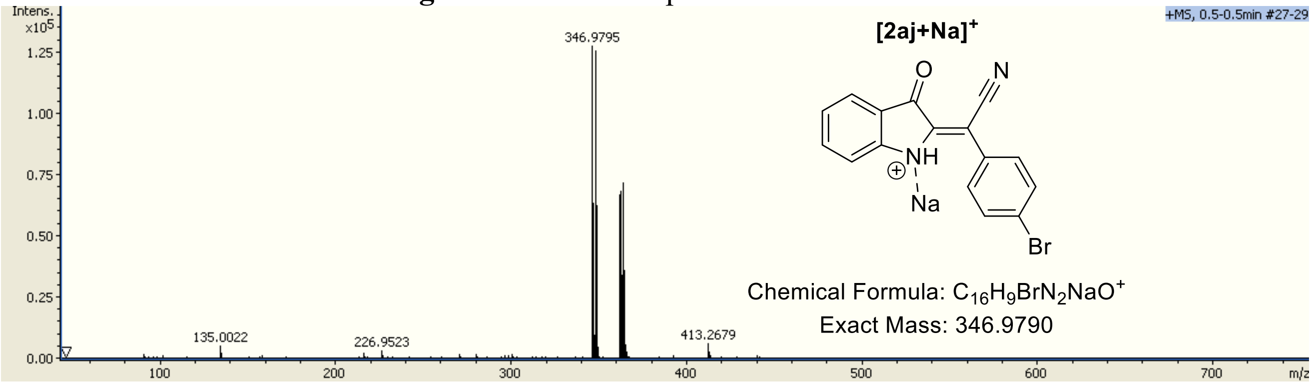

Figure S62. HRMS spectral chart for **2aj**

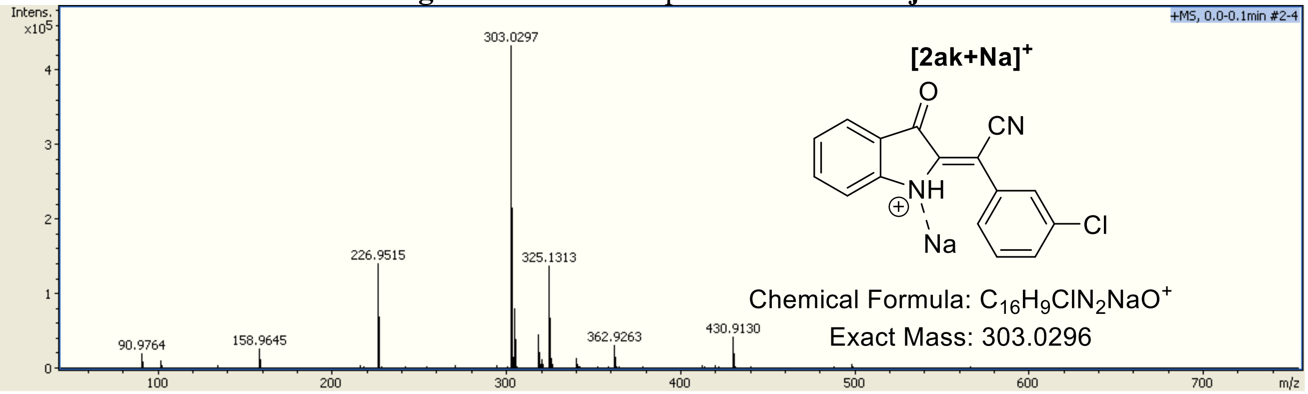

Figure S63. HRMS spectral chart for **2ak**

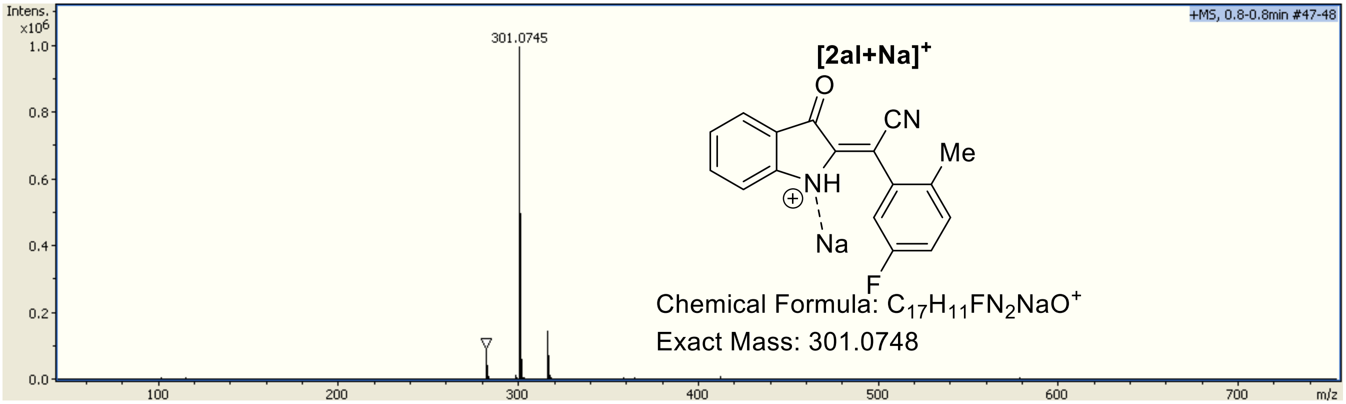

Figure S64. HRMS spectral chart for **2al**

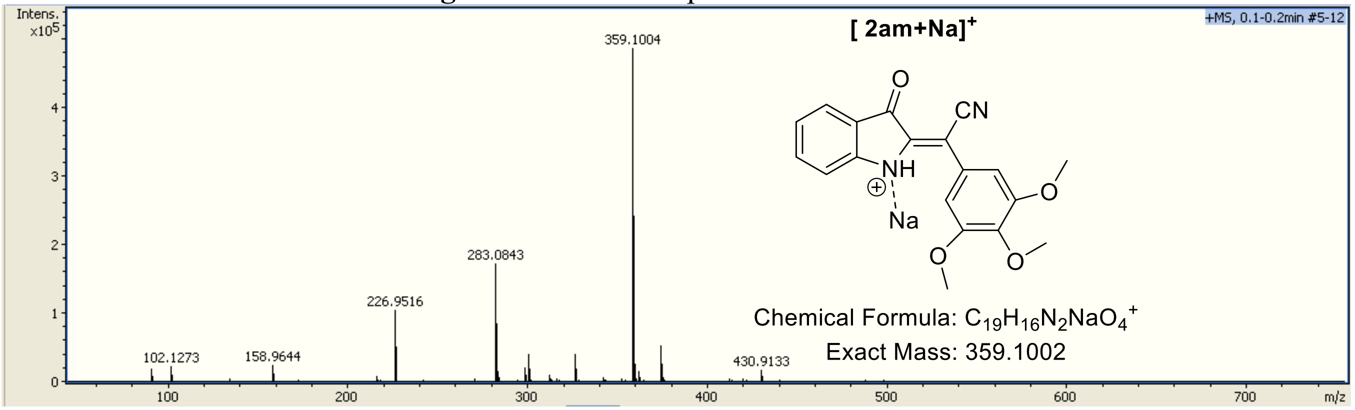

Figure S65. HRMS spectral chart for **2am**

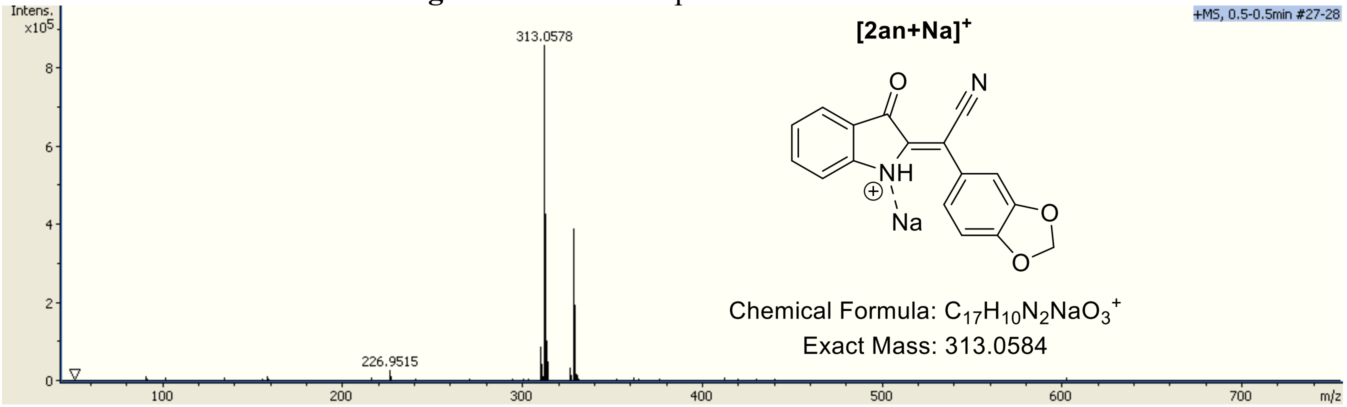

Figure S66. HRMS spectral chart for **2an**

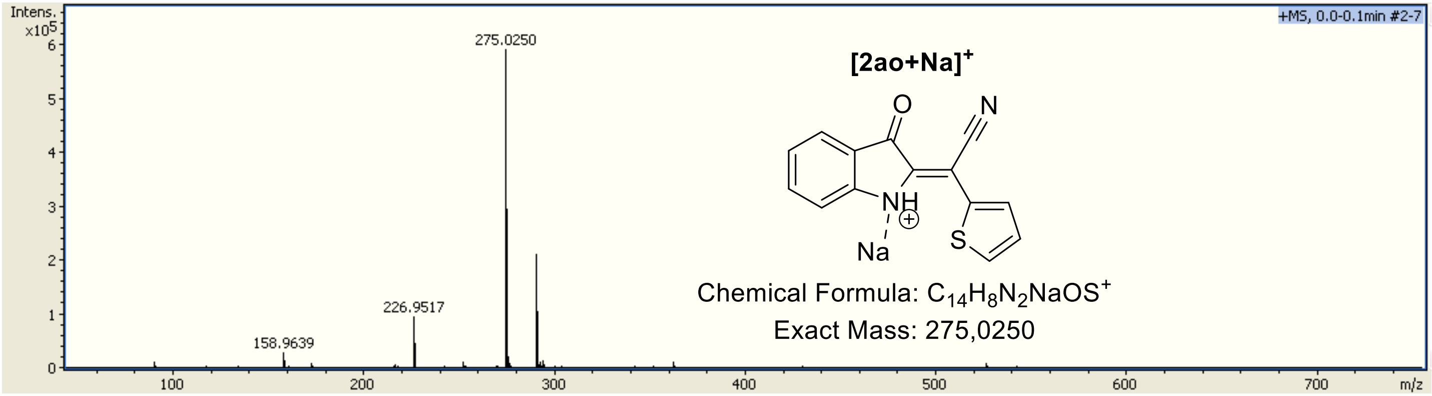

Figure S67. HRMS spectral chart for **2ao**

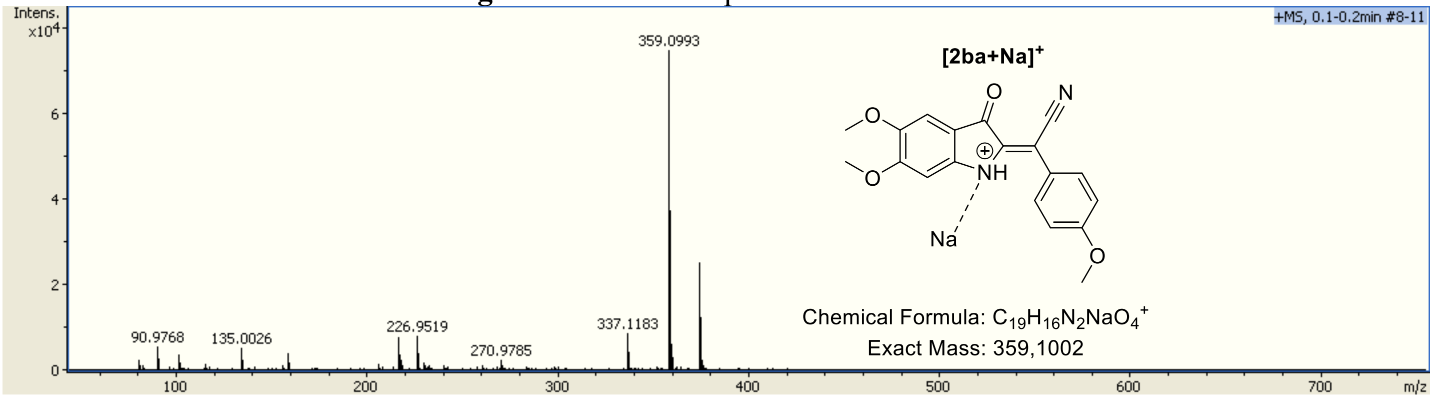

Figure S68. HRMS spectral chart for **2ba**

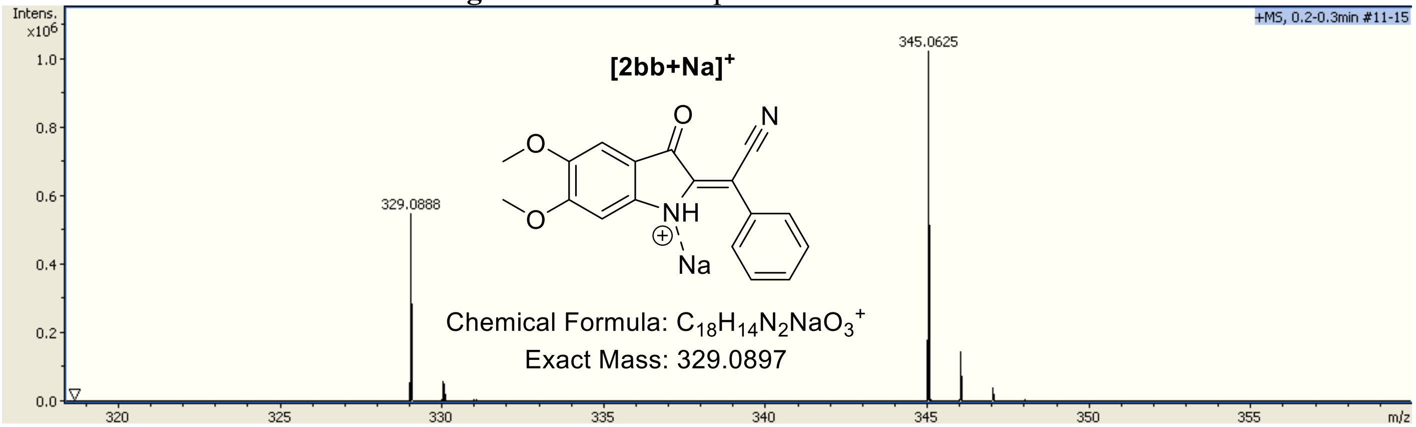

Figure S69. HRMS spectral chart for **2bb**

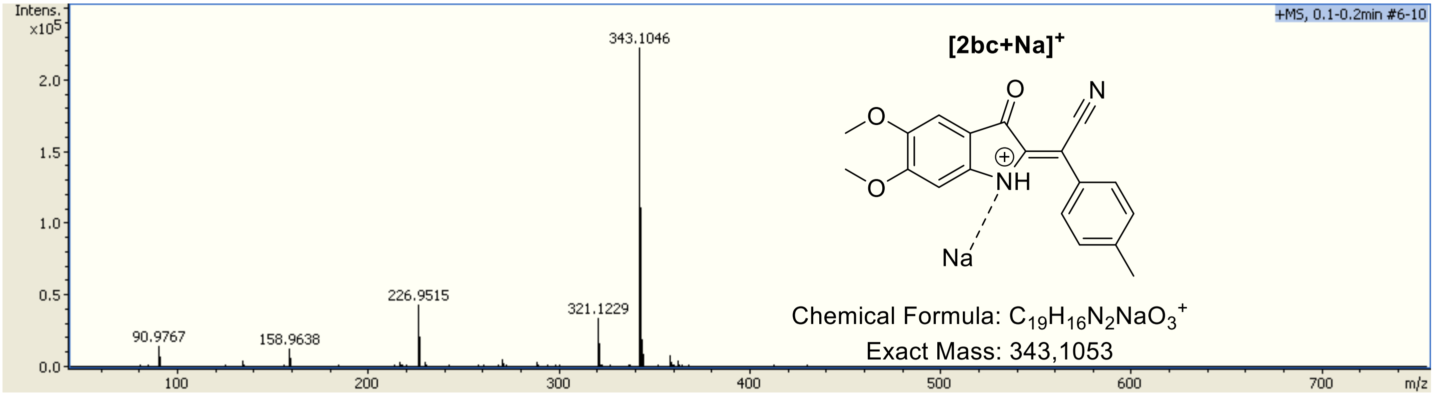

Figure S70. HRMS spectral chart for **2bc**

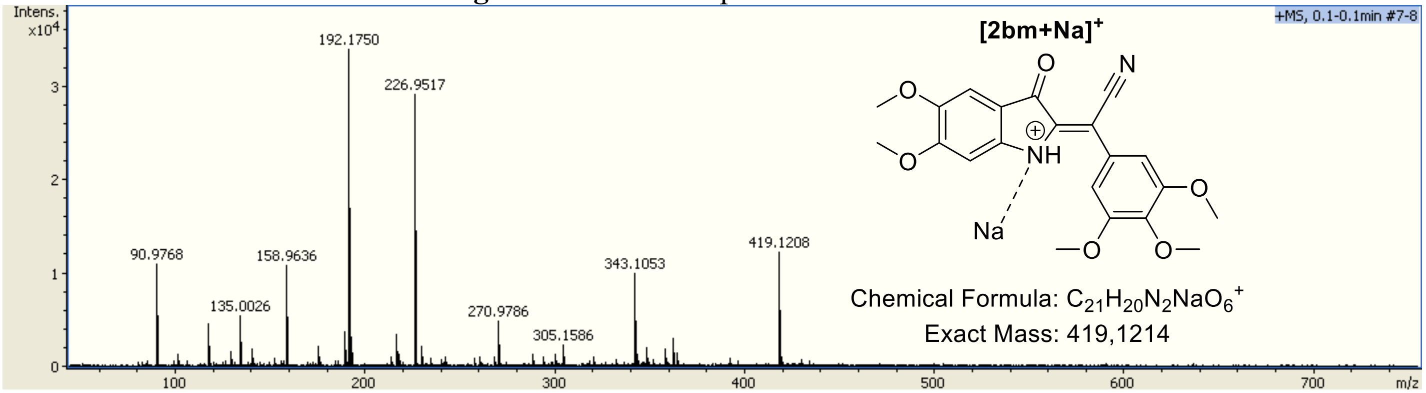

Figure S71. HRMS spectral chart for **2bm**

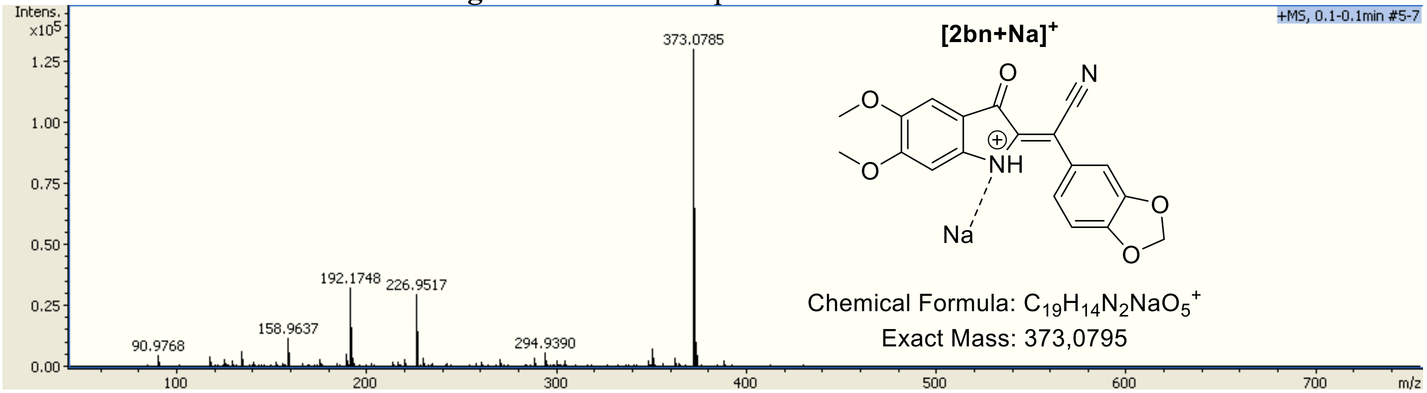

Figure S72. HRMS spectral chart for **2bn**

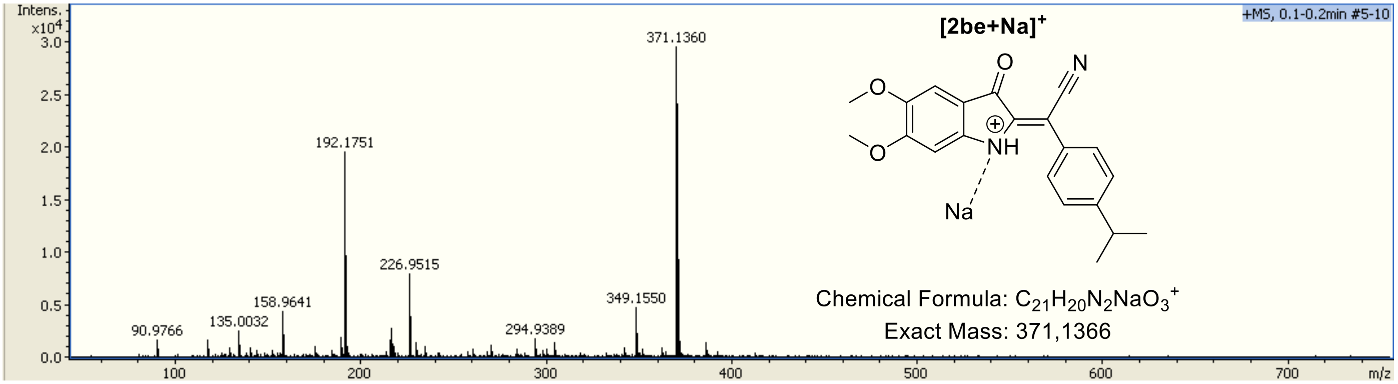

Figure S73. HRMS spectral chart for **2be**

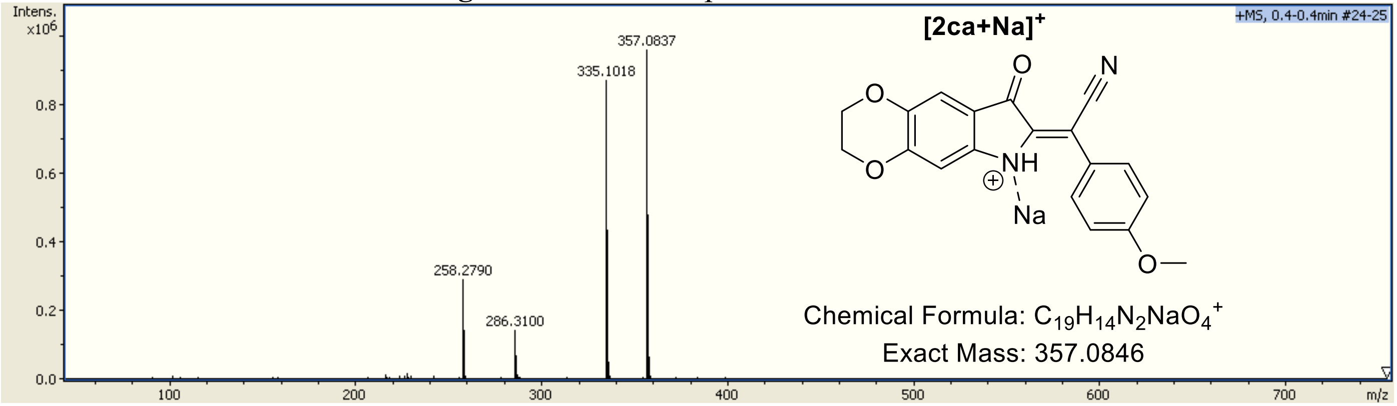

Figure S74. HRMS spectral chart for **2ca**

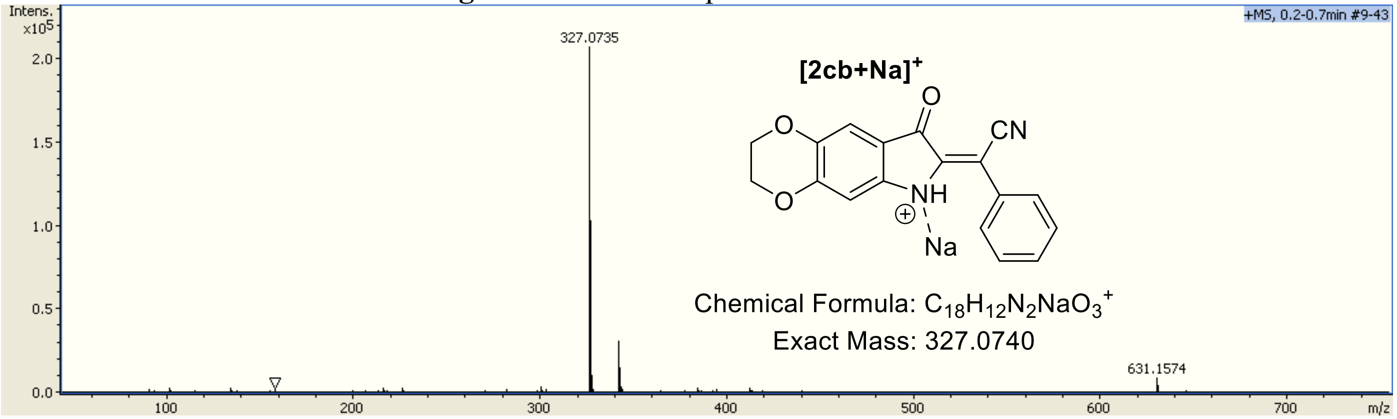

Figure S75. HRMS spectral chart for **2cb**

HRMS spectral charts for (*E*)-2-(1-hydroxy-3-oxoindolin-2-ylidene)-2-phenylacetonitrile (10ab):

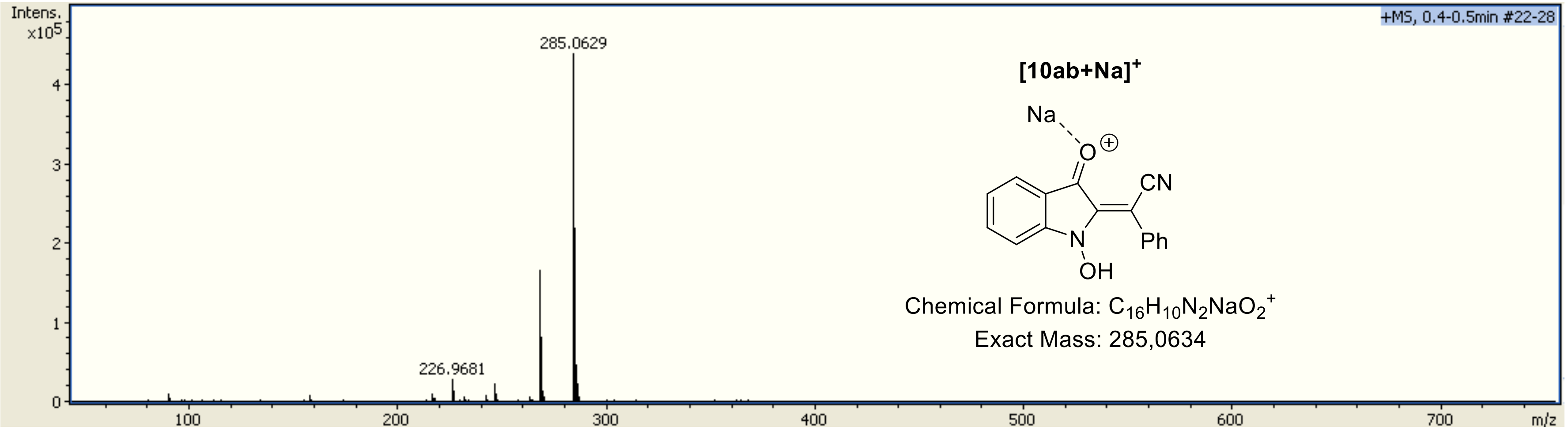

Figure S76. HRMS spectral chart for **2ab**

HRMS spectral charts for 4-oxo-2,4-diphenylbutanenitrile (12):

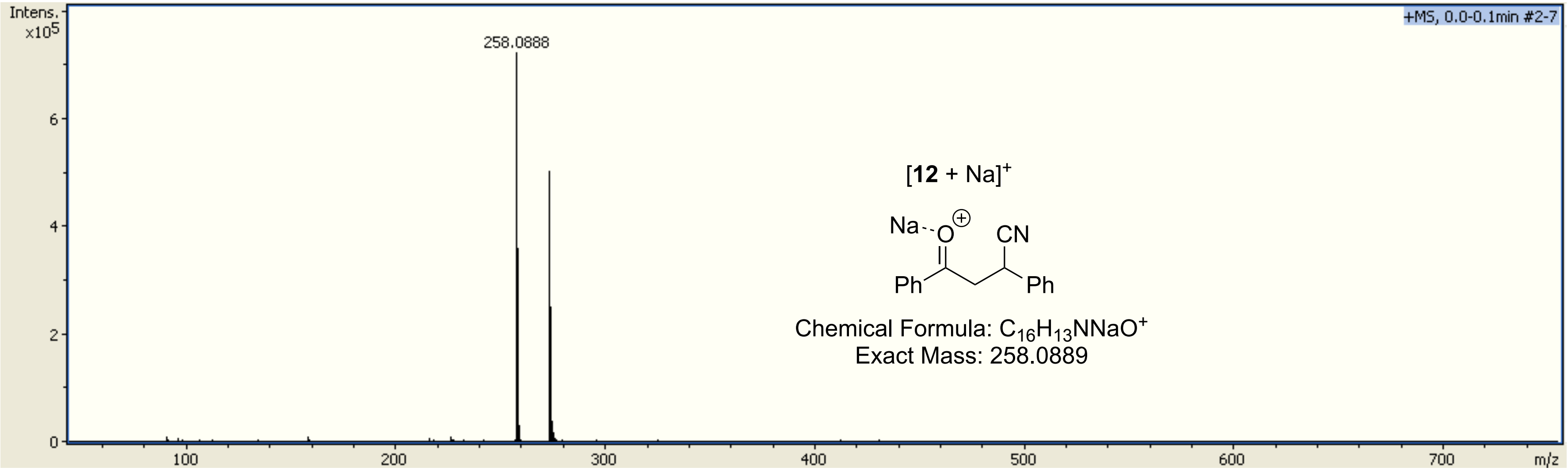

Figure S77. HRMS spectral chart for **12**

HRMS spectral charts for 5*b*-hydroxy-5*b*,10-dihydrobenzo[4',5']thieno[2',3':3,4]cyclopenta[1,2-*b*]indole-11-carbonitrile (2ap):

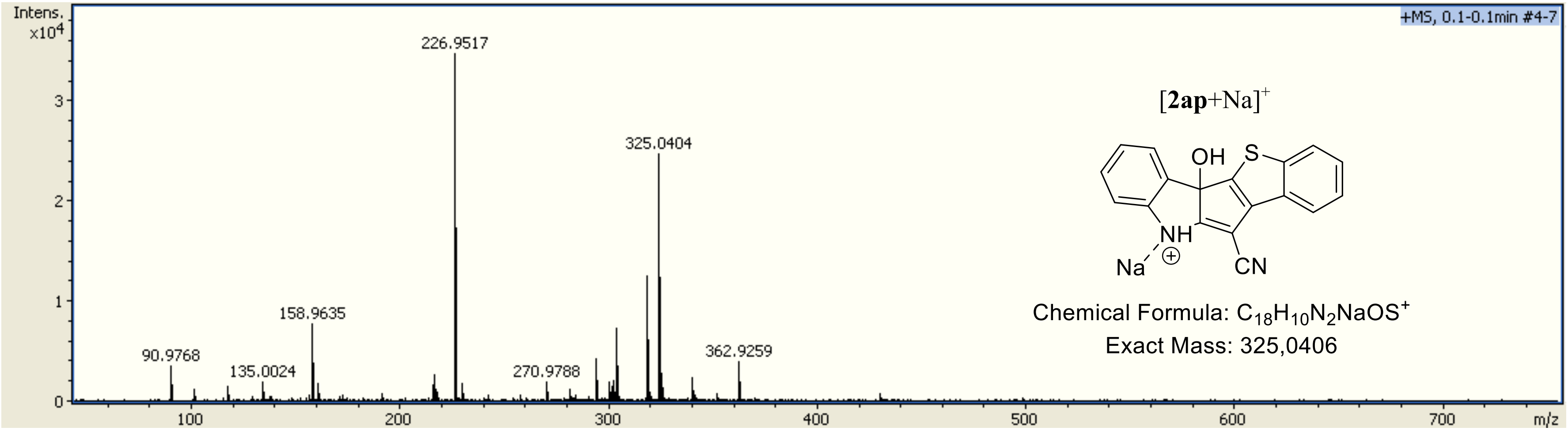

Figure S78. HRMS spectral chart for **2ap**

X-Ray crystallography data

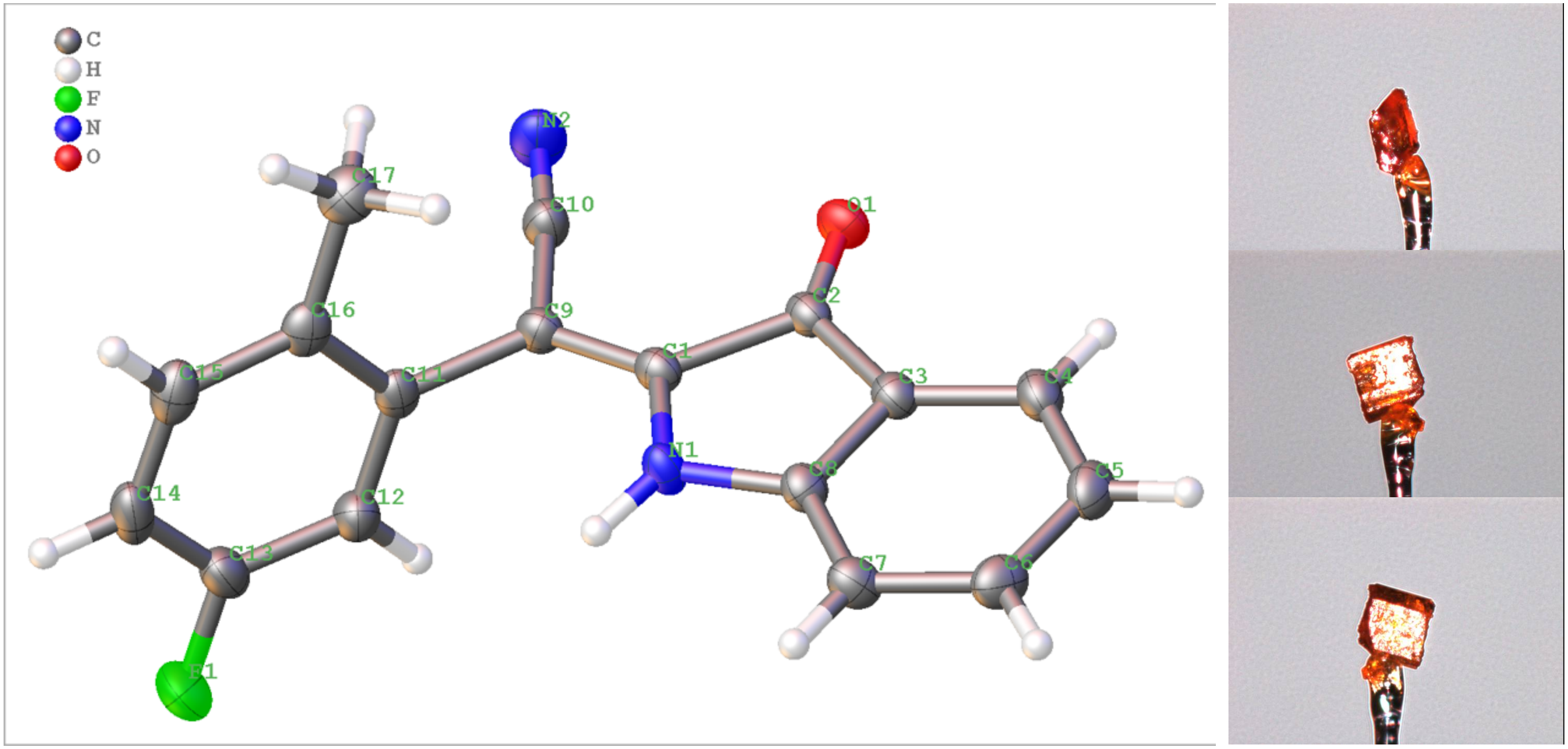

Figure S79. ORTEP drawing of the crystal structure showing 50% probability thermal ellipsoids (left, CCDC 2157035) and microphotography of the single crystal of compound **2al** used for X-Ray diffraction analysis at the bottom.

Table S1. Crystal data and structure refinement for 2al.

Identification code	ANNA_nik544_2
Empirical formula	$C_{17}H_{11}FN_2O$
Formula weight	278.28
Temperature/K	100.00(10)
Crystal system	orthorhombic
Space group	Pbca
a/Å	8.8927(3)
b/Å	12.5305(4)
c/Å	24.5955(10)
α/°	90
β/°	90
γ/°	90
Volume/Å^3	2740.68(17)
Z	8
ρ_{calc}g/cm^3	1.349
μ/mm^{-1}	0.783
F(000)	1152.0
Crystal size/mm^3	0.251 × 0.222 × 0.09
Radiation	Cu Kα (λ = 1.54184)
2Θ range for data collection/°	7.188 to 152.588
Index ranges	$-8 \le h \le 11, -15 \le k \le 15, -27 \le l \le 30$
Reflections collected	15288
Independent reflections	2868 [R_{int} = 0.0215, R_{sigma} = 0.0135]
Data/restraints/parameters	2868/0/195
Goodness-of-fit on F^2	1.070
Final R indexes [I>=2σ (I)]	R_1 = 0.0381, wR_2 = 0.0909
Final R indexes [all data]	R_1 = 0.0394, wR_2 = 0.0920
Largest diff. peak/hole / e Å^{-3}	0.24/-0.19

Table S2. Fractional Atomic Coordinates (×10^4) and Equivalent Isotropic Displacement Parameters (Å^2×10^3) for 2al. U_{eq} is defined as 1/3 of of the trace of the orthogonalised U_{IJ} tensor.

Atom	*x*	*y*	*z*	U(eq)
F1	-810.1(10)	2610.3(7)	6658.5(4)	45.8(2)
O1	3874.0(10)	7291.9(6)	5619.9(3)	26.53(19)
N1	4497.3(11)	4729.0(8)	6129.7(4)	23.3(2)
N2	723.5(14)	7457.4(9)	6346.5(5)	37.1(3)
C8	5723.8(12)	4846.8(9)	5779.7(5)	22.5(2)
C2	4347.2(13)	6409.5(9)	5728.5(4)	21.4(2)
C1	3603.8(13)	5611.9(9)	6114.1(4)	21.1(2)
C3	5695.8(13)	5853.7(9)	5534.9(5)	22.5(2)
C11	1666.4(13)	4895.8(9)	6755.5(5)	22.8(2)
C9	2300.6(13)	5741.5(9)	6389.7(4)	22.2(2)
C16	2012.7(13)	4895.5(9)	7310.9(5)	25.1(2)
C7	6840.9(13)	4108.4(10)	5666.4(5)	26.7(2)
C10	1472.6(14)	6710.9(9)	6347.3(5)	26.7(3)
C4	6803.4(13)	6152.9(10)	5165.3(5)	26.8(2)
C12	720.4(14)	4124.1(10)	6533.3(5)	28.4(3)
C15	1391.6(14)	4089.4(10)	7633.6(5)	30.7(3)
C6	7934.9(13)	4418.0(11)	5294.5(5)	29.8(3)
C14	444.3(15)	3322.1(10)	7420.1(6)	32.4(3)
C13	128.0(15)	3356.6(10)	6872.4(6)	31.6(3)
C5	7923.9(14)	5421.4(11)	5048.4(5)	30.6(3)
C17	2993.9(15)	5742.7(10)	7556.9(5)	31.8(3)

Table S3. Anisotropic Displacement Parameters (Å^2×10^3) for 2al. The Anisotropic displacement factor exponent takes the form: -2π^2[h^{2}a*^{2}U$_{11}$+2hka*b*U$_{12}$+…].

Atom	U_{11}	U_{22}	U_{33}	U_{23}	U_{13}	U_{12}
F1	51.8(5)	36.2(4)	49.3(5)	-3.9(4)	14.9(4)	-20.7(4)
O1	33.4(4)	18.5(4)	27.7(4)	4.2(3)	0.4(3)	1.3(3)
N1	21.6(5)	18.7(5)	29.6(5)	6.6(4)	4.4(4)	1.8(4)
N2	43.0(6)	24.7(5)	43.5(7)	5.4(5)	12.9(5)	9.9(5)
C8	19.7(5)	22.9(5)	24.9(5)	1.1(4)	0.0(4)	-2.1(4)
C2	25.0(5)	18.7(5)	20.5(5)	1.6(4)	-0.7(4)	-2.7(4)
C1	23.5(5)	18.0(5)	21.7(5)	2.3(4)	-0.9(4)	-0.1(4)
C3	22.6(5)	21.3(5)	23.6(5)	0.7(4)	0.0(4)	-2.6(4)
C11	22.1(5)	19.5(5)	26.7(5)	3.0(4)	6.1(4)	5.0(4)
C9	25.1(5)	18.3(5)	23.3(5)	1.7(4)	1.8(4)	1.7(4)
C16	21.4(5)	26.0(6)	27.8(6)	3.1(4)	2.8(4)	8.3(4)
C7	21.9(5)	24.1(6)	34.0(6)	0.8(5)	-0.5(5)	0.7(4)
C10	30.9(6)	22.0(6)	27.1(6)	2.5(4)	7.2(5)	1.5(5)
C4	27.6(6)	26.8(6)	26.0(5)	1.8(4)	2.0(5)	-6.8(5)
C12	33.0(6)	25.5(6)	26.7(6)	0.5(5)	6.7(5)	-2.0(5)
C15	29.9(6)	34.3(6)	27.9(6)	8.8(5)	6.4(5)	10.6(5)
C6	20.1(5)	33.4(6)	35.9(6)	-6.3(5)	1.8(5)	0.2(5)
C14	32.9(6)	27.1(6)	37.1(7)	10.0(5)	14.6(5)	5.3(5)
C13	32.1(6)	23.8(6)	39.0(7)	-0.7(5)	10.7(5)	-4.1(5)
C5	23.2(6)	37.9(7)	30.7(6)	-2.3(5)	5.3(5)	-7.0(5)
C17	31.1(6)	34.2(7)	30.1(6)	1.0(5)	-3.5(5)	4.9(5)

Table S4. Bond Lengths for 2al.

Atom	Atom	Length/Å	Atom	Atom	Length/Å
F1	C13	1.3591(15)	C11	C16	1.4004(17)
O1	C2	1.2128(14)	C11	C12	1.3934(17)
N1	C8	1.3972(15)	C9	C10	1.4244(16)
N1	C1	1.3626(14)	C16	C15	1.3982(17)
N2	C10	1.1483(16)	C16	C17	1.5015(18)
C8	C3	1.3982(16)	C7	C6	1.3905(18)
C8	C7	1.3859(16)	C4	C5	1.3842(18)
C2	C1	1.5283(15)	C12	C13	1.3777(17)
C2	C3	1.4663(16)	C15	C14	1.382(2)
C1	C9	1.3523(16)	C6	C5	1.3955(19)
C3	C4	1.3919(16)	C14	C13	1.377(2)
C11	C9	1.5002(15)			

Table S5. Bond Angles for 2al.

Atom	Atom	Atom	Angle/˚	Atom	Atom	Atom	Angle/˚
C1	N1	C8	110.62(9)	C1	C9	C10	120.58(10)
N1	C8	C3	110.29(10)	C10	C9	C11	116.88(10)
C7	C8	N1	127.80(11)	C11	C16	C17	121.37(11)
C7	C8	C3	121.91(11)	C15	C16	C11	117.84(12)
O1	C2	C1	125.65(10)	C15	C16	C17	120.78(11)
O1	C2	C3	130.19(11)	C8	C7	C6	116.59(11)
C3	C2	C1	104.16(9)	N2	C10	C9	174.10(13)
N1	C1	C2	107.23(9)	C5	C4	C3	117.82(11)
C9	C1	N1	125.67(10)	C13	C12	C11	118.54(12)
C9	C1	C2	127.10(10)	C14	C15	C16	121.86(12)
C8	C3	C2	107.66(10)	C7	C6	C5	122.13(11)
C4	C3	C8	120.78(11)	C13	C14	C15	118.32(11)
C4	C3	C2	131.52(11)	F1	C13	C12	118.73(12)
C16	C11	C9	120.17(11)	F1	C13	C14	118.85(11)
C12	C11	C9	118.81(10)	C14	C13	C12	122.42(12)
C12	C11	C16	121.01(11)	C4	C5	C6	120.77(11)
C1	C9	C11	122.54(10)				

Table S6. Torsion Angles for 2al.

A	B	C	D	Angle/°	A	B	C	D	Angle/°
O1	C2	C1	N1	-179.18(11)	C3	C2	C1	C9	-178.55(11)
O1	C2	C1	C9	1.12(19)	C3	C4	C5	C6	0.02(18)
O1	C2	C3	C8	-179.45(12)	C11	C16	C15	C14	-1.09(17)
O1	C2	C3	C4	-1.7(2)	C11	C12	C13	F1	179.19(11)
N1	C8	C3	C2	-1.49(13)	C11	C12	C13	C14	-0.40(19)
N1	C8	C3	C4	-179.53(11)	C9	C11	C16	C15	179.99(10)
N1	C8	C7	C6	179.14(12)	C9	C11	C16	C17	1.15(16)
N1	C1	C9	C11	-0.20(18)	C9	C11	C12	C13	-179.28(11)
N1	C1	C9	C10	179.03(11)	C16	C11	C9	C1	92.36(14)
C8	N1	C1	C2	-2.11(13)	C16	C11	C9	C10	-86.89(14)
C8	N1	C1	C9	177.60(11)	C16	C11	C12	C13	0.04(18)
C8	C3	C4	C5	0.18(17)	C16	C15	C14	C13	0.74(18)
C8	C7	C6	C5	0.39(18)	C7	C8	C3	C2	177.94(11)
C2	C1	C9	C11	179.45(11)	C7	C8	C3	C4	-0.10(18)
C2	C1	C9	C10	-1.32(18)	C7	C6	C5	C4	-0.32(19)
C2	C3	C4	C5	-177.33(12)	C12	C11	C9	C1	-88.31(14)
C1	N1	C8	C3	2.33(13)	C12	C11	C9	C10	92.44(13)
C1	N1	C8	C7	-177.06(12)	C12	C11	C16	C15	0.68(16)
C1	C2	C3	C8	0.21(12)	C12	C11	C16	C17	-178.16(11)
C1	C2	C3	C4	177.96(12)	C15	C14	C13	F1	-179.57(11)
C3	C8	C7	C6	-0.18(17)	C15	C14	C13	C12	0.02(19)
C3	C2	C1	N1	1.15(12)	C17	C16	C15	C14	177.76(11)

Table S7. Hydrogen Atom Coordinates (Å×10[4]) and Isotropic Displacement Parameters (Å[2]×10[3]) for 2al.

Atom	*x*	*y*	*z*	U(eq)
H7	6858.07	3440.02	5830.67	32
H4	6791.01	6822.49	5002.25	32
H12	493.86	4127.03	6164.17	34
H15	1622.6	4068.99	8002.19	37
H6	8698.63	3940.97	5206.99	36
H14	30.86	2795.76	7640.81	39
H5	8678.17	5600.62	4802.89	37
H17A	3947.89	5751.76	7374.22	48
H17B	3144.44	5592.44	7935.72	48
H17C	2516.88	6425.75	7517.94	48
H1	4250(18)	4091(13)	6269(7)	35(4)

Experimental

Single crystals of $C_{17}H_{11}FN_2O$ **2al** were crystallized by slow evaporation of saturated solution in EtOAc. A suitable crystal was selected and mounted on the glass stick by acrylic glue on a SuperNova, Dual, Cu at home/near, AtlasS2 diffractometer. The crystal was kept at 100.00(10) K during data collection. Using Olex2 [S1], the structure was solved with the SHELXT [S2] structure solution program using Intrinsic Phasing and refined with the SHELXL [S3] refinement package using Least Squares minimisation.

Crystal structure determination of 2al

Crystal Data for $C_{17}H_{11}FN_2O$ (M =278.28 g/mol): orthorhombic, space group Pbca (no. 61), a = 8.8927(3) Å, b = 12.5305(4) Å, c = 24.5955(10) Å, V = 2740.68(17) Å^3, Z = 8, T = 100.00(10) K, μ(Cu Kα) = 0.783 mm^{-1}, $Dcalc$ = 1.349 g/cm^3, 15288 reflections measured (7.188° ≤ 2Θ ≤ 152.588°), 2868 unique (R_{int} = 0.0215, R_{sigma} = 0.0135) which were used in all calculations. The final R_1 was 0.0381 (I > 2σ(I)) and wR_2 was 0.0920 (all data).

Refinement model description

Number of restraints - 0, number of constraints - unknown.

Details:

```
1. Fixed Uiso
 At 1.2 times of:
  All C(H) groups
 At 1.5 times of:
  All C(H,H,H) groups
2.a Aromatic/amide H refined with riding coordinates:
 C7(H7), C4(H4), C12(H12), C15(H15), C6(H6), C14(H14), C5(H5)
2.b Idealised Me refined as rotating group:
 C17(H17A,H17B,H17C)
```

This report has been created with Olex2, compiled on 2020.11.12 svn.r5f609507 for OlexSys. Please let us know if there are any errors or if you would like to have additional features.

References

S1. Dolomanov, O.V., Bourhis, L.J., Gildea, R.J, Howard, J.A.K. & Puschmann, H. (2009), *J. Appl. Cryst*. 42, 339-341.

S2. Sheldrick, G.M. (2008). *Acta Cryst*. A64, 112-122.

S3. Sheldrick, G.M. (2015). *Acta Cryst*. C71, 3-8.
